# The structures of eleven (4-phen­yl)piperazinium salts containing organic anions

**DOI:** 10.1107/S2056989022009057

**Published:** 2022-09-22

**Authors:** Sreeramapura D. Archana, Haruvegowda Kiran Kumar, Hemmige S. Yathirajan, Sabine Foro, Ray J. Butcher

**Affiliations:** a Department of Studies in Chemistry, University of Mysore, Manasagangotri, Mysore-570 006, India; b Institute of Materials Science, Darmstadt University of Technology, Alarich-Weiss-Strasse 2, D-64287 Darmstadt, Germany; c Department of Chemistry, Howard University, 525 College Street NW, Washington DC 20059, USA; University of Aberdeen, Scotland

**Keywords:** crystal structure, phenyl­piperazinium cation, organic salt

## Abstract

Eleven (4-phen­yl)piperazinium salts containing organic anions have been prepared and structurally characterized, namely, 4-phenyl­piperazin-1-ium 4-fluoro­benzoate monohydrate, C_10_H_15_N_2_^+^·C_7_H_4_FO_2_^−^·H_2_O, **1**; 4-phenyl­piperazin-1-ium 4-bromo­benzoate monohydrate, C_10_H_15_N_2_^+^·C_7_H_4_BrO_2_^−^·H_2_O, **3**; 4-phenyl­piperazin-1-ium 4-iodo­benzoate, C_10_H_15_N_2_^+^·C_7_H_4_IO_2_^−^, **4**; 4-phenyl­piperazin-1-ium 4-nitro­benzoate, C_10_H_15_N_2_^+^·C_7_H_4_NO_4_^−^, **5**; 4-phenyl­piperazin-1-ium 3,5-di­nitro­salicylate, C_10_H_15_N_2_^+^·C_7_H_3_N_2_O_7_^−^, **6**; 4-phenyl­piperazin-1-ium 3,5-di­nitro­benzoate, C_10_H_15_N_2_^+^·C_7_H_3_N_2_O_6_^−^, **7**; 4-phenyl­piperazin-1-ium picrate, C_10_H_15_N_2_^+^·C_6_H_2_N_3_O_7_^−^, **8**; 4-phenyl­piperazin-1-ium benzoate monohydrate, C_10_H_15_N_2_^+^·C_7_H_5_O_2_^−^·H_2_O, **9**; 4-phenyl­piperazin-1-ium *p*-toluene­sulfonate, C_10_H_15_N_2_^+^·C_7_H_7_O_3_S^−^, **10**; 4-phenyl­piperazin-1-ium tartarate monohydrate, C_10_H_15_N_2_^+^·C_4_H_5_O_6_^−^·H_2_O, **11**; and 4-phenyl­piperazin-1-ium fumarate, C_10_H_15_N_2_^+^·C_4_H_3_O_4_^−^, **12**. Compounds **1** and **3**–**12** are all 1:1 salts with the acid proton transferred to the phenyl­piperaizine basic N atom (the secondary amine) with the exception of **3** where there is disorder in the proton position with it being 68% attached to the base and 32% attached to the acid. Of the structures with similar stoichiometries only **3** and **9** are isomorphous. The 4-phenyl substituent in all cases occupies an equatorial position except for **12** where it is in an axial position. The crystal chosen for structure **7** was refined as a non-merohedral twin. There is disorder in **5**, **6**, **10** and **11**. For both **5** and **6**, a nitro group is disordered and was modeled with two equivalent orientations with occupancies of 0.62 (3)/0.38 (3) and 0.690 (11)/0.310 (11), respectively. For **6**, **10** and **11**, this disorder is associated with the phenyl ring of the phenyl­piperazinium cation with occupancies of 0.687 (10)/0.313 (10), 0.51 (7)/0.49 (7) and 0.611 (13)/389 (13), respectively. For all salts, the packing is dominated by the N—H⋯O hydrogen bonds formed by the cation and anion. In addition, several structures contain C—H⋯π (**1**, **3**, **4**, **8**, **9**, **10**, and **12**) and aromatic π–π stacking inter­actions (**6** and **8**) and one structure (**5**) contains a –NO_2_⋯π inter­action. For all structures, the Hirshfeld surface fingerprint plots show the expected prominent spikes as a result of the N—H⋯O and O—H⋯O hydrogen bonds.

## Abstract 2

Eleven (4-phen­yl)piperazinium salts containing organic anions have been prepared and structurally characterized.

## 1. Chemical context

The pharmacological properties of phenyl­piperazines and their derivatives have been described by various researchers (Cohen *et al.* 1982 ▸; Conrado *et al.* 2010 ▸; Neves *et al.* 2003 ▸; Hanano *et al.* 2000 ▸). The design and synthesis of phenyl­piperazine derivatives as potent anti­cancer agents for prostate cancer have been reported (Demirci *et al.*, 2019 ▸). Many pharmaceutical compounds are derived from 1-phenyl­piperazine, including oxypertine (Archer *et al.*, 1962 ▸), trazodone (Alhaider, 1992 ▸) and nefazodone. Derivatives of 1-phenyl­piperazine have shown other inter­esting properties, such as (C_10_H_15_N_2_)^+^_4_(Pb_3_Cl_10_)^4–^ where dielectric relaxation spectroscopy has shown different mol­ecular motions and measurements of AC conductivity as a function of frequency at different temperatures indicated a hopping conduction mechanism (Mathlouthi *et al.*, 2017 ▸) and new organic–inorganic hybrid materials of formula (C_10_H_15_N_2_)_7_(Sb_2_Cl_10_)(Sb_2_Cl_9_)(SbCl_5_)_2_(SbCl_4_)_2_Cl·7H_2_O (Lah­bib *et al.*, 2017 ▸). 
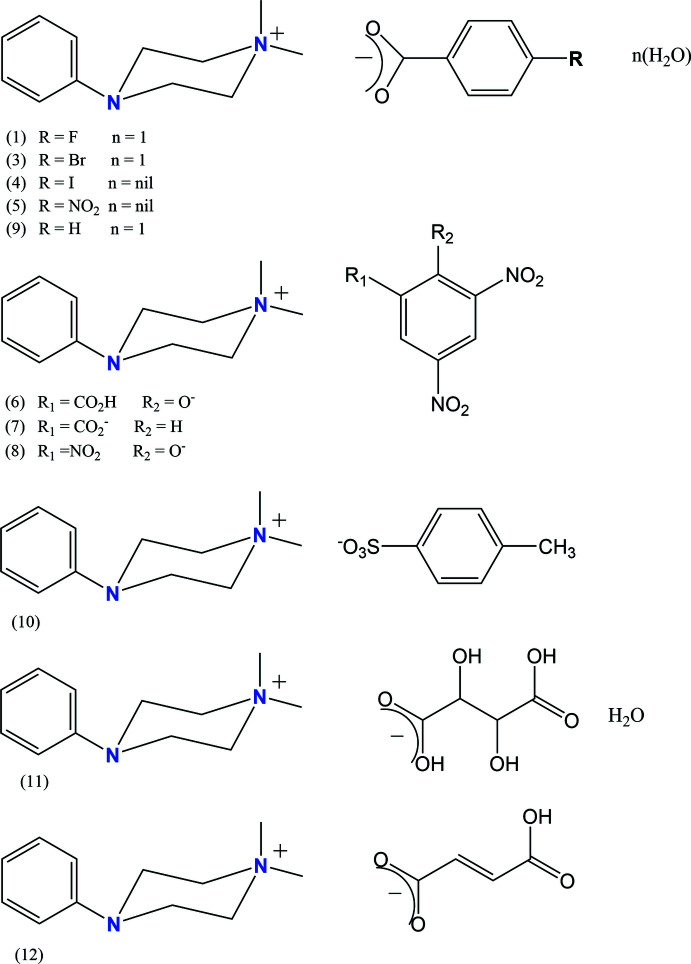


As part of our ongoing studies of hydrogen-bonding patterns in mol­ecular salts (Sagar *et al.*, 2017 ▸; Kiran Kumar *et al.*, 2019*a* ▸,*b* ▸, 2020 ▸, Harish Chinthal *et al.*, 2020 ▸), the present paper reports the syntheses and crystal structures of eleven mol­ecular salts of 1-phenyl­piperazine, C_10_H_14_N_2_, *viz*.: 4-phen­yl­piperazin-1-ium 4-fluoro­benzoate monohydrate, C_10_H_15_N_2_^+^·C_7_H_4_FO_2_^−^·H_2_O, **1**; phenyl­piperazin-1-ium 4-bro­mo­benzoate monohydrate, C_10_H_15_N_2_^+^·C_7_H_4_BrO_2_^−^·H_2_O, **3**; phenyl­piperazin-1-ium 4-iodo­benzoate, C_10_H_15_N_2_^+^·C_7_H_4_IO_2_^−^, **4**; phenyl­piperazin-1-ium 4-nitro­benzoate, C_10_H_15_N_2_^+^·C_7_H_4_NO_4_^−^, **5**; phenyl­piperazin-1-ium 3,5-di­nitro­salicylate, C_10_H_15_N_2_^+^·C_7_H_3_N_2_O_7_^−^, **6**; phenyl­piperazin-1-ium 3,5-di­nitro­benzoate, C_10_H_15_N_2_^+^·C_7_H_3_N_2_O_6_^−^, **7**; phenyl­piperazin-1-ium picrate, C_10_H_15_N_2_^+^·C_6_H_2_N_3_O_7_^−^, **8**; phenyl­piperazin-1-ium benzoate monohydrate, C_10_H_15_N_2_^+^·C_7_H_5_O_2_^−^·H_2_O, **9**; phenyl­piperazin-1-ium *p*-toluene­sulfonate, C_10_H_15_N_2_^+^·C_7_H_7_O_3_S^−^, **10**; phenyl­piperazin-1-ium tartarate monohydrate, C_10_H_15_N_2_^+^·C_4_H_5_O_6_^−^·H_2_O, **11**; and phenyl­piperazin-1-ium fumarate, C_10_H_15_N_2_^+^·C_4_H_3_O_4_^−^, **12**.

## 2. Structural commentary

**Figure 1:**
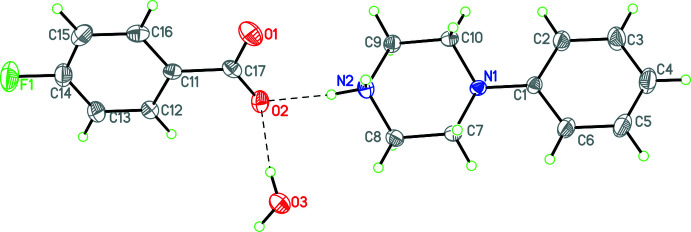
The mol­ecular structure of **1** with hydrogen bonds shown as dashed lines. Atomic displacement parameters are at the 30% probability level.

Compounds **1** and **3**–**12** (Figs. 1 ▸–11 ▸ ▸ ▸ ▸ ▸ ▸ ▸ ▸ ▸ ▸) are all 1:1 mol­ecular salts with the acid proton transferred to the secondary N atom of the phenyl­piperaizine base with the exception of **3** where there is disorder in the proton position with it being 68% attached to the base and 32% attached to the acid. Compounds **1**, **3** and **9** crystallize as mono-hydrates but the remaining crystals are solvent free. In compounds **1**, **3**, **4**, **5** and **9**, the anions are all benzoate ions or *p*-substituted benzoates but only **3** and **9** are isomorphous. Compounds **6**, **7** and **8** contain picrate or nitrated benzoate anions while **10** contains a tosyl­ate anion and **11** and **12** contain hydrogen tartarate and hydrogen fumarate mono-anions. Apart from the disorder in the acidic proton position mentioned above, there is disorder in **5**, **6**, **10** and **11**. For **5** this disorder is confined to the nitro substituent on the benzoate anion, which is disordered over two orientations with occupancies of 0.62 (3)/0.38 (2). For **6**, **10** and **11** the disorder is associated with the phenyl ring of the phenyl­piperazinium cation, with occupancies of 0.687 (10)/0.313 (10), 0.51 (7)/0.49 (7) and 0.611 (13)/389 (13), respectively. This is a common feature of this moiety as shown in a recent study (Kiran Kumar *et al.*, 2019*a* ▸) of 12 salts of the 4-meth­oxy­phenyl­piperazinium cation, of which four were found to contain similar disorder of the phenyl ring.

For the structures containing benzoate or *p*-substituted benzoate anions, the C—O distances fall into two groups. In one group (**3**, **5**), these distances are the same within experimental error at 2.246 (4) Å, while in the second group (**1**, **4**, and **9**) these are substanti­ally different and average 2.235 (4) and 2.255 (4) Å.

**Figure 5:**
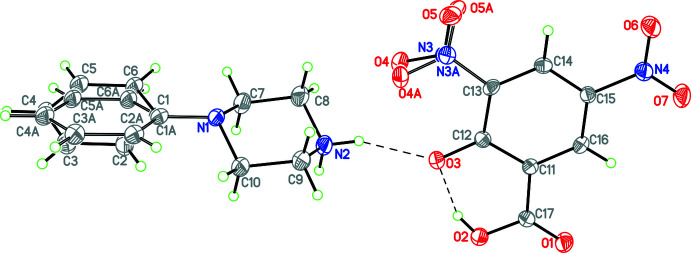
The mol­ecular structure of **6** with hydrogen bonds shown as dashed lines and disorder of the phenyl ring and one nitro group indicated. Atomic displacement parameters are at the 30% probability level.

For the structures containing the 3,5-di­nitro­salicylic (**6**), 3,5-di­nitro­benzoate (**7**) and 2,3,5-tri­nitro­phenolate ions (**8**), some inter­esting patterns emerge. In the anion of compound **6**, the carboxyl group is unionized, with C—O distances of 1.211 (4) and 1.309 (4) Å and it is the phenolic H atom that has been lost (Fig. 5 ▸). The C12—O3 distance, 1.283 (4) Å, is closer to that normally found in ketones than to that typical of phenols or phenolates (Allen *et al.*, 1987 ▸). In addition, the C11—C12 and C12—C13 distances, 1.428 (4) and 1.449 (5) Å, respectively, are significantly larger than the other C—C distances in this ring, which lie in the rather narrow range 1.370 (4)–1.398 (4) Å, but the C—N and N—O distances of the nitro substituents are all typical of their types. These observations indicate that the negative charge in this anion is delocalized over the five atoms C11, C13, C14, C15 and C16, but without any significant delocalization onto the nitro groups, as has been observed in tri­nitro­phenolate (picrate) anions (Kavitha *et al.*, 2006 ▸; Sagar *et al.*, 2017 ▸; Shaibah *et al.*, 2017*a* ▸,*b* ▸). The carboxyl­ate anion in **7** contains similar C—O distances [C17—O1 = 1.251 (14); C17—O2 = 1.256 (14) Å]. Structure **8** contains a picrate anion. Here the situation is similar to that of **6** in that the C—O distance is even shorter at 1.244 (3) Å and in the phenyl ring the C—C bonds are not equal with C11—C12 and C11—C16 being 1.443 (3) and 1.445 (3) Å, respectively, while the remaining C—C bonds range from 1.360 (3) to 1.386 (3) Å. For the nitro groups the C—N distances range from 1.441 (3) to 1.456 (3) Å, indicating that the negative charge in this anion is also delocalized over the five atoms C11, C13, C14, C15 and C16, but without any significant delocalization onto the nitro groups.

Structure **10** contains the tosyl­ate anion. There are two formula units in the asymmetric unit and in both anions the S—O distances are almost equal within experimental error ranging from 1.448 (12) to 1.462 (11) Å and 1.430 (13) to 1.473 (11) Å. Structures **11** and **12** contain the mono-anions of the di-carb­oxy­lic acids tartaric acid and fumaric acid. For both structures the metrical parameters of both cation and anion are in the normal range for such species. It notable that in **1** and **3**–**11**, the phenyl substituent occupies an equatorial position in the piperazinium cation, but for **12** this substituent occupies an axial position.

## 3. Supra­molecular features

In discussing the supra­molecular features of these eleven mol­ecular salts it is convenient to break these up into four groups based on the nature of the anion and the stoichiometry of the resulting salt. In the first group are structures **1**, **3**, **4**, **5**, and **9**, which contain benzoate and substituted benzoate anions. In the second group are **6**, **7**, and **8** in which the anions contain nitrated phenyl rings. In the third group, **10** contains a tosyl­ate anion, and in the fourth group, **11** and **12** contain the mono-deprotonated di­carboxyl­ate anions hydrogen tartarate and hydrogen fumarate. The hydrogen bonds for **1** and **3**–**12** are listed in Tables 1 ▸–11 ▸ ▸ ▸ ▸ ▸ ▸ ▸ ▸ ▸ ▸.

**Figure 12:**
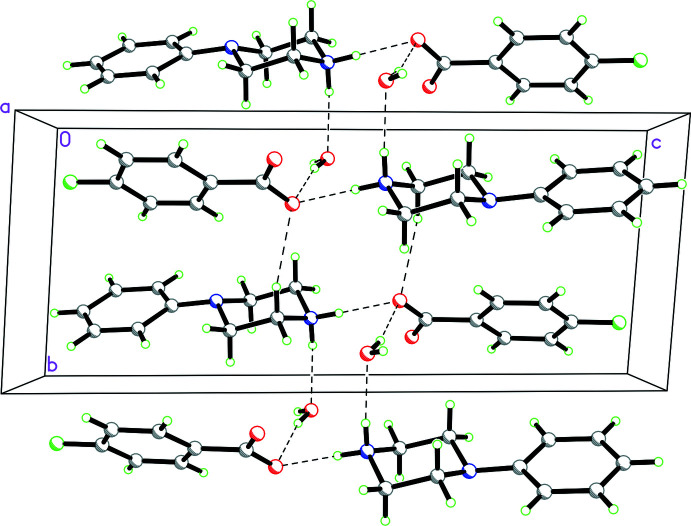
Packing diagram for **1** viewed along the *a* axis showing the 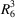 (12) rings made up of N—H⋯O and O—H⋯O hydrogen bonds, which involve the water mol­ecule, and 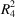 (10) rings made up of N—H⋯O hydrogen bonds, which do not involve the water mol­ecule. This combination of 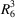 (12) and 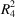 (10) rings form ribbons propagating in the *a*-axis direction.

**Figure 13:**
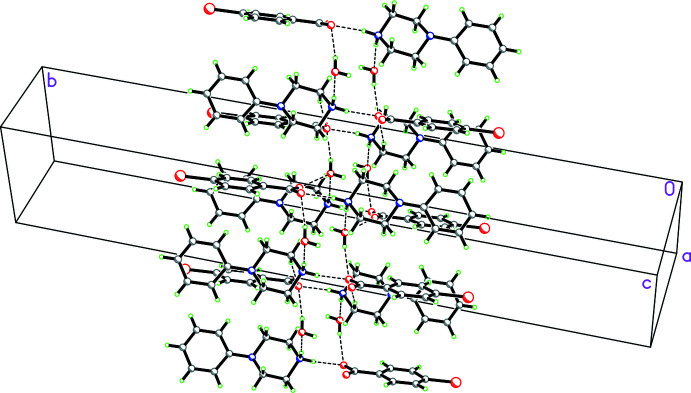
Packing diagram for **3** viewed along the [101] direction showing both 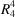 (12) rings demonstrating N—H⋯O and O—H⋯O hydrogen bonds involving the cations, anions and water mol­ecule as well as 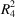 (10) rings showing O—H⋯O hydrogen bonds just associated with the cations and anions. These link the cations, anions and water mol­ecules into ribbons propagating in the [101] direction.

Even though **1**, **3**, **4**, **5**, and **9** contain similar anions, only **3** and **9** are isomorphous. For **1** (Fig. 12 ▸), which contains a water mol­ecule of crystallization, there are 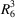 (12) rings (Etter *et al.*, 1990 ▸) made up of N—H⋯O and O—H⋯O hydrogen bonds, which involve the water mol­ecule, and 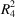 (10) rings made up of N—H⋯O hydrogen bonds, which do not involve the water mol­ecule. This combination of 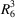 (12) and 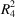 (10) rings form ribbons propagating in the *a*-axis direction. In addition, there is a C—H⋯π inter­action involving C2—H2 and the C11–C16 phenyl ring (*Cg*1) [C2⋯*Cg*1 = 3.610 (6) Å; C2—H2⋯*Cg*1 = 133°; symmetry operation −*x*, 1 − *y*, 1 − *z*]. In the packing arrangement for **3** shown in Fig. 13 ▸, both 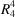 (12) rings exhibit N—H⋯O and O—H⋯O hydrogen bonds involving the cation, anion and water mol­ecule as well as 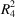 (10) rings showing O—H⋯O hydrogen bonds just associated with the cation and anion. These link the cations, anions and water mol­ecules into ribbons propagating in the [101] direction. In addition there is a C—H⋯π inter­action involving C2—H2 and the C11–C16 phenyl ring (*Cg*1) [C2⋯*Cg*1 = 3.6040 (14) Å; C2—H2⋯*Cg*1 = 133°; symmetry operation 1 − *x*, 1 − *y*, 1 − *z*].

**Figure 14:**
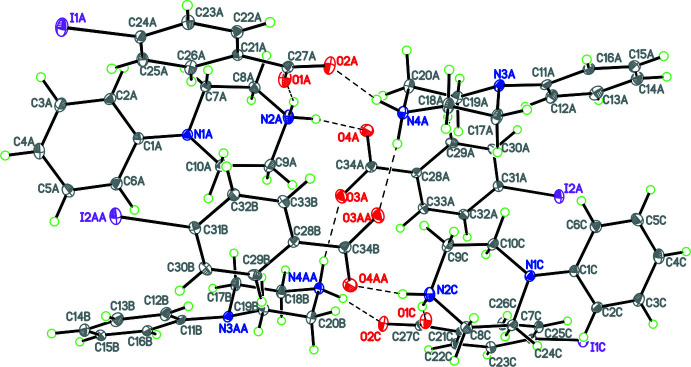
Partial packing diagram for **4** showing the 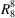 (24) ring with a topology analogous to the seam of a tennis ball involving N—H⋯O hydrogen bonds.

**Figure 15:**
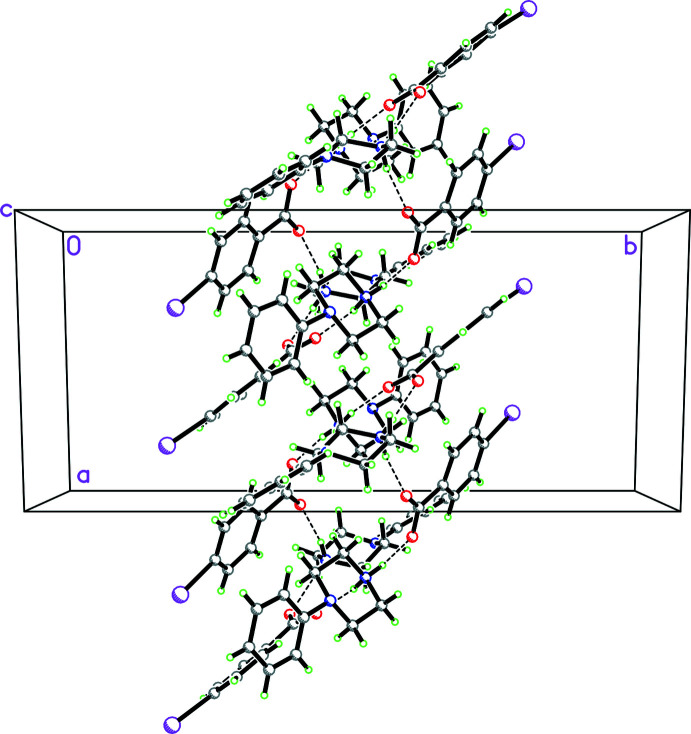
Packing diagram for **4** viewed along the *c* axis showing how the 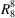 (24) rings pack in the *a*-axis direction.

**Figure 16:**
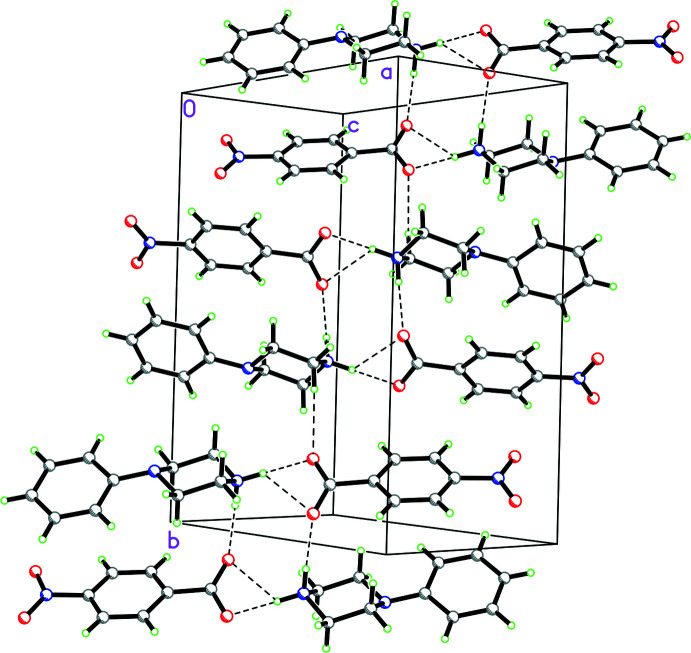
Packing diagram for **5** showing how the 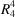 (12) rings link the cation and anion *via* N—H⋯O hydrogen bonds and this collection forms ribbons propagating in the *b*-axis direction.

In **4**, there is no water mol­ecule of crystallization. In this case there is an 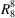 (24) ring with a topology analogous to the seam of a tennis ball (Fig. 14 ▸) involving N—H⋯O hydrogen bonds. These collections of cations and anions linked by 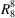 (24) rings pack in the *a*-axis direction (Fig. 15 ▸). In addition there is a C—H⋯π inter­action involving C19—H19 and the C21–C26 phenyl ring (*Cg*1) [C19⋯*Cg*1 = 3.750 (4) Å; C19—H19*B*⋯*Cg*1 = 154°; symmetry operation 1 − *x*, 1 − *y*, 1 − *z*]. In **5**, 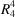 (12) rings link the cations and anions *via* N—H⋯O hydrogen bonds and this collection forms ribbons in the *b*-axis direction (Fig. 16 ▸). In addition, the –NO_2_ group accepts an N—H⋯(O,O) bifurcated hydrogen bond. There is a further inter­action with a phenyl ring (C11–C16, *Cg*1) involving the nitro substituent [N3⋯*Cg*1 = 3.530 (14) Å; N3—O3⋯*Cg*1 = 140.6 (13)°, symmetry operation *x*, 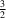 − *y*, 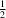 + *z*].

**Figure 17:**
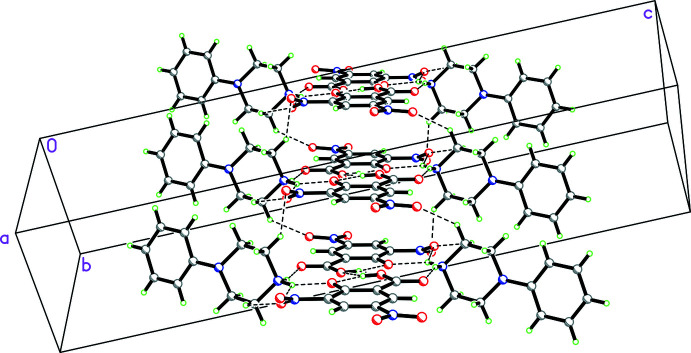
Packing diagram for **6** showing 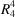 (16) loops linking the phenyl­piperazinium cations and the 3,5-dintrosalicylate anions *via* N—H⋯O and O—H⋯O hydrogen bonds.

In **6**, there are 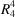 (16) loops linking the phenyl­piperazinium cations and the 3,5-dintrosalicylate anions *via* N—H⋯O hydrogen bonds (Fig. 17 ▸). In addition, there are π–π inter­actions involving the phenyl ring (C11–C16, *Cg*1) of the 3,5-dintrosalicylate anions, which form offset stacks (slippages of 1.580 and 1.900 Å) in the [110] direction [*Cg*1⋯*Cg*1 = 3.3600 (15) Å; symmetry operation −*x*, 1 − *y*, 1 − *z*; *Cg*1⋯*Cg*1 = 3.3690 (15) Å; symmetry operation −*x*, 2 − *y*, 1 − *z*].

**Figure 18:**
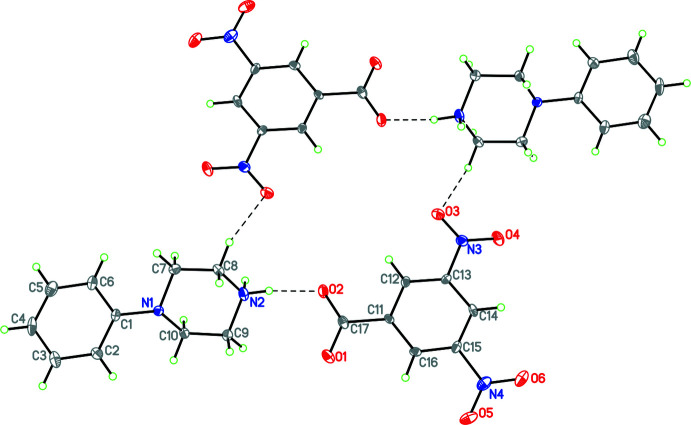
Partial packing diagram for **7** showing 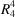 (22) rings in the (101) plane made up of N—H⋯O hydrogen-bonding inter­actions involving the phenyl­piperazinium cation and carboxyl­ate group of the 3,5-di­nitro­benzoate anion.

**Figure 19:**
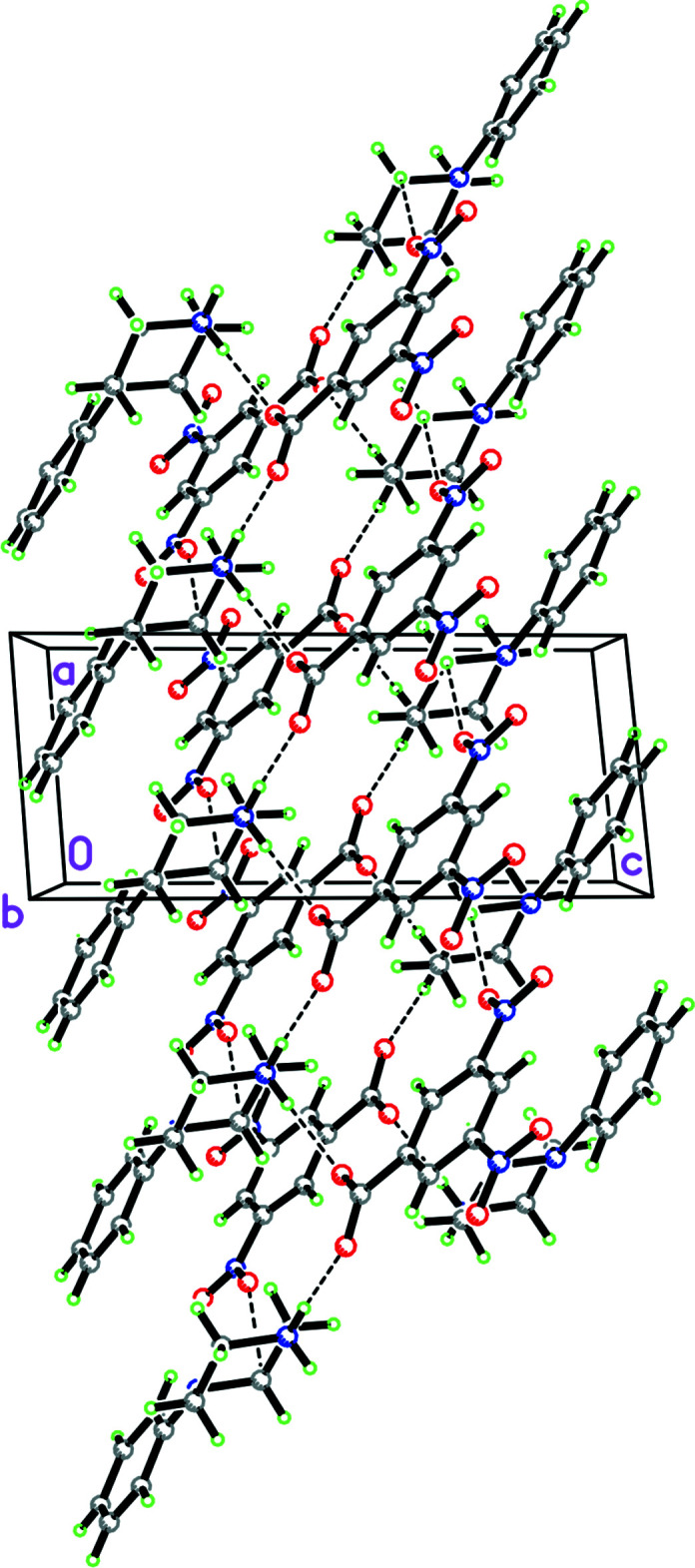
Packing diagram for **7** showing how the 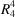 (22) rings shown in the previous figure are linked in the [111] direction by 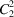 (6) chains also involving N—H⋯O hydrogen bonds involving the phenyl­piperazinium cation and carboxyl­ate group of the 3,5-di­nitro­benzoate anion and weak C—H⋯O inter­actions.

The packing of **7** is composed of 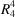 (22) rings in the (101) plane made up of N—H⋯O hydrogen bonds involving the phenyl­piperazinium cation and carboxyl­ate group of the 3,5-di­nitro­benzoate anion (Fig. 18 ▸). These planes are linked in the [111] direction by 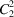 (6) chains also involving N—H⋯O hydrogen-bonding inter­actions involving the phenyl­piperazinium cation and carboxyl­ate group of the 3,5-di­nitro­benzoate anion and weak C—H⋯O inter­actions (Fig. 19 ▸). In this structure there are no C—H⋯π or π–π inter­actions.

**Figure 20:**
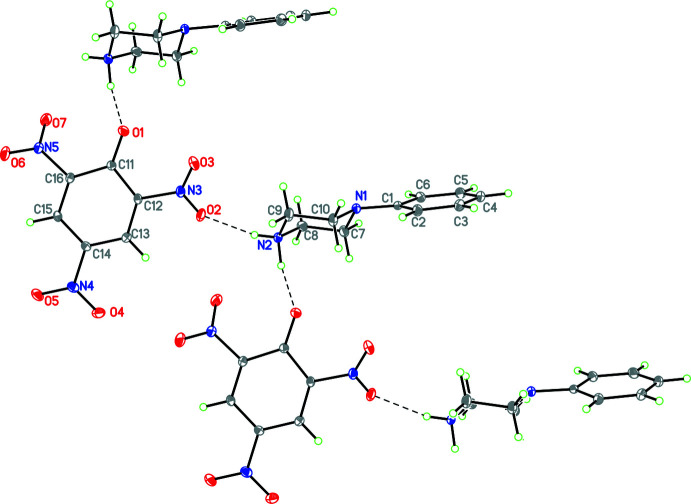
Partial packing diagram for **8** showing the 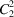 (8) chains made up of N—H⋯O hydrogen bonds involving the phenyl­piperazinium cation and a nitro group of the picrate anion. Hydrogen-bonding inter­actions are shown by dashed lines.

**Figure 21:**
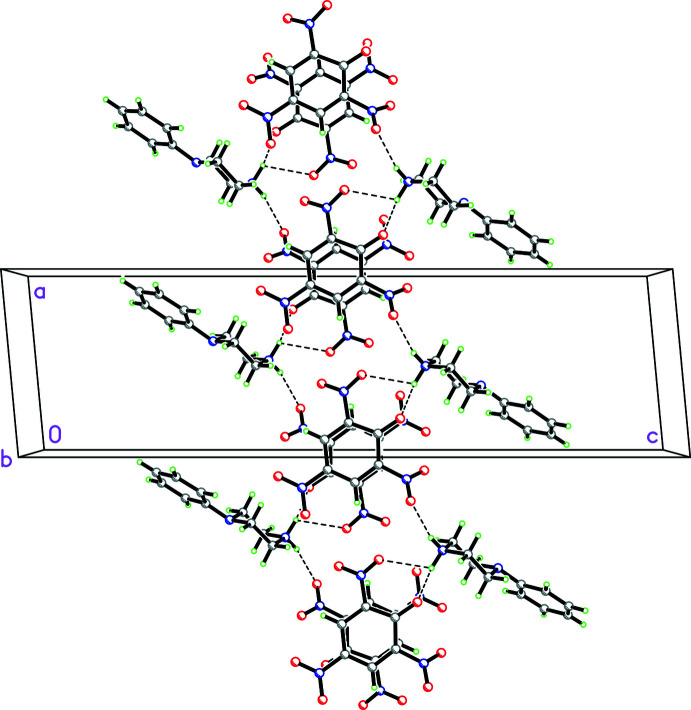
Packing diagram for **8** viewed along the *b* axis showing how the picrate anions form π–π inter­actions in the *a*-axis direction.

**Figure 22:**
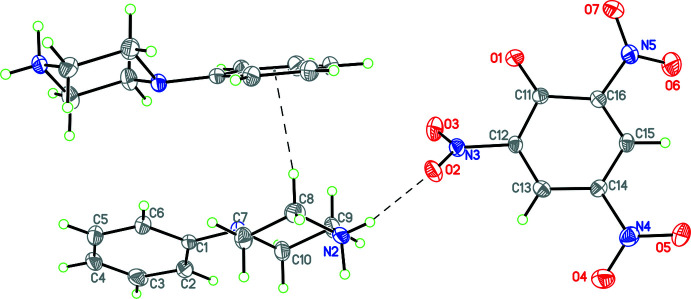
Partial packing diagram for **8** showing one of the C—H⋯π inter­actions involving the phenyl ring of the phenyl­piperazinium cation.

In **8** there are 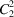 (8) chains made up of N—H⋯O hydrogen bonds involving the phenyl­piperazinium cation and a nitro group of the picrate anion (Fig. 20 ▸). In addition, the picrate anions form strong π–π inter­actions (C1–C6, *Cg*1) in the *a*-axis direction [*Cg*1⋯*Cg*1 = 3.4395 (5) Å; symmetry operation 2 − *x*, 1 − *y*, −*z*; *Cg*1⋯*Cg*1 = 3.4223 (5) Å; symmetry operation 2 − *x*, −*y*, −*z*] (Fig. 21 ▸). Furthermore, there are C—H⋯π inter­actions involving the phenyl ring (C1–C6, *Cg*1) of the phenyl­piperazinium cation [C3⋯*Cg*1 = 3.683 (3) Å, C3—H3⋯*Cg*1 = 134°, symmetry operation 2 − *x*, 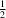 + *y*, 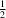 − *z*; C8⋯*Cg*1 = 3.512 (3) Å, C8—H8*A*⋯*Cg*1 = 160°, symmetry operation 1 − *x*, − 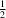 + *y*, 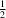 − *z*] (one example shown in Fig. 22 ▸).

**Figure 23:**
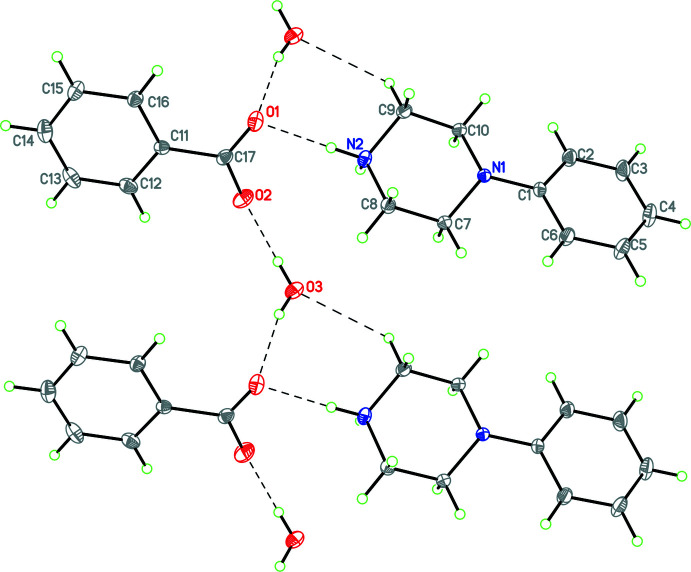
Partial packing diagram for **9** showing one of the two anti-parallel 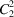 (6) chains linked by N—H⋯O hydrogen bonds and C—H⋯O inter­actions propagating in the *a-*axis direction.

**Figure 24:**
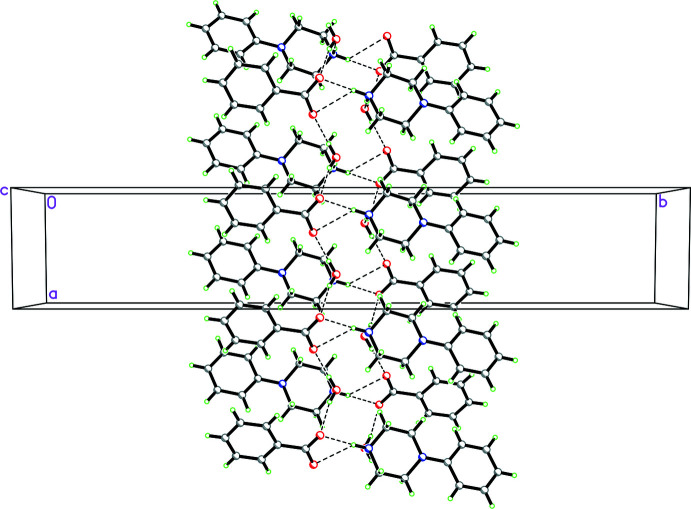
Packing diagram for **9** viewed along the *c-*axis direction showing the two anti-parallel 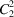 (6) chains linked by N—H⋯O and C—H⋯O inter­actions involving the water oxygen atom, which combine to form ribbons in the *a-*axis direction.

In the case of **9** there are two anti-parallel 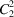 (6) chains linked by N—H⋯O hydrogen bonds as well as C—H⋯O inter­actions involving the water oxygen atom, which combine to form ribbons propagating in the *a*-axis direction (Fig. 23 ▸). In addition, there are C—H⋯π inter­actions (C11–C16, *Cg*1) involving the benzoate phenyl ring [C2⋯*Cg*1 = 3.710 (4) Å, C2—H2⋯*Cg*1 = 141°, symmetry operation −*x*, 1 − *y*, 1 − *z*; C6⋯*Cg*1 = 3.656 (4) Å, C6—H6⋯*Cg*1 = 142°, symmetry operation 1 − *x*, 1 − *y*, 2 − *z*]. The overall packing is shown in Fig. 24 ▸.

**Figure 25:**
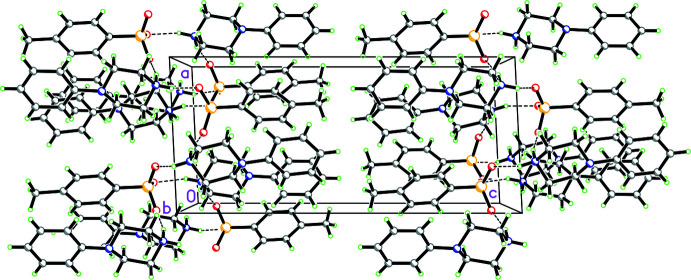
Packing diagram for **10** viewed down the *b* axis showing the three-dimensional network of N—H⋯O hydrogen bonds involving phenyl­piperazinium cations and tosyl­ate anions, which arrange the ions such that there are hydro­philic and hydro­phobic (110) planes in the *a*-axis direction.

The structure of **10** contains the tosyl­ate anion, which contains the non-planar –SO_3_^−^ group. This results in a packing arrangement in which N—H⋯O hydrogen bonds involving the phenyl­piperazinium cations and tosyl­ate anions are arranged such that there are hydro­philic and hydro­phobic (110) planes (Fig. 25 ▸). This structure also contains C—H⋯π inter­actions involving one of the phenyl­piperazinium cations (C18–C23, *Cg*1) and tosyl­ate anions [C30⋯*Cg*1 = 3.74 (3) Å, C30—H30⋯*Cg*1 = 144°, symmetry operation 1 − *x*, − 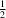 + *y*, −*z*].

**Figure 26:**
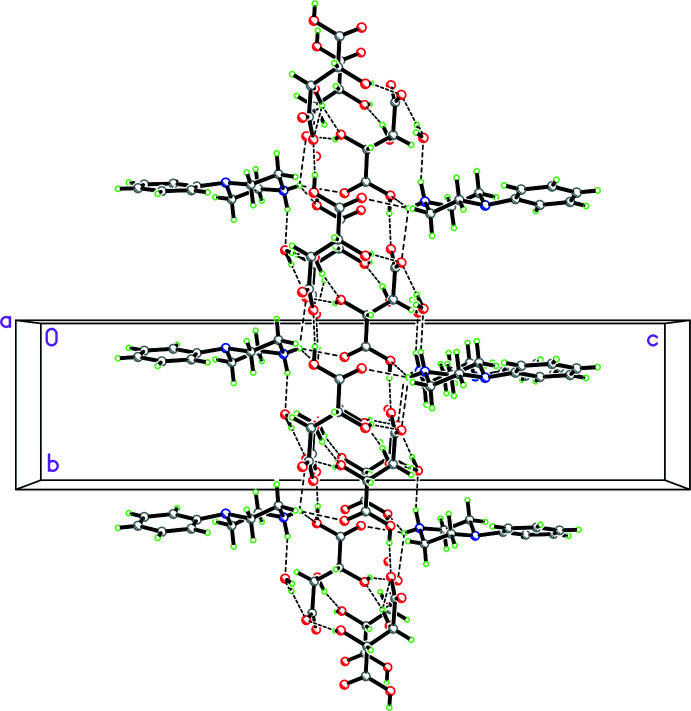
Packing diagram for **11** viewed along the *a* axis where multiple N—H⋯O and O—H⋯O hydrogen bonds involving the phenyl­piperazinium NH_2_ group, the tartarate anion and water mol­ecule of crystallization combine to form a three-dimensional network.

Structure **11** has a complicated packing arrangement as in addition to the phenyl­piperazinium NH_2_ group, the flexible tartarate anion contains four OH groups and there is a water mol­ecule of crystallization. Multiple N—H⋯O and O—H⋯O hydrogen-bonding inter­actions combine to form a three-dimensional array (Fig. 26 ▸).

**Figure 27:**
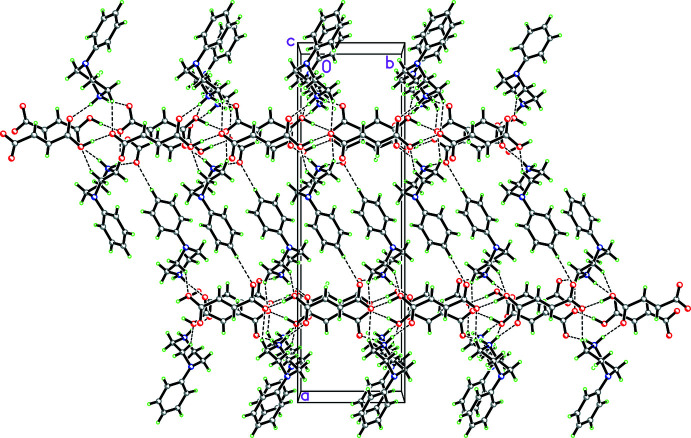
Packing diagram for **12** viewed along the *c-*axis direction showing the two 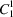 (7) chains propagating in the *b*-axis direction involving the fumarate anions and composed of O—H⋯O hydrogen bonds which are in turn cross-linked by both N—H⋯O hydrogen bonds and C—H⋯O inter­actions.

Structure **12** contains a phenyl­piperazinium cation and the monoanion of fumaric acid. In the packing of this structure, there are two 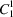 (7) chains in the *b*-axis direction involving the fumarate anions and composed of O—H⋯O hydrogen bonds. These chains are in turn cross-linked by both N—H⋯O hydrogen bonds and C—H⋯O inter­actions (Fig. 27 ▸). There are also C—H⋯π inter­actions involving the phenyl ring (C1–C6, *Cg*1) of the phenyl­piperazinium cation [C5⋯*Cg*1 = 3.723 (3) Å, C5—H5⋯*Cg*1 =144°, symmetry operation −*x*, 1 − *y*, 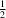 + *z*; C10⋯*Cg*1 = 3.608 (3) Å, C10—H10*A*⋯*Cg*1 = 145°, symmetry operation −*x*, −*y*, − 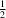 + *z*].

The Hirshfeld surface fingerprint plots for **1** and **3**–**12** generated using *CrystalExplorer* are available in the supporting information. All of them show the distinctive ‘pincer spikes’ associated with the N—H⋯O and/or O—H⋯O hydrogen bonds (Spackman *et al.*, 2021 ▸).

## 4. Database survey

The structural versatility of the 1-phenyl­piperazine moiety itself is shown by its involvement in many structural forms, including as neutral co-crystals [Cambridge Structural Database (Groom *et al.*, 2016 ▸) refcodes HINQUR and HINRAY, Müller-Buschbaum & Zurawski, 2007 ▸], as neutral ligands (HIWJAY, Stocker *et al.*, 1999 ▸; HIWJAY01, VIYPIE, VIYPOK, VIYPUQ; Pike *et al.*, 2014 ▸), as simultaneously both neutral ligands and co-crystals (FITTEI and FITTIM, Quitmann & Müller-Buschbaum, 2005 ▸; HOCBEH, HOCBIL, PIYXEB, Zurawski & Müller-Buschbaum, 2008 ▸). In addition, there have been many structural investigations of 1-phenyl­piperazine as a cation, combined with simple anions (DMPIPZ, Cho­thia & Pauling, 1978 ▸; JEHXIE, Batsanov *et al.*, 2006 ▸; KUZWUY, Marouani *et al.*, 2010 ▸; LOHQIL, Oueslati *et al.*, 2019 ▸; QORVEB, Marouani *et al.*, 2012 ▸; SUYXEQ, Essid *et al.*, 2010 ▸), with simple anionic metal salts (BEBKAX, Lahbib *et al.*, 2017 ▸; CEBHIB, Garbia *et al.*, 2005 ▸; PENWAJ, Mathlouthi *et al.*, 2017 ▸; PHPIPZ, Battaglia *et al.*, 1979 ▸; QIZPIA, Dhieb *et al.*, 2014 ▸; SUKKAM, Dhieb *et al.*, 2015 ▸; ZAMHUQ, Zouari *et al.*, 1995 ▸), combined with anionic carboxyl­ates (IGOGUI, Pang *et al.*, 2015 ▸; VAKCIW, Zong *et al.*, 2016 ▸; Mahesha *et al.*, 2022 ▸), combined with anionic pyrimidines (DUPMUY, DUPNAF, Al-Alshaikh *et al.*, 2015 ▸), combined with anionic ligands (WOVKAW, Lo *et al.*, 2019 ▸), combined with a clathrate (GUBHOB, Wu *et al.*, 2009 ▸), and combined with anionic metal complexes (DUJPIK, Shin *et al.*, 2020 ▸; SICGUJ, Nasr *et al.*, 2018 ▸; SICGUJ01, Khedhiri *et al.* 2018 ▸).

## 5. Synthesis and crystallization

For the synthesis of salts **1**–**12**, a solution of commercially available 1-phenyl­piperazine (100 mg, 0.62 mol) (from Sigma-Aldrich) in methanol (10 ml) was mixed with an equimolar solution of (**1**) 4-fluoro­benzoic acid (87 mg, 0.62 mol), (**2**) 4-chloro­benzoic acid (97 mg, 0.62 mol), (**3**) 4-bromo­benzoic acid (125 mg, 0.62 mol), (**4**) 4-iodo­benzoic acid (154 mg, 0.62 mol), (**5**) 4-nitro­benzoic acid (104 mg, 0.62 mol), (**6**) 3,5-di­nitro­salicylic acid (104 mg, 0.62 mol), (**7**) 3,5-di­nitro­benzoic acid (132 mg, 0.62 mol), (**8**) picric acid (142 mg, 0.62 mol), (**9**) benzoic acid (76 mg, 0.62 mol), (**10**) *p*-toluene­sulfonic acid (107 mg, 0.62 mol), (**11**) tartaric acid (93 mg, 0.62 mol) and (**12**) fumaric acid (72 mg, 0.62 mol). The resulting mixture was stirred for 30 min at 323 K and allowed to stand at room temperature. X-ray quality crystals of **1** and **3**–**12** were formed on slow evaporation after one week (m.p.: 381–384 K (**1**), 382–387 K (**3**), 413–418 K (**4**), 423–428 K (**5**), 431–436 K (**6**), 427–429 K (**7**), 430–433 K (**8**), 455–457 K (**9**), 377–380 K (**10**), 416–420 K (**11**) and 438–440 K (**12**). No crystals of (**2)** (m.p. 488–490 K) suitable for X-ray diffraction were obtained.

## 6. Refinement

Crystal data, data collection and structure refinement details for structures **1** and **3**–**12** are summarized in Table 12 ▸. All hydrogen atoms were positioned geometrically with their *U*_iso_ values 1.2 times that of their attached atoms. For some structures (**6**, **10**, and **11**), the phenyl ring of the piperazinium cation was disordered over two orientations in ratios of 0.687 (10)/0.313 (10); 0.51 (7)/0.49 (7), and 0.611 (13)/0.389 (13) for **6**, **10**, and **11**, respectively. For both **5** and **6**, a nitro group was disordered and modeled with two orientations with occupancies of 0.62 (3)/0.38 (3) and 0.690 (11)/0.310 (11), respectively.

## Supplementary Material

Crystal structure: contains datablock(s) 1, 3, 4, 5, 6, 7, 8, 9, 10, 11, 12. DOI: 10.1107/S2056989022009057/hb8034sup1.cif

Structure factors: contains datablock(s) 1. DOI: 10.1107/S2056989022009057/hb80341sup2.hkl

Supporting information file. DOI: 10.1107/S2056989022009057/hb80341sup22.cml

Supporting information file. DOI: 10.1107/S2056989022009057/hb80343sup12.cml

Structure factors: contains datablock(s) 3. DOI: 10.1107/S2056989022009057/hb80343sup3.hkl

Supporting information file. DOI: 10.1107/S2056989022009057/hb80344sup13.cml

Structure factors: contains datablock(s) 4. DOI: 10.1107/S2056989022009057/hb80344sup4.hkl

Supporting information file. DOI: 10.1107/S2056989022009057/hb80345sup14.cml

Structure factors: contains datablock(s) 5. DOI: 10.1107/S2056989022009057/hb80345sup5.hkl

Supporting information file. DOI: 10.1107/S2056989022009057/hb80346sup15.cml

Structure factors: contains datablock(s) 6. DOI: 10.1107/S2056989022009057/hb80346sup6.hkl

Supporting information file. DOI: 10.1107/S2056989022009057/hb80347sup16.cml

Structure factors: contains datablock(s) 7. DOI: 10.1107/S2056989022009057/hb80347sup7.hkl

Supporting information file. DOI: 10.1107/S2056989022009057/hb80348sup17.cml

Structure factors: contains datablock(s) 8. DOI: 10.1107/S2056989022009057/hb80348sup8.hkl

Supporting information file. DOI: 10.1107/S2056989022009057/hb80349sup18.cml

Structure factors: contains datablock(s) 9. DOI: 10.1107/S2056989022009057/hb80349sup9.hkl

Structure factors: contains datablock(s) 10. DOI: 10.1107/S2056989022009057/hb803410sup10.hkl

Supporting information file. DOI: 10.1107/S2056989022009057/hb803410sup19.cml

Structure factors: contains datablock(s) 11. DOI: 10.1107/S2056989022009057/hb803411sup11.hkl

Supporting information file. DOI: 10.1107/S2056989022009057/hb803411sup20.cml

Structure factors: contains datablock(s) 12. DOI: 10.1107/S2056989022009057/hb803412sup12.hkl

Supporting information file. DOI: 10.1107/S2056989022009057/hb803412sup21.cml

Hirschfeld fingerprint plots for the eleven structures. DOI: 10.1107/S2056989022009057/hb8034sup22.docx

CCDC references: 2206380, 2206379, 2206378, 2206377, 2206376, 2206375, 2206374, 2206373, 2206372, 2206371, 2206370

Additional supporting information:  crystallographic information; 3D view; checkCIF report

## Figures and Tables

**Figure 2:**
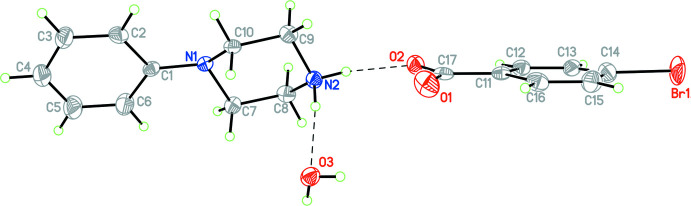
The mol­ecular structure of **3** with hydrogen bonds shown as dashed lines. Atomic displacement parameters are at the 30% probability level.

**Figure 3:**
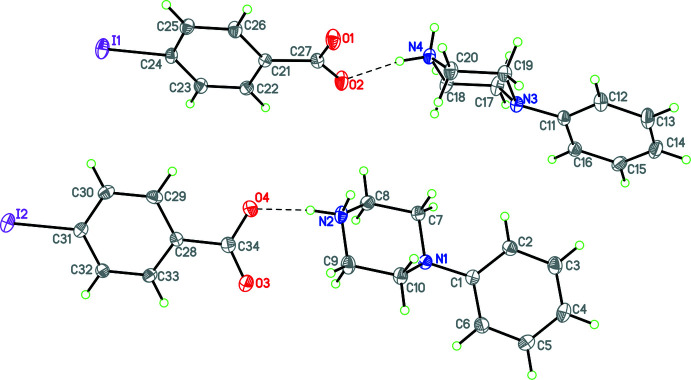
The mol­ecular structure of **4** with hydrogen bonds shown as dashed lines. Atomic displacement parameters are at the 30% probability level.

**Figure 4:**
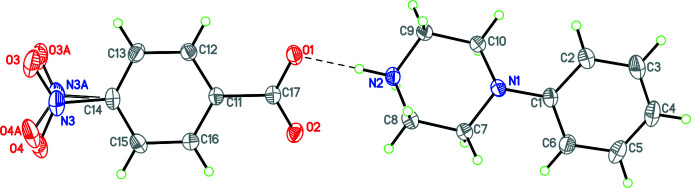
The mol­ecular structure of **5** with hydrogen bonds shown as dashed lines and disorder of the nitro group indicated. Atomic displacement parameters are at the 30% probability level.

**Figure 6:**
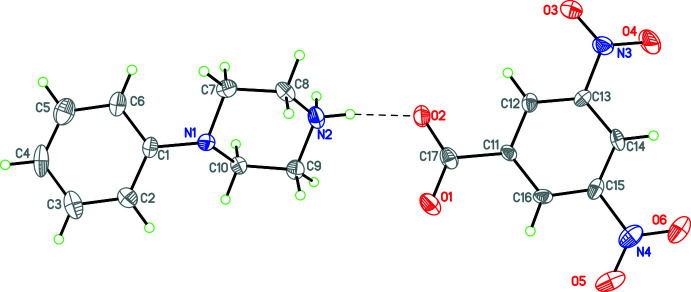
The mol­ecular structure of **7** with hydrogen bonds shown as dashed lines. Atomic displacement parameters are at the 30% probability level.

**Figure 7:**
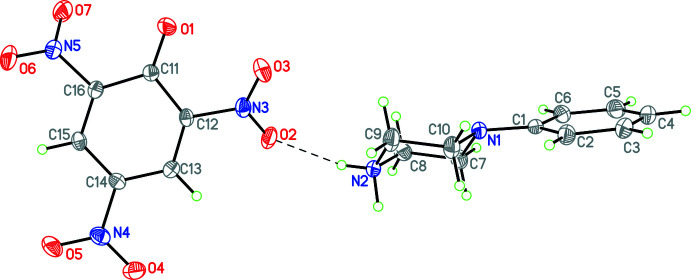
The mol­ecular structure of **8** with hydrogen bonds shown as dashed lines. Atomic displacement parameters are at the 30% probability level.

**Figure 8:**
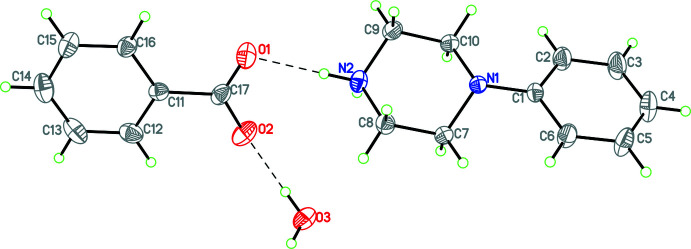
The mol­ecular structure of **9** with hydrogen bonds shown as dashed lines. Atomic displacement parameters are at the 30% probability level.

**Figure 9:**
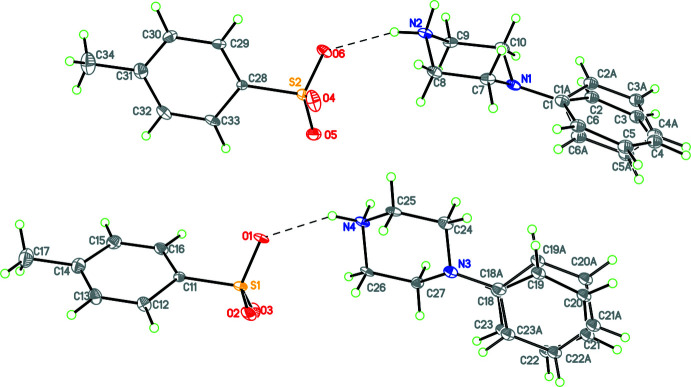
The mol­ecular structure of **10** with hydrogen bonds shown as dashed lines and disorder of the phenyl rings indicated. Atomic displacement parameters are at the 30% probability level.

**Figure 10:**
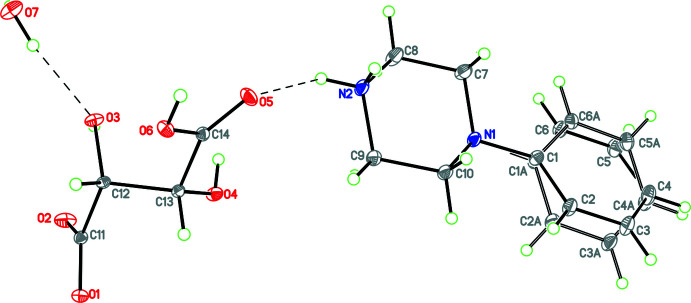
The mol­ecular structure of **11** with hydrogen bonds shown as dashed lines and disorder of the phenyl ring indicated. Atomic displacement parameters are at the 30% probability level.

**Figure 11:**
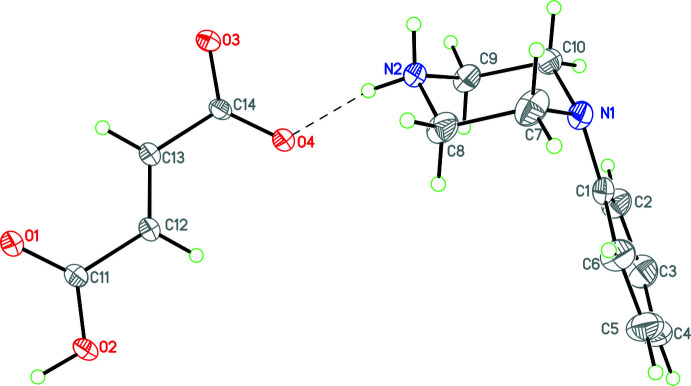
The mol­ecular structure of **12** with hydrogen bonds shown as dashed lines. Atomic displacement parameters are at the 30% probability level. Note the axial conformation of the phenyl ring.

**Table 1:** Hydrogen-bond geometry (Å, °) for **1** [Chem scheme1]

*D*—H⋯*A*	*D*—H	H⋯*A*	*D*⋯*A*	*D*—H⋯*A*
N2—H21⋯O1	0.90 (2)	2.65 (4)	3.215 (6)	122 (3)
N2—H21⋯O2	0.90 (2)	1.89 (2)	2.791 (5)	175 (4)
N2—H22⋯O3^i^	0.88 (2)	1.96 (2)	2.812 (5)	163 (4)
C8—H8*A*⋯O2^ii^	0.97	2.53	3.481 (6)	168
C8—H8*B*⋯O3	0.97	2.60	3.341 (5)	133
C9—H9*A*⋯O3^iii^	0.97	2.59	3.416 (6)	143
O3—H31⋯O1^iv^	0.83 (2)	1.79 (2)	2.619 (5)	176 (6)
O3—H32⋯O2	0.83 (2)	1.96 (2)	2.773 (5)	167 (6)

Symmetry codes: (i) 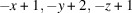 ; (ii) 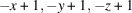 ; (iii) 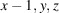 ; (iv) 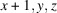 .

**Table 2:** Hydrogen-bond geometry (Å, °) for **3** [Chem scheme1]

*D*—H⋯*A*	*D*—H	H⋯*A*	*D*⋯*A*	*D*—H⋯*A*
N2—H2*N*1⋯O2	0.89	1.90	2.780 (11)	170
N2—H2*N*2⋯O3	0.89	1.94	2.803 (12)	164
C8—H8*A*⋯O3^i^	0.97	2.64	3.377 (14)	133
C8—H8*B*⋯O2^ii^	0.97	2.53	3.475 (14)	166
C9—H9*B*⋯O3^iii^	0.97	2.59	3.403 (14)	142
O2—H2*O*⋯N2	0.82	2.00	2.780 (11)	159
O3—H31⋯O2^i^	0.81 (2)	1.98 (2)	2.782 (12)	170 (7)

Symmetry codes: (i) 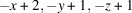 ; (ii) 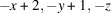 ; (iii) 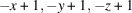 .

**Table 3:** Hydrogen-bond geometry (Å, °) for **4** [Chem scheme1]

*D*—H⋯*A*	*D*—H	H⋯*A*	*D*⋯*A*	*D*—H⋯*A*
C9—H9*A*⋯O3	0.97	2.63	3.218 (4)	119
N2—H21*N*⋯O1^i^	0.88 (2)	1.94 (2)	2.780 (4)	160 (3)
N2—H22*N*⋯O4	0.90 (2)	1.74 (2)	2.627 (4)	173 (3)
N4—H41*N*⋯O1	0.87 (2)	2.63 (3)	3.285 (4)	133 (3)
N4—H41*N*⋯O2	0.87 (2)	1.78 (2)	2.643 (4)	169 (3)
N4—H42*N*⋯O3^ii^	0.85 (2)	1.97 (2)	2.809 (4)	169 (4)

Symmetry codes: (i) 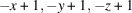 ; (ii) 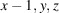 .

**Table 4:** Hydrogen-bond geometry (Å, °) for **5** [Chem scheme1]

*D*—H⋯*A*	*D*—H	H⋯*A*	*D*⋯*A*	*D*—H⋯*A*
N2—H21⋯O2^i^	0.90 (1)	1.96 (2)	2.846 (2)	173 (2)
N2—H22⋯O1	0.93 (1)	1.78 (2)	2.7135 (19)	179 (2)
N2—H22⋯O2	0.93 (1)	2.49 (2)	3.057 (2)	120 (1)
C8—H8*B*⋯O1^ii^	0.97	2.50	3.468 (2)	176
C10—H10*B*⋯O4*A* ^iii^	0.97	2.61	3.276 (15)	126

Symmetry codes: (i) 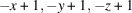 ; (ii) 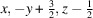 ; (iii) 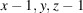 .

**Table 5:** Hydrogen-bond geometry (Å, °) for **6** [Chem scheme1]

*D*—H⋯*A*	*D*—H	H⋯*A*	*D*⋯*A*	*D*—H⋯*A*
O2—H2*O*⋯O3	0.84 (2)	1.69 (2)	2.487 (3)	156 (4)
N2—H21⋯O3	0.88 (2)	2.03 (2)	2.873 (4)	159 (3)
N2—H21⋯O4	0.88 (2)	2.37 (3)	2.950 (6)	123 (3)
N2—H21⋯O4*A*	0.88 (2)	2.40 (4)	2.966 (10)	122 (3)
N2—H22⋯O1^i^	0.87 (2)	2.10 (2)	2.947 (4)	164 (4)
N2—H22⋯O2^i^	0.87 (2)	2.62 (3)	3.270 (4)	132 (3)
C8—H8*A*⋯O7^ii^	0.97	2.36	3.134 (5)	137
C8—H8*B*⋯O4	0.97	2.44	3.000 (6)	116
C9—H9*A*⋯O4*A*	0.97	2.60	3.166 (8)	118
C9—H9*B*⋯O5^iii^	0.97	2.58	3.311 (8)	132
C9—H9*B*⋯O5*A* ^iii^	0.97	2.29	3.040 (13)	133

Symmetry codes: (i) 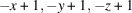 ; (ii) 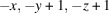 ; (iii) 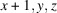 .

**Table 6:** Hydrogen-bond geometry (Å, °) for **7** [Chem scheme1]

*D*—H⋯*A*	*D*—H	H⋯*A*	*D*⋯*A*	*D*—H⋯*A*
C8—H8*A*⋯O3^i^	0.97	2.43	3.250 (14)	142
C10—H10*B*⋯O5^ii^	0.97	2.58	3.366 (16)	138
N2—H21⋯O2	0.87 (3)	1.81 (4)	2.672 (13)	172 (13)
N2—H22⋯O1^iii^	0.87 (3)	1.94 (4)	2.792 (13)	166 (12)

Symmetry codes: (i) 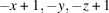 ; (ii) 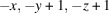 ; (iii) 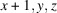 .

**Table 7:** Hydrogen-bond geometry (Å, °) for **8** [Chem scheme1]

*D*—H⋯*A*	*D*—H	H⋯*A*	*D*⋯*A*	*D*—H⋯*A*
C8—H8*B*⋯O4^i^	0.97	2.42	3.265 (4)	145
C9—H9*A*⋯O4^ii^	0.97	2.60	3.353 (4)	134
C9—H9*A*⋯O6^iii^	0.97	2.61	3.455 (4)	146
N2—H21⋯O2	0.83 (3)	2.06 (3)	2.871 (3)	166 (3)
N2—H21⋯O7^iv^	0.83 (3)	2.60 (3)	2.985 (3)	110 (2)
N2—H22⋯O1^iv^	0.98 (3)	1.74 (3)	2.705 (3)	168 (3)

Symmetry codes: (i) 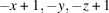 ; (ii) 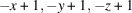 ; (iii) 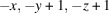 ; (iv) 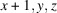 .

**Table 8:** Hydrogen-bond geometry (Å, °) for **9** [Chem scheme1]

*D*—H⋯*A*	*D*—H	H⋯*A*	*D*⋯*A*	*D*—H⋯*A*
N2—H21⋯O1	0.90 (2)	1.92 (2)	2.813 (3)	173 (2)
N2—H21⋯O2	0.90 (2)	2.56 (2)	3.112 (4)	121 (2)
N2—H22⋯O3^i^	0.89 (2)	1.92 (2)	2.812 (3)	173 (2)
C9—H9*A*⋯O1^ii^	0.97	2.48	3.420 (4)	164
C9—H9*B*⋯O3^iii^	0.97	2.60	3.340 (4)	133
O3—H31⋯O1^iv^	0.83 (2)	1.96 (2)	2.772 (3)	166 (4)
O3—H32⋯O2	0.83 (2)	1.77 (2)	2.599 (3)	179 (4)

Symmetry codes: (i) 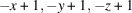 ; (ii) 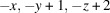 ; (iii) 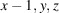 ; (iv) 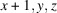 .

**Table 9:** Hydrogen-bond geometry (Å, °) for **10** [Chem scheme1]

*D*—H⋯*A*	*D*—H	H⋯*A*	*D*⋯*A*	*D*—H⋯*A*
N2—H21*N*⋯O6	0.89	2.07	2.884 (18)	151
N2—H22*N*⋯O4^i^	0.89	1.92	2.774 (17)	161
C9—H9*B*⋯O1^ii^	0.97	2.64	3.534 (19)	154
N4—H41*N*⋯O3^ii^	0.89	1.92	2.788 (16)	163
N4—H42*N*⋯O1	0.89	2.09	2.890 (18)	149
N4—H42*N*⋯O5	0.89	2.43	2.865 (18)	111
C25—H25*A*⋯O6^iii^	0.97	2.63	3.520 (18)	153

Symmetry codes: (i) 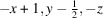 ; (ii) 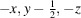 ; (iii) 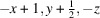 .

**Table 10:** Hydrogen-bond geometry (Å, °) for **11** [Chem scheme1]

*D*—H⋯*A*	*D*—H	H⋯*A*	*D*⋯*A*	*D*—H⋯*A*
N2—H21⋯O7^i^	0.88 (2)	1.95 (2)	2.808 (5)	164 (4)
N2—H22⋯O1^ii^	0.86 (2)	2.52 (3)	3.069 (4)	122 (3)
N2—H22⋯O5	0.86 (2)	2.22 (3)	2.820 (3)	127 (3)
N2—H22⋯O6^iii^	0.86 (2)	2.41 (3)	2.992 (3)	125 (3)
C9—H9*B*⋯O2^iv^	0.97	2.61	3.276 (4)	126
O3—H3*O*⋯O2	0.83 (2)	2.17 (3)	2.614 (3)	114 (3)
O3—H3*O*⋯O4^ii^	0.83 (2)	2.04 (2)	2.789 (3)	150 (3)
O4—H4*O*⋯O1^ii^	0.79 (2)	2.06 (3)	2.773 (3)	151 (3)
O6—H6*O*⋯O2^v^	0.83 (2)	1.67 (2)	2.501 (3)	174 (3)
O7—H71*O*⋯O3	0.84 (2)	1.95 (2)	2.780 (3)	171 (4)
O7—H72*O*⋯O1^vi^	0.85 (2)	1.97 (2)	2.821 (3)	178 (4)

Symmetry codes: (i) 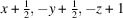 ; (ii) 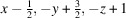 ; (iii) 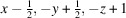 ; (iv) 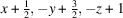 ; (v) 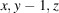 ; (vi) 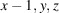 .

**Table 11:** Hydrogen-bond geometry (Å, °) for **12** [Chem scheme1]

*D*—H⋯*A*	*D*—H	H⋯*A*	*D*⋯*A*	*D*—H⋯*A*
N2—H21⋯O4	0.87 (2)	1.88 (2)	2.741 (2)	168 (2)
N2—H22⋯O1^i^	0.89 (2)	1.89 (2)	2.775 (2)	172 (2)
C7—H7*A*⋯O2^ii^	0.97	2.51	3.317 (3)	141
C8—H8*B*⋯O3^iii^	0.97	2.55	3.203 (3)	124
C9—H9*A*⋯O2^iv^	0.97	2.66	3.318 (3)	126
O2—H2*O*⋯O3^v^	0.92 (2)	1.54 (2)	2.4610 (18)	174 (2)

Symmetry codes: (i) 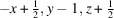 ; (ii) 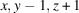 ; (iii) 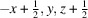 ; (iv) 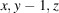 ; (v) 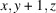 .

**Table:** 

	**1**	**3**	**4**	**5**
Crystal data				
Chemical formula	C_10_H_15_N_2_^+^·C_7_H_4_FO_2_^−^·H_2_O	C_10_H_15_N_2_^+^·C_7_H_4_BrO_2_^−^·H_2_O	C_10_H_15_N_2_^+^·C_7_H_4_IO_2_^−^	C_10_H_15_N_2_^+^·C_7_H_4_NO_4_^−^
*M*_r_	320.36	381.27	410.24	329.35
Crystal system, space group	Triclinic, *P* 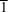	Monoclinic, *P*2_1_/*c*	Monoclinic, *P*2_1_/*c*	Monoclinic, *P*2_1_/*c*
Temperature (K)	293	293	293	293
*a*, *b*, *c* (Å)	6.239 (1), 7.496 (1), 17.817 (3)	6.183 (2), 37.748 (7), 7.506 (2)	10.8507 (4), 23.4045 (7), 13.3019 (4)	13.0683 (9), 15.7868 (9), 7.9255 (5)
α, β, γ (°)	93.55 (2), 92.94 (2), 94.87 (2)	90, 93.69 (4), 90	90, 102.491 (4), 90	90, 95.137 (6), 90
*V* (Å^3^)	827.3 (2)	1748.2 (8)	3298.13 (19)	1628.52 (18)
*Z*	2	4	8	4
Radiation type	Mo *K*α	Mo *K*α	Mo *K*α	Mo *K*α
μ (mm^−1^)	0.10	2.37	1.95	0.10
Crystal size (mm)	0.44 × 0.32 × 0.16	0.46 × 0.20 × 0.12	0.48 × 0.48 × 0.40	0.48 × 0.44 × 0.16

Data collection				
Diffractometer	Oxford Diffraction Xcalibur with Sapphire CCD	Oxford Diffraction Xcalibur with Sapphire CCD	Oxford Diffraction Xcalibur with Sapphire CCD	Oxford Diffraction Xcalibur with Sapphire CCD
Absorption correction	Multi-scan (*CrysAlis RED*; Oxford Diffraction, 2007 ▸)	Multi-scan (*CrysAlis RED*; Oxford Diffraction, 2007 ▸)	Multi-scan (*CrysAlis RED*; Oxford Diffraction, 2007 ▸)	Multi-scan (*CrysAlis RED*; Oxford Diffraction, 2007 ▸)
*T*_min_, *T*_max_	0.613, 1.000	0.613, 1.000	0.575, 1.000	0.790, 1.000
No. of measured, independent and observed [*I* > 2σ(*I*)] reflections	4669, 3013, 1611	6103, 3170, 1374	14154, 7079, 4641	11699, 3587, 2088
*R*_int_	0.033	0.061	0.024	0.035
(sin θ/λ)_max_ (Å^−1^)	0.602	0.602	0.657	0.660

Refinement				
*R*[*F* ^2^ > 2σ(*F* ^2^)], *wR*(*F* ^2^), *S*	0.090, 0.226, 1.10	0.138, 0.375, 1.03	0.037, 0.084, 1.02	0.054, 0.117, 1.10
No. of reflections	3013	3170	7079	3587
No. of parameters	220	215	409	251
No. of restraints	4	7	4	83
H-atom treatment	H atoms treated by a mixture of independent and constrained refinement	H atoms treated by a mixture of independent and constrained refinement	H atoms treated by a mixture of independent and constrained refinement	H atoms treated by a mixture of independent and constrained refinement
Δρ_max_, Δρ_min_ (e Å^−3^)	0.27, −0.20	0.49, −0.61	0.77, −1.25	0.15, −0.13
Absolute structure	–	–	–	–
Absolute structure parameter	–	–	–	–

**Table:** 

	**6**	**7**	**8**	**9**
Crystal data				
Chemical formula	C_10_H_15_N_2_^+^·C_7_H_3_N_2_O_7_^−^	C_10_H_15_N_2_^+^·C_7_H_3_N_2_O_6_^−^	C_10_H_15_N_2_^+^·C_6_H_2_N_3_O_7_^−^	C_10_H_15_N_2_^+^·C_7_H_5_O_2_^−^·H_2_O
*M*_r_	390.35	374.35	391.34	302.36
Crystal system, space group	Monoclinic, *P*2_1_/*c*	Triclinic, *P* 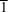	Monoclinic, *P*2_1_/*c*	Monoclinic, *P*2_1_/*c*
Temperature (K)	293	293	293	293
*a*, *b*, *c* (Å)	7.779 (3), 7.411 (3), 31.357 (9)	5.707 (2), 12.505 (3), 13.116 (3)	8.517 (1), 6.825 (1), 30.265 (4)	6.202 (2), 34.573 (9), 7.596 (2)
α, β, γ (°)	90, 96.82 (3), 90	97.41 (2), 93.28 (2), 102.82 (2)	90, 95.33 (1), 90	90, 93.83 (2), 90
*V* (Å^3^)	1794.9 (11)	901.5 (4)	1751.7 (4)	1625.1 (8)
*Z*	4	2	4	4
Radiation type	Mo *K*α	Mo *K*α	Mo *K*α	Mo *K*α
μ (mm^−1^)	0.11	0.11	0.12	0.09
Crystal size (mm)	0.20 × 0.18 × 0.12	0.48 × 0.08 × 0.04	0.50 × 0.36 × 0.20	0.32 × 0.20 × 0.16

Data collection				
Diffractometer	Oxford Diffraction Xcalibur with Sapphire CCD	Oxford Diffraction Xcalibur with Sapphire CCD	Oxford Diffraction Xcalibur with Sapphire CCD	Oxford Diffraction Xcalibur with Sapphire CCD
Absorption correction	Multi-scan (*CrysAlis RED*; Oxford Diffraction, 2007 ▸)	Multi-scan (*CrysAlis RED*; Oxford Diffraction, 2007 ▸)	Multi-scan (*CrysAlis RED*; Oxford Diffraction, 2007 ▸)	Multi-scan (*CrysAlis RED*; Oxford Diffraction, 2007 ▸)
*T*_min_, *T*_max_	0.959, 1.000	0.647, 1.000	0.835, 1.000	0.985, 1.000
No. of measured, independent and observed [*I* > 2σ(*I*)] reflections	7737, 3882, 1590	7800, 7800, 2647	12427, 3893, 2389	6075, 3492, 1387
*R*_int_	0.055	0.087	0.076	0.039
(sin θ/λ)_max_ (Å^−1^)	0.661	0.663	0.660	0.656

Refinement				
*R*[*F* ^2^ > 2σ(*F* ^2^)], *wR*(*F* ^2^), *S*	0.085, 0.155, 1.03	0.147, 0.297, 1.13	0.064, 0.149, 1.05	0.065, 0.144, 0.95
No. of reflections	3882	7800	3893	3492
No. of parameters	321	251	260	211
No. of restraints	288	2	0	4
H-atom treatment	H atoms treated by a mixture of independent and constrained refinement	H atoms treated by a mixture of independent and constrained refinement	H atoms treated by a mixture of independent and constrained refinement	H atoms treated by a mixture of independent and constrained refinement
Δρ_max_, Δρ_min_ (e Å^−3^)	0.18, −0.20	0.28, −0.30	0.26, −0.20	0.24, −0.16
Absolute structure	–	–	–	–
Absolute structure parameter	–	–	–	–

**Table:** 

	**10**	**11**	**12**
Crystal data			
Chemical formula	C_10_H_15_N_2_^+^·C_7_H_7_O_3_S^−^	C_10_H_15_N_2_^+^·C_4_H_5_O_6_^−^·H_2_O	C_10_H_15_N_2_^+^·C_4_H_3_O_4_^−^
*M*_r_	334.42	330.33	278.30
Crystal system, space group	Monoclinic, *P*2_1_	Orthorhombic, *P*2_1_2_1_2_1_	Orthorhombic, *P* *c* *a*2_1_
Temperature (K)	293	293	293
*a*, *b*, *c* (Å)	8.325 (1), 10.949 (2), 18.418 (4)	7.1185 (7), 7.5255 (8), 29.955 (3)	26.702 (1), 7.9626 (3), 6.7571 (3)
α, β, γ (°)	90, 92.67 (2), 90	90, 90, 90	90, 90, 90
*V* (Å^3^)	1677.0 (5)	1604.7 (3)	1436.68 (10)
*Z*	4	4	4
Radiation type	Mo *K*α	Mo *K*α	Mo *K*α
μ (mm^−1^)	0.21	0.11	0.10
Crystal size (mm)	0.50 × 0.36 × 0.14	0.42 × 0.32 × 0.24	0.48 × 0.44 × 0.40

Data collection			
Diffractometer	Oxford Diffraction Xcalibur with Sapphire CCD	Oxford Diffraction Xcalibur with Sapphire CCD	Oxford Diffraction Xcalibur with Sapphire CCD
Absorption correction	Multi-scan (*CrysAlis RED*; Oxford Diffraction, 2007 ▸)	Multi-scan (*CrysAlis RED*; Oxford Diffraction, 2007 ▸)	Multi-scan (*CrysAlis RED*; Oxford Diffraction, 2007 ▸)
*T*_min_, *T*_max_	0.696, 1.000	0.883, 1.000	0.894, 1.000
No. of measured, independent and observed [*I* > 2σ(*I*)] reflections	6123, 4918, 2767	6773, 3354, 2808	9534, 3127, 2770
*R*_int_	0.044	0.019	0.018
(sin θ/λ)_max_ (Å^−1^)	0.654	0.657	0.657

Refinement			
*R*[*F* ^2^ > 2σ(*F* ^2^)], *wR*(*F* ^2^), *S*	0.126, 0.298, 1.12	0.045, 0.100, 1.09	0.034, 0.077, 1.06
No. of reflections	4918	3354	3127
No. of parameters	480	260	191
No. of restraints	853	211	4
H-atom treatment	H-atom parameters constrained	H atoms treated by a mixture of independent and constrained refinement	H atoms treated by a mixture of independent and constrained refinement
Δρ_max_, Δρ_min_ (e Å^−3^)	1.08, −0.41	0.20, −0.16	0.17, −0.13
Absolute structure	Flack *x* determined using 597 quotients [(*I* ^+^)−(*I* ^−^)]/[(*I* ^+^)+(*I* ^−^)] (Parsons *et al.*, 2013 ▸)	Flack *x* determined using 912 quotients [(*I* ^+^)−(*I* ^−^)]/[(*I* ^+^)+(*I* ^−^)] (Parsons *et al.*, 2013 ▸)	Flack *x* determined using 1130 quotients [(*I* ^+^)−(*I* ^−^)]/[(*I* ^+^)+(*I* ^−^)] (Parsons *et al.*, 2013 ▸)
Absolute structure parameter	0.00 (11)	−0.2 (5)	0.3 (3)

Computer programs: *CrysAlis CCD* and *CrysAlis RED* (Oxford Diffraction, 2007 ▸), *SHELXT* (Sheldrick, 2015*a* ▸), *SHELXL* (Sheldrick, 2015*b* ▸) and *SHELXTL* (Sheldrick, 2008 ▸).

## supplementary crystallographic information

### 4-Phenylpiperazin-1-ium 4-fluorobenzoate monohydrate (1) Crystal data

**Table:**

C_10_H_15_N_2_^+^·C_7_H_4_FO_2_^−^·H_2_O	*Z* = 2
*M**_r_* = 320.36	*F*(000) = 340
Triclinic, *P*1	*D*_x_ = 1.286 Mg m^−^^3^
*a* = 6.239 (1) Å	Mo *K*α radiation, λ = 0.71073 Å
*b* = 7.496 (1) Å	Cell parameters from 1437 reflections
*c* = 17.817 (3) Å	θ = 2.8–27.7°
α = 93.55 (2)°	µ = 0.10 mm^−^^1^
β = 92.94 (2)°	*T* = 293 K
γ = 94.87 (2)°	Rod, colourless
*V* = 827.3 (2) Å^3^	0.44 × 0.32 × 0.16 mm

### 4-Phenylpiperazin-1-ium 4-fluorobenzoate monohydrate (1) Data collection

**Table:**

Oxford Diffraction Xcalibur with Sapphire CCD diffractometer	1611 reflections with *I* > 2σ(*I*)
Radiation source: Enhance (Mo) X-ray Source	*R*_int_ = 0.033
ω and φ scans	θ_max_ = 25.3°, θ_min_ = 2.9°
Absorption correction: multi-scan (CrysalisRED; Oxford Diffraction, 2007)	*h* = −7→7
*T*_min_ = 0.613, *T*_max_ = 1.000	*k* = −9→8
4669 measured reflections	*l* = −21→20
3013 independent reflections	

### 4-Phenylpiperazin-1-ium 4-fluorobenzoate monohydrate (1) Refinement

**Table:**

Refinement on *F*^2^	Primary atom site location: dual
Least-squares matrix: full	Secondary atom site location: difference Fourier map
*R*[*F*^2^ > 2σ(*F*^2^)] = 0.090	Hydrogen site location: mixed
*wR*(*F*^2^) = 0.226	H atoms treated by a mixture of independent and constrained refinement
*S* = 1.10	*w* = 1/[σ^2^(*F*_o_^2^) + (0.0717*P*)^2^ + 0.6694*P*] where *P* = (*F*_o_^2^ + 2*F*_c_^2^)/3
3013 reflections	(Δ/σ)_max_ < 0.001
220 parameters	Δρ_max_ = 0.27 e Å^−^^3^
4 restraints	Δρ_min_ = −0.20 e Å^−^^3^

### 4-Phenylpiperazin-1-ium 4-fluorobenzoate monohydrate (1) Special details

**Table:**

Geometry. All esds (except the esd in the dihedral angle between two l.s. planes) are estimated using the full covariance matrix. The cell esds are taken into account individually in the estimation of esds in distances, angles and torsion angles; correlations between esds in cell parameters are only used when they are defined by crystal symmetry. An approximate (isotropic) treatment of cell esds is used for estimating esds involving l.s. planes.

### 4-Phenylpiperazin-1-ium 4-fluorobenzoate monohydrate (1) Fractional atomic coordinates and isotropic or equivalent isotropic displacement parameters (Å^2^)

**Table:**

	*x*	*y*	*z*	*U*_iso_*/*U*_eq_	
N1	0.1673 (5)	0.6916 (4)	0.28754 (18)	0.0488 (8)	
N2	0.2637 (6)	0.7578 (5)	0.4474 (2)	0.0582 (10)	
H21	0.321 (6)	0.736 (6)	0.4932 (14)	0.070*	
H22	0.222 (6)	0.867 (3)	0.453 (2)	0.070*	
C1	0.1054 (6)	0.7113 (5)	0.2116 (2)	0.0517 (10)	
C2	−0.0937 (8)	0.6442 (7)	0.1796 (3)	0.0795 (15)	
H2	−0.193159	0.589826	0.210092	0.095*	
C3	−0.1510 (9)	0.6546 (8)	0.1045 (3)	0.1007 (19)	
H3	−0.285388	0.604546	0.084997	0.121*	
C4	−0.0122 (11)	0.7376 (9)	0.0585 (3)	0.1030 (19)	
H4	−0.049909	0.745192	0.007649	0.124*	
C5	0.1822 (10)	0.8090 (10)	0.0887 (3)	0.122 (3)	
H5	0.278723	0.865814	0.057892	0.147*	
C6	0.2409 (9)	0.7997 (8)	0.1637 (3)	0.0979 (19)	
H6	0.373978	0.853592	0.182809	0.117*	
C7	0.3620 (6)	0.8019 (6)	0.3177 (2)	0.0608 (11)	
H7A	0.332558	0.927034	0.320838	0.073*	
H7B	0.476070	0.788623	0.283301	0.073*	
C8	0.4378 (7)	0.7509 (6)	0.3946 (2)	0.0616 (12)	
H8A	0.484338	0.630572	0.390672	0.074*	
H8B	0.560050	0.832523	0.413731	0.074*	
C9	0.0695 (7)	0.6398 (6)	0.4170 (2)	0.0599 (11)	
H9A	−0.045974	0.649597	0.451027	0.072*	
H9B	0.103679	0.515781	0.413417	0.072*	
C10	−0.0033 (6)	0.6933 (6)	0.3403 (2)	0.0558 (11)	
H10A	−0.124568	0.611513	0.320378	0.067*	
H10B	−0.051786	0.812879	0.345074	0.067*	
F1	0.5316 (7)	0.7684 (6)	0.94599 (18)	0.1434 (15)	
O1	0.1448 (6)	0.8383 (6)	0.6190 (2)	0.1136 (14)	
O2	0.4185 (6)	0.6849 (4)	0.59104 (18)	0.0761 (10)	
C11	0.3677 (6)	0.7628 (5)	0.7197 (2)	0.0519 (10)	
C12	0.5515 (7)	0.6900 (6)	0.7457 (2)	0.0605 (11)	
H12	0.639144	0.638731	0.711219	0.073*	
C13	0.6074 (8)	0.6918 (7)	0.8214 (3)	0.0777 (14)	
H13	0.731513	0.642435	0.838182	0.093*	
C14	0.4782 (10)	0.7668 (8)	0.8711 (3)	0.0885 (16)	
C15	0.2959 (10)	0.8423 (8)	0.8486 (3)	0.0890 (16)	
H15	0.210580	0.894321	0.883627	0.107*	
C16	0.2416 (7)	0.8393 (6)	0.7725 (3)	0.0723 (13)	
H16	0.117563	0.889726	0.756336	0.087*	
C17	0.3049 (7)	0.7617 (6)	0.6375 (3)	0.0609 (12)	
O3	0.7825 (5)	0.8718 (5)	0.5423 (2)	0.0747 (10)	
H31	0.898 (5)	0.857 (8)	0.565 (3)	0.112*	
H32	0.674 (6)	0.829 (8)	0.562 (3)	0.112*	

### 4-Phenylpiperazin-1-ium 4-fluorobenzoate monohydrate (1) Atomic displacement parameters (Å^2^)

**Table:**

	*U*^11^	*U*^22^	*U*^33^	*U*^12^	*U*^13^	*U*^23^
N1	0.0464 (19)	0.0462 (19)	0.053 (2)	−0.0029 (14)	0.0061 (15)	0.0075 (14)
N2	0.072 (2)	0.048 (2)	0.055 (2)	0.0064 (18)	−0.0066 (19)	0.0061 (17)
C1	0.058 (3)	0.048 (2)	0.048 (3)	0.0003 (19)	0.005 (2)	0.0056 (18)
C2	0.079 (3)	0.094 (4)	0.060 (3)	−0.022 (3)	−0.005 (3)	0.017 (3)
C3	0.104 (4)	0.118 (5)	0.070 (4)	−0.036 (4)	−0.021 (3)	0.017 (3)
C4	0.129 (5)	0.120 (5)	0.056 (3)	−0.015 (4)	−0.006 (3)	0.014 (3)
C5	0.116 (5)	0.185 (7)	0.059 (4)	−0.045 (5)	0.008 (3)	0.029 (4)
C6	0.088 (4)	0.141 (5)	0.058 (3)	−0.039 (4)	0.003 (3)	0.020 (3)
C7	0.049 (2)	0.072 (3)	0.059 (3)	−0.009 (2)	0.004 (2)	0.009 (2)
C8	0.053 (3)	0.065 (3)	0.065 (3)	0.000 (2)	−0.002 (2)	0.003 (2)
C9	0.064 (3)	0.056 (3)	0.058 (3)	−0.008 (2)	0.005 (2)	0.007 (2)
C10	0.049 (2)	0.058 (3)	0.060 (3)	−0.0019 (19)	0.006 (2)	0.006 (2)
F1	0.180 (4)	0.187 (4)	0.060 (2)	0.003 (3)	−0.002 (2)	0.004 (2)
O1	0.090 (3)	0.140 (4)	0.114 (3)	0.037 (3)	−0.027 (2)	0.029 (3)
O2	0.097 (2)	0.069 (2)	0.061 (2)	0.0018 (18)	−0.0046 (18)	0.0101 (16)
C11	0.049 (2)	0.041 (2)	0.066 (3)	−0.0054 (18)	0.007 (2)	0.0126 (19)
C12	0.062 (3)	0.056 (3)	0.062 (3)	0.004 (2)	−0.001 (2)	0.006 (2)
C13	0.082 (3)	0.083 (4)	0.067 (3)	0.009 (3)	−0.009 (3)	0.010 (3)
C14	0.105 (4)	0.098 (4)	0.059 (3)	−0.005 (3)	−0.002 (3)	0.004 (3)
C15	0.097 (4)	0.093 (4)	0.079 (4)	0.004 (3)	0.031 (3)	0.001 (3)
C16	0.057 (3)	0.066 (3)	0.096 (4)	0.002 (2)	0.012 (3)	0.018 (3)
C17	0.056 (3)	0.049 (3)	0.076 (3)	−0.012 (2)	−0.009 (2)	0.020 (2)
O3	0.071 (2)	0.065 (2)	0.088 (3)	0.0124 (18)	−0.0088 (18)	0.0039 (17)

### 4-Phenylpiperazin-1-ium 4-fluorobenzoate monohydrate (1) Geometric parameters (Å, º)

**Table:**

N1—C1	1.408 (5)	C8—H8B	0.9700
N1—C10	1.456 (5)	C9—C10	1.507 (5)
N1—C7	1.468 (5)	C9—H9A	0.9700
N2—C8	1.475 (5)	C9—H9B	0.9700
N2—C9	1.495 (5)	C10—H10A	0.9700
N2—H21	0.904 (19)	C10—H10B	0.9700
N2—H22	0.881 (19)	F1—C14	1.358 (6)
C1—C2	1.381 (6)	O1—C17	1.235 (5)
C1—C6	1.389 (6)	O2—C17	1.261 (5)
C2—C3	1.377 (6)	C11—C12	1.382 (5)
C2—H2	0.9300	C11—C16	1.386 (6)
C3—C4	1.361 (8)	C11—C17	1.496 (6)
C3—H3	0.9300	C12—C13	1.375 (6)
C4—C5	1.355 (8)	C12—H12	0.9300
C4—H4	0.9300	C13—C14	1.356 (7)
C5—C6	1.375 (7)	C13—H13	0.9300
C5—H5	0.9300	C14—C15	1.366 (7)
C6—H6	0.9300	C15—C16	1.377 (7)
C7—C8	1.509 (5)	C15—H15	0.9300
C7—H7A	0.9700	C16—H16	0.9300
C7—H7B	0.9700	O3—H31	0.83 (2)
C8—H8A	0.9700	O3—H32	0.83 (2)
			
C1—N1—C10	116.2 (3)	C7—C8—H8B	109.5
C1—N1—C7	115.9 (3)	H8A—C8—H8B	108.1
C10—N1—C7	111.5 (3)	N2—C9—C10	110.4 (3)
C8—N2—C9	110.3 (3)	N2—C9—H9A	109.6
C8—N2—H21	107 (3)	C10—C9—H9A	109.6
C9—N2—H21	117 (3)	N2—C9—H9B	109.6
C8—N2—H22	111 (3)	C10—C9—H9B	109.6
C9—N2—H22	106 (3)	H9A—C9—H9B	108.1
H21—N2—H22	105 (4)	N1—C10—C9	112.3 (3)
C2—C1—C6	115.5 (4)	N1—C10—H10A	109.1
C2—C1—N1	122.4 (4)	C9—C10—H10A	109.1
C6—C1—N1	122.2 (4)	N1—C10—H10B	109.1
C3—C2—C1	122.7 (5)	C9—C10—H10B	109.1
C3—C2—H2	118.7	H10A—C10—H10B	107.9
C1—C2—H2	118.7	C12—C11—C16	117.8 (4)
C4—C3—C2	120.5 (5)	C12—C11—C17	121.9 (4)
C4—C3—H3	119.8	C16—C11—C17	120.2 (4)
C2—C3—H3	119.8	C13—C12—C11	121.4 (4)
C5—C4—C3	118.2 (5)	C13—C12—H12	119.3
C5—C4—H4	120.9	C11—C12—H12	119.3
C3—C4—H4	120.9	C14—C13—C12	118.8 (5)
C4—C5—C6	121.8 (5)	C14—C13—H13	120.6
C4—C5—H5	119.1	C12—C13—H13	120.6
C6—C5—H5	119.1	C13—C14—F1	119.2 (6)
C5—C6—C1	121.3 (5)	C13—C14—C15	122.3 (5)
C5—C6—H6	119.4	F1—C14—C15	118.5 (6)
C1—C6—H6	119.4	C14—C15—C16	118.3 (5)
N1—C7—C8	112.4 (3)	C14—C15—H15	120.9
N1—C7—H7A	109.1	C16—C15—H15	120.9
C8—C7—H7A	109.1	C15—C16—C11	121.4 (5)
N1—C7—H7B	109.1	C15—C16—H16	119.3
C8—C7—H7B	109.1	C11—C16—H16	119.3
H7A—C7—H7B	107.9	O1—C17—O2	123.6 (5)
N2—C8—C7	110.8 (3)	O1—C17—C11	117.7 (5)
N2—C8—H8A	109.5	O2—C17—C11	118.7 (4)
C7—C8—H8A	109.5	H31—O3—H32	115 (6)
N2—C8—H8B	109.5		
			
C10—N1—C1—C2	−33.4 (6)	C1—N1—C10—C9	170.1 (3)
C7—N1—C1—C2	−167.2 (4)	C7—N1—C10—C9	−54.1 (4)
C10—N1—C1—C6	146.6 (5)	N2—C9—C10—N1	55.9 (4)
C7—N1—C1—C6	12.8 (6)	C16—C11—C12—C13	0.5 (6)
C6—C1—C2—C3	3.5 (8)	C17—C11—C12—C13	−180.0 (4)
N1—C1—C2—C3	−176.4 (5)	C11—C12—C13—C14	0.0 (7)
C1—C2—C3—C4	−1.9 (9)	C12—C13—C14—F1	179.7 (4)
C2—C3—C4—C5	0.0 (10)	C12—C13—C14—C15	−0.7 (8)
C3—C4—C5—C6	−0.2 (11)	C13—C14—C15—C16	0.8 (9)
C4—C5—C6—C1	2.1 (11)	F1—C14—C15—C16	−179.6 (4)
C2—C1—C6—C5	−3.6 (8)	C14—C15—C16—C11	−0.3 (8)
N1—C1—C6—C5	176.4 (6)	C12—C11—C16—C15	−0.4 (6)
C1—N1—C7—C8	−170.5 (3)	C17—C11—C16—C15	−179.9 (4)
C10—N1—C7—C8	53.5 (5)	C12—C11—C17—O1	−176.1 (4)
C9—N2—C8—C7	56.3 (4)	C16—C11—C17—O1	3.4 (6)
N1—C7—C8—N2	−55.1 (5)	C12—C11—C17—O2	3.2 (6)
C8—N2—C9—C10	−56.6 (4)	C16—C11—C17—O2	−177.3 (4)

### 4-Phenylpiperazin-1-ium 4-fluorobenzoate monohydrate (1) Hydrogen-bond geometry (Å, º)

**Table:**

*D*—H···*A*	*D*—H	H···*A*	*D*···*A*	*D*—H···*A*
N2—H21···O1	0.90 (2)	2.65 (4)	3.215 (6)	122 (3)
N2—H21···O2	0.90 (2)	1.89 (2)	2.791 (5)	175 (4)
N2—H22···O3^i^	0.88 (2)	1.96 (2)	2.812 (5)	163 (4)
C8—H8*A*···O2^ii^	0.97	2.53	3.481 (6)	168
C8—H8*B*···O3	0.97	2.60	3.341 (5)	133
C9—H9*A*···O3^iii^	0.97	2.59	3.416 (6)	143
O3—H31···O1^iv^	0.83 (2)	1.79 (2)	2.619 (5)	176 (6)
O3—H32···O2	0.83 (2)	1.96 (2)	2.773 (5)	167 (6)

Symmetry codes: (i) −*x*+1, −*y*+2, −*z*+1; (ii) −*x*+1, −*y*+1, −*z*+1; (iii) *x*−1, *y*, *z*; (iv) *x*+1, *y*, *z*.

### 4-Phenylpiperazin-1-ium 4-bromobenzoate monohydrate (3) Crystal data

**Table:**

C_10_H_15_N_2_^+^·C_7_H_4_BrO_2_^−^·H_2_O	*F*(000) = 784
*M**_r_* = 381.27	*D*_x_ = 1.449 Mg m^−^^3^
Monoclinic, *P*2_1_/*c*	Mo *K*α radiation, λ = 0.71073 Å
*a* = 6.183 (2) Å	Cell parameters from 1437 reflections
*b* = 37.748 (7) Å	θ = 2.8–27.7°
*c* = 7.506 (2) Å	µ = 2.37 mm^−^^1^
β = 93.69 (4)°	*T* = 293 K
*V* = 1748.2 (8) Å^3^	Rod, colourless
*Z* = 4	0.46 × 0.20 × 0.12 mm

### 4-Phenylpiperazin-1-ium 4-bromobenzoate monohydrate (3) Data collection

**Table:**

Oxford Diffraction Xcalibur with Sapphire CCD diffractometer	1374 reflections with *I* > 2σ(*I*)
Radiation source: Enhance (Mo) X-ray Source	*R*_int_ = 0.061
ω and φ scans	θ_max_ = 25.4°, θ_min_ = 2.8°
Absorption correction: multi-scan (CrysalisRED; Oxford Diffraction, 2007)	*h* = −4→7
*T*_min_ = 0.613, *T*_max_ = 1.000	*k* = −45→24
6103 measured reflections	*l* = −8→9
3170 independent reflections	

### 4-Phenylpiperazin-1-ium 4-bromobenzoate monohydrate (3) Refinement

**Table:**

Refinement on *F*^2^	Primary atom site location: dual
Least-squares matrix: full	Secondary atom site location: difference Fourier map
*R*[*F*^2^ > 2σ(*F*^2^)] = 0.138	Hydrogen site location: mixed
*wR*(*F*^2^) = 0.375	H atoms treated by a mixture of independent and constrained refinement
*S* = 1.03	*w* = 1/[σ^2^(*F*_o_^2^) + (0.1282*P*)^2^ + 10.0601*P*] where *P* = (*F*_o_^2^ + 2*F*_c_^2^)/3
3170 reflections	(Δ/σ)_max_ = 0.003
215 parameters	Δρ_max_ = 0.49 e Å^−^^3^
7 restraints	Δρ_min_ = −0.61 e Å^−^^3^

### 4-Phenylpiperazin-1-ium 4-bromobenzoate monohydrate (3) Special details

**Table:**

Geometry. All esds (except the esd in the dihedral angle between two l.s. planes) are estimated using the full covariance matrix. The cell esds are taken into account individually in the estimation of esds in distances, angles and torsion angles; correlations between esds in cell parameters are only used when they are defined by crystal symmetry. An approximate (isotropic) treatment of cell esds is used for estimating esds involving l.s. planes.

### 4-Phenylpiperazin-1-ium 4-bromobenzoate monohydrate (3) Fractional atomic coordinates and isotropic or equivalent isotropic displacement parameters (Å^2^)

**Table:**

	*x*	*y*	*z*	*U*_iso_*/*U*_eq_	Occ. (<1)
N1	0.7034 (12)	0.4002 (2)	0.2295 (10)	0.047 (2)	
N2	0.7744 (14)	0.4754 (2)	0.2661 (11)	0.057 (2)	
H2N1	0.816948	0.495983	0.221941	0.068*	0.68 (16)
H2N2	0.734325	0.479226	0.376244	0.068*	
C1	0.6526 (17)	0.3649 (3)	0.2644 (14)	0.052 (3)	
C2	0.4510 (19)	0.3499 (3)	0.2008 (17)	0.077 (4)	
H2	0.347995	0.364177	0.140653	0.092*	
C3	0.406 (2)	0.3151 (4)	0.226 (2)	0.095 (4)	
H3	0.275365	0.305737	0.179024	0.114*	
C4	0.552 (3)	0.2938 (4)	0.320 (2)	0.101 (5)	
H4	0.518855	0.270118	0.340272	0.122*	
C5	0.745 (3)	0.3071 (4)	0.385 (2)	0.109 (5)	
H5	0.846262	0.292510	0.444957	0.131*	
C6	0.790 (2)	0.3418 (3)	0.361 (2)	0.091 (4)	
H6	0.919874	0.350644	0.411615	0.109*	
C7	0.8936 (15)	0.4148 (3)	0.3321 (15)	0.056 (3)	
H7A	0.863266	0.416060	0.457161	0.067*	
H7B	1.015626	0.398972	0.321899	0.067*	
C8	0.9530 (18)	0.4502 (3)	0.2711 (16)	0.066 (3)	
H8A	1.070611	0.459306	0.349995	0.080*	
H8B	1.005544	0.448322	0.152505	0.080*	
C9	0.5886 (17)	0.4613 (3)	0.1532 (15)	0.063 (3)	
H9A	0.628083	0.459435	0.030616	0.075*	
H9B	0.466823	0.477483	0.156218	0.075*	
C10	0.5255 (17)	0.4260 (3)	0.2189 (14)	0.057 (3)	
H10A	0.471137	0.428689	0.336512	0.069*	
H10B	0.408395	0.416774	0.140070	0.069*	
Br1	0.9708 (5)	0.72340 (4)	0.1736 (4)	0.1674 (14)	
O1	0.6289 (16)	0.5558 (3)	0.3222 (15)	0.114 (4)	
O2	0.9081 (15)	0.5426 (2)	0.1687 (11)	0.077 (2)	
H2O	0.853628	0.522821	0.170738	0.093*	0.32 (16)
C11	0.8325 (17)	0.6031 (3)	0.2219 (13)	0.053 (3)	
C12	1.0163 (19)	0.6155 (3)	0.1465 (14)	0.063 (3)	
H12	1.115409	0.599557	0.104078	0.076*	
C13	1.053 (2)	0.6508 (3)	0.1343 (16)	0.072 (3)	
H13	1.177707	0.658367	0.083258	0.086*	
C14	0.919 (3)	0.6747 (4)	0.1911 (19)	0.088 (4)	
C15	0.741 (3)	0.6638 (4)	0.277 (2)	0.097 (5)	
H15	0.650657	0.680550	0.324796	0.116*	
C16	0.694 (2)	0.6281 (4)	0.2929 (17)	0.080 (4)	
H16	0.572770	0.620785	0.349992	0.096*	
C17	0.787 (2)	0.5643 (4)	0.2376 (14)	0.062 (3)	
O3	0.7207 (13)	0.4796 (2)	0.6335 (11)	0.073 (2)	
H31	0.830 (14)	0.4755 (18)	0.696 (17)	0.109*	
H32	0.697 (5)	0.5004 (5)	0.635 (5)	0.109*	

### 4-Phenylpiperazin-1-ium 4-bromobenzoate monohydrate (3) Atomic displacement parameters (Å^2^)

**Table:**

	*U*^11^	*U*^22^	*U*^33^	*U*^12^	*U*^13^	*U*^23^
N1	0.044 (5)	0.050 (5)	0.046 (5)	0.003 (4)	0.002 (4)	−0.005 (4)
N2	0.063 (6)	0.056 (5)	0.052 (5)	−0.003 (5)	0.011 (4)	0.000 (4)
C1	0.054 (6)	0.053 (6)	0.048 (6)	0.007 (5)	−0.001 (5)	−0.010 (5)
C2	0.066 (8)	0.085 (9)	0.076 (8)	−0.005 (7)	−0.021 (7)	0.021 (7)
C3	0.092 (10)	0.077 (9)	0.113 (12)	−0.021 (8)	−0.018 (9)	0.004 (9)
C4	0.119 (13)	0.067 (9)	0.116 (12)	−0.010 (9)	−0.015 (10)	0.016 (9)
C5	0.127 (13)	0.062 (9)	0.134 (14)	0.012 (9)	−0.021 (11)	0.010 (9)
C6	0.085 (9)	0.068 (8)	0.117 (12)	−0.004 (7)	−0.015 (8)	0.000 (8)
C7	0.039 (6)	0.060 (7)	0.068 (7)	0.003 (5)	0.000 (5)	−0.003 (6)
C8	0.056 (7)	0.084 (9)	0.061 (7)	−0.004 (6)	0.010 (6)	0.004 (6)
C9	0.054 (7)	0.068 (7)	0.066 (7)	0.003 (6)	0.005 (6)	0.006 (6)
C10	0.065 (7)	0.060 (7)	0.045 (6)	0.004 (6)	−0.012 (5)	−0.003 (5)
Br1	0.266 (3)	0.0626 (11)	0.169 (2)	−0.0154 (13)	−0.020 (2)	−0.0006 (12)
O1	0.091 (7)	0.131 (9)	0.123 (8)	−0.028 (6)	0.036 (7)	0.037 (7)
O2	0.098 (6)	0.063 (5)	0.072 (6)	−0.005 (5)	0.015 (5)	0.005 (4)
C11	0.052 (6)	0.074 (7)	0.033 (5)	0.004 (6)	0.006 (5)	−0.005 (5)
C12	0.069 (7)	0.074 (8)	0.047 (6)	0.004 (6)	0.008 (6)	−0.003 (6)
C13	0.085 (9)	0.069 (8)	0.060 (8)	−0.015 (7)	−0.003 (6)	0.000 (7)
C14	0.128 (13)	0.067 (8)	0.071 (9)	0.011 (9)	0.012 (9)	0.006 (7)
C15	0.110 (12)	0.090 (11)	0.089 (10)	0.036 (9)	0.006 (10)	−0.016 (9)
C16	0.079 (9)	0.102 (11)	0.060 (7)	0.006 (8)	0.010 (6)	0.009 (8)
C17	0.058 (7)	0.089 (9)	0.037 (6)	−0.009 (7)	−0.013 (5)	0.008 (6)
O3	0.081 (6)	0.073 (5)	0.064 (5)	−0.015 (4)	0.010 (4)	−0.006 (4)

### 4-Phenylpiperazin-1-ium 4-bromobenzoate monohydrate (3) Geometric parameters (Å, º)

**Table:**

N1—C1	1.398 (12)	C9—C10	1.483 (14)
N1—C10	1.467 (12)	C9—H9A	0.9700
N1—C7	1.471 (12)	C9—H9B	0.9700
N2—C8	1.456 (13)	C10—H10A	0.9700
N2—C9	1.481 (13)	C10—H10B	0.9700
N2—H2N1	0.8900	Br1—C14	1.872 (14)
N2—H2N2	0.8900	O1—C17	1.242 (13)
C1—C6	1.387 (16)	O2—C17	1.244 (14)
C1—C2	1.424 (15)	O2—H2O	0.8200
C2—C3	1.356 (17)	C11—C12	1.384 (14)
C2—H2	0.9300	C11—C16	1.402 (16)
C3—C4	1.38 (2)	C11—C17	1.495 (16)
C3—H3	0.9300	C12—C13	1.354 (16)
C4—C5	1.35 (2)	C12—H12	0.9300
C4—H4	0.9300	C13—C14	1.312 (18)
C5—C6	1.352 (17)	C13—H13	0.9300
C5—H5	0.9300	C14—C15	1.38 (2)
C6—H6	0.9300	C15—C16	1.385 (19)
C7—C8	1.467 (14)	C15—H15	0.9300
C7—H7A	0.9700	C16—H16	0.9300
C7—H7B	0.9700	O3—H31	0.81 (2)
C8—H8A	0.9700	O3—H32	0.796 (19)
C8—H8B	0.9700		
			
C1—N1—C10	117.8 (8)	C7—C8—H8B	108.9
C1—N1—C7	116.2 (8)	H8A—C8—H8B	107.7
C10—N1—C7	110.5 (7)	N2—C9—C10	110.1 (9)
C8—N2—C9	109.8 (8)	N2—C9—H9A	109.6
C8—N2—H2N1	109.7	C10—C9—H9A	109.6
C9—N2—H2N1	109.7	N2—C9—H9B	109.6
C8—N2—H2N2	109.7	C10—C9—H9B	109.6
C9—N2—H2N2	109.7	H9A—C9—H9B	108.2
H2N1—N2—H2N2	108.2	N1—C10—C9	113.8 (9)
C6—C1—N1	124.0 (10)	N1—C10—H10A	108.8
C6—C1—C2	114.6 (10)	C9—C10—H10A	108.8
N1—C1—C2	121.3 (9)	N1—C10—H10B	108.8
C3—C2—C1	121.5 (11)	C9—C10—H10B	108.8
C3—C2—H2	119.3	H10A—C10—H10B	107.7
C1—C2—H2	119.3	C17—O2—H2O	109.5
C2—C3—C4	120.3 (13)	C12—C11—C16	117.6 (11)
C2—C3—H3	119.8	C12—C11—C17	121.9 (10)
C4—C3—H3	119.8	C16—C11—C17	120.4 (11)
C5—C4—C3	120.0 (14)	C13—C12—C11	120.4 (11)
C5—C4—H4	120.0	C13—C12—H12	119.8
C3—C4—H4	120.0	C11—C12—H12	119.8
C6—C5—C4	119.8 (15)	C14—C13—C12	123.0 (13)
C6—C5—H5	120.1	C14—C13—H13	118.5
C4—C5—H5	120.1	C12—C13—H13	118.5
C5—C6—C1	123.7 (13)	C13—C14—C15	119.1 (13)
C5—C6—H6	118.2	C13—C14—Br1	122.7 (13)
C1—C6—H6	118.2	C15—C14—Br1	118.1 (12)
C8—C7—N1	112.7 (9)	C14—C15—C16	120.5 (13)
C8—C7—H7A	109.0	C14—C15—H15	119.7
N1—C7—H7A	109.0	C16—C15—H15	119.7
C8—C7—H7B	109.0	C15—C16—C11	119.2 (12)
N1—C7—H7B	109.0	C15—C16—H16	120.4
H7A—C7—H7B	107.8	C11—C16—H16	120.4
N2—C8—C7	113.5 (9)	O1—C17—O2	123.8 (12)
N2—C8—H8A	108.9	O1—C17—C11	116.9 (12)
C7—C8—H8A	108.9	O2—C17—C11	119.3 (10)
N2—C8—H8B	108.9	H31—O3—H32	109 (4)
			
C10—N1—C1—C6	−147.2 (11)	C1—N1—C10—C9	−170.7 (9)
C7—N1—C1—C6	−12.7 (15)	C7—N1—C10—C9	52.4 (11)
C10—N1—C1—C2	33.1 (14)	N2—C9—C10—N1	−55.9 (12)
C7—N1—C1—C2	167.6 (10)	C16—C11—C12—C13	−3.5 (15)
C6—C1—C2—C3	−3.4 (18)	C17—C11—C12—C13	−180.0 (10)
N1—C1—C2—C3	176.3 (12)	C11—C12—C13—C14	−0.2 (19)
C1—C2—C3—C4	3 (2)	C12—C13—C14—C15	4 (2)
C2—C3—C4—C5	−2 (3)	C12—C13—C14—Br1	−180.0 (9)
C3—C4—C5—C6	2 (3)	C13—C14—C15—C16	−4 (2)
C4—C5—C6—C1	−3 (3)	Br1—C14—C15—C16	179.6 (11)
N1—C1—C6—C5	−175.8 (14)	C14—C15—C16—C11	1 (2)
C2—C1—C6—C5	4 (2)	C12—C11—C16—C15	3.2 (17)
C1—N1—C7—C8	172.4 (9)	C17—C11—C16—C15	179.7 (11)
C10—N1—C7—C8	−50.0 (11)	C12—C11—C17—O1	173.4 (10)
C9—N2—C8—C7	−55.9 (12)	C16—C11—C17—O1	−3.0 (15)
N1—C7—C8—N2	53.6 (12)	C12—C11—C17—O2	−5.8 (15)
C8—N2—C9—C10	55.7 (11)	C16—C11—C17—O2	177.8 (10)

### 4-Phenylpiperazin-1-ium 4-bromobenzoate monohydrate (3) Hydrogen-bond geometry (Å, º)

**Table:**

*D*—H···*A*	*D*—H	H···*A*	*D*···*A*	*D*—H···*A*
N2—H2*N*1···O2	0.89	1.90	2.780 (11)	170
N2—H2*N*2···O3	0.89	1.94	2.803 (12)	164
C8—H8*A*···O3^i^	0.97	2.64	3.377 (14)	133
C8—H8*B*···O2^ii^	0.97	2.53	3.475 (14)	166
C9—H9*B*···O3^iii^	0.97	2.59	3.403 (14)	142
O2—H2*O*···N2	0.82	2.00	2.780 (11)	159
O3—H31···O2^i^	0.81 (2)	1.98 (2)	2.782 (12)	170 (7)

Symmetry codes: (i) −*x*+2, −*y*+1, −*z*+1; (ii) −*x*+2, −*y*+1, −*z*; (iii) −*x*+1, −*y*+1, −*z*+1.

### 4-Phenylpiperazin-1-ium 4-iodobenzoate (4) Crystal data

**Table:**

C_10_H_15_N_2_^+^·C_7_H_4_IO_2_^−^	*F*(000) = 1632
*M**_r_* = 410.24	*D*_x_ = 1.652 Mg m^−^^3^
Monoclinic, *P*2_1_/*c*	Mo *K*α radiation, λ = 0.71073 Å
*a* = 10.8507 (4) Å	Cell parameters from 6801 reflections
*b* = 23.4045 (7) Å	θ = 2.6–27.8°
*c* = 13.3019 (4) Å	µ = 1.95 mm^−^^1^
β = 102.491 (4)°	*T* = 293 K
*V* = 3298.13 (19) Å^3^	Prism, colourless
*Z* = 8	0.48 × 0.48 × 0.40 mm

### 4-Phenylpiperazin-1-ium 4-iodobenzoate (4) Data collection

**Table:**

Oxford Diffraction Xcalibur with Sapphire CCD diffractometer	4641 reflections with *I* > 2σ(*I*)
Radiation source: Enhance (Mo) X-ray Source	*R*_int_ = 0.024
Rotation method data acquisition using ω scans.	θ_max_ = 27.8°, θ_min_ = 2.8°
Absorption correction: multi-scan (CrysalisRED; Oxford Diffraction, 2007)	*h* = −7→13
*T*_min_ = 0.575, *T*_max_ = 1.000	*k* = −30→15
14154 measured reflections	*l* = −17→17
7079 independent reflections	

### 4-Phenylpiperazin-1-ium 4-iodobenzoate (4) Refinement

**Table:**

Refinement on *F*^2^	Primary atom site location: dual
Least-squares matrix: full	Secondary atom site location: difference Fourier map
*R*[*F*^2^ > 2σ(*F*^2^)] = 0.037	Hydrogen site location: mixed
*wR*(*F*^2^) = 0.084	H atoms treated by a mixture of independent and constrained refinement
*S* = 1.02	*w* = 1/[σ^2^(*F*_o_^2^) + (0.0402*P*)^2^] where *P* = (*F*_o_^2^ + 2*F*_c_^2^)/3
7079 reflections	(Δ/σ)_max_ = 0.002
409 parameters	Δρ_max_ = 0.77 e Å^−^^3^
4 restraints	Δρ_min_ = −1.25 e Å^−^^3^

### 4-Phenylpiperazin-1-ium 4-iodobenzoate (4) Special details

**Table:**

Geometry. All esds (except the esd in the dihedral angle between two l.s. planes) are estimated using the full covariance matrix. The cell esds are taken into account individually in the estimation of esds in distances, angles and torsion angles; correlations between esds in cell parameters are only used when they are defined by crystal symmetry. An approximate (isotropic) treatment of cell esds is used for estimating esds involving l.s. planes.

### 4-Phenylpiperazin-1-ium 4-iodobenzoate (4) Fractional atomic coordinates and isotropic or equivalent isotropic displacement parameters (Å^2^)

**Table:**

	*x*	*y*	*z*	*U*_iso_*/*U*_eq_	
C1	0.6237 (3)	0.57603 (14)	0.0843 (2)	0.0363 (8)	
C2	0.4969 (3)	0.58795 (15)	0.0505 (3)	0.0431 (9)	
H2	0.437868	0.568638	0.079160	0.052*	
C3	0.4565 (4)	0.62827 (17)	−0.0255 (3)	0.0535 (10)	
H3	0.370693	0.635548	−0.047848	0.064*	
C4	0.5422 (4)	0.65774 (17)	−0.0684 (3)	0.0587 (11)	
H4	0.514944	0.685086	−0.119018	0.070*	
C5	0.6689 (4)	0.64613 (17)	−0.0351 (3)	0.0578 (11)	
H5	0.727710	0.665869	−0.063350	0.069*	
C6	0.7087 (3)	0.60578 (16)	0.0390 (3)	0.0488 (9)	
H6	0.794547	0.598015	0.059728	0.059*	
C7	0.5780 (3)	0.49980 (14)	0.1955 (2)	0.0399 (8)	
H7A	0.522273	0.482727	0.136209	0.048*	
H7B	0.527435	0.523672	0.230538	0.048*	
C8	0.6407 (3)	0.45316 (15)	0.2676 (3)	0.0461 (9)	
H8A	0.576921	0.431322	0.291939	0.055*	
H8B	0.685619	0.427387	0.231077	0.055*	
C9	0.8241 (3)	0.51415 (16)	0.3220 (3)	0.0506 (10)	
H9A	0.875290	0.490380	0.287377	0.061*	
H9B	0.879334	0.531571	0.381165	0.061*	
C10	0.7615 (3)	0.56009 (15)	0.2497 (3)	0.0437 (9)	
H10A	0.716702	0.586020	0.286126	0.052*	
H10B	0.825224	0.581826	0.225187	0.052*	
C11	0.1383 (3)	0.57882 (14)	0.0931 (3)	0.0378 (8)	
C12	0.0736 (3)	0.62745 (16)	0.1107 (3)	0.0500 (10)	
H12	0.060684	0.634674	0.176404	0.060*	
C13	0.0276 (4)	0.66576 (16)	0.0312 (3)	0.0604 (11)	
H13	−0.014418	0.698638	0.044310	0.072*	
C14	0.0443 (4)	0.65500 (19)	−0.0669 (3)	0.0609 (12)	
H14	0.012708	0.680304	−0.120126	0.073*	
C15	0.1071 (3)	0.60727 (17)	−0.0854 (3)	0.0519 (10)	
H15	0.117968	0.600061	−0.151708	0.062*	
C16	0.1549 (3)	0.56928 (15)	−0.0072 (3)	0.0435 (9)	
H16	0.198547	0.537080	−0.021249	0.052*	
C17	0.1510 (4)	0.48047 (15)	0.1505 (3)	0.0540 (10)	
H17A	0.154730	0.470809	0.080323	0.065*	
H17B	0.064339	0.476153	0.157480	0.065*	
C18	0.2346 (4)	0.44063 (16)	0.2238 (3)	0.0516 (10)	
H18A	0.203871	0.401837	0.210497	0.062*	
H18B	0.319385	0.442244	0.211451	0.062*	
C19	0.1817 (3)	0.55429 (15)	0.2758 (3)	0.0441 (9)	
H19A	0.094917	0.550003	0.282589	0.053*	
H19B	0.206022	0.593865	0.289794	0.053*	
C20	0.2659 (3)	0.51623 (15)	0.3526 (3)	0.0480 (9)	
H20A	0.353238	0.523724	0.350540	0.058*	
H20B	0.254904	0.525406	0.421236	0.058*	
C21	0.5418 (3)	0.35843 (13)	0.5875 (2)	0.0330 (7)	
C22	0.6116 (3)	0.31980 (14)	0.5442 (3)	0.0403 (8)	
H22	0.609882	0.321699	0.474075	0.048*	
C23	0.6838 (3)	0.27831 (14)	0.6037 (3)	0.0421 (9)	
H23	0.730730	0.252638	0.573877	0.050*	
C24	0.6857 (3)	0.27537 (14)	0.7075 (3)	0.0378 (8)	
C25	0.6173 (3)	0.31365 (15)	0.7525 (3)	0.0422 (9)	
H25	0.618742	0.311707	0.822587	0.051*	
C26	0.5467 (3)	0.35490 (15)	0.6916 (3)	0.0439 (9)	
H26	0.501084	0.381014	0.721785	0.053*	
C27	0.4575 (3)	0.40162 (15)	0.5219 (3)	0.0393 (8)	
C28	1.0539 (3)	0.35016 (13)	0.5371 (2)	0.0332 (7)	
C29	1.0084 (3)	0.32960 (15)	0.6197 (3)	0.0435 (9)	
H29	0.927288	0.339430	0.625776	0.052*	
C30	1.0812 (3)	0.29473 (14)	0.6934 (3)	0.0436 (9)	
H30	1.050319	0.282046	0.749431	0.052*	
C31	1.2002 (3)	0.27904 (13)	0.6825 (3)	0.0365 (8)	
C32	1.2455 (3)	0.29675 (14)	0.5995 (3)	0.0411 (8)	
H32	1.324481	0.284729	0.591371	0.049*	
C33	1.1730 (3)	0.33275 (14)	0.5275 (3)	0.0413 (8)	
H33	1.204660	0.345427	0.471774	0.050*	
C34	0.9773 (3)	0.39127 (14)	0.4615 (3)	0.0419 (9)	
I1	0.78561 (3)	0.21027 (2)	0.79917 (2)	0.05702 (10)	
I2	1.31203 (3)	0.22704 (2)	0.79616 (2)	0.05455 (10)	
N1	0.6722 (2)	0.53483 (12)	0.1613 (2)	0.0367 (7)	
N2	0.7303 (3)	0.47858 (13)	0.3568 (2)	0.0450 (8)	
H21N	0.684 (3)	0.4978 (14)	0.392 (2)	0.054*	
H22N	0.771 (3)	0.4508 (12)	0.397 (2)	0.054*	
N3	0.1911 (2)	0.53973 (11)	0.1718 (2)	0.0367 (7)	
N4	0.2388 (3)	0.45488 (14)	0.3322 (2)	0.0460 (8)	
H41N	0.300 (3)	0.4353 (14)	0.370 (2)	0.055*	
H42N	0.170 (2)	0.4466 (15)	0.350 (3)	0.055*	
O1	0.4122 (2)	0.44040 (11)	0.56598 (19)	0.0571 (7)	
O2	0.4359 (2)	0.39436 (11)	0.42592 (19)	0.0561 (7)	
O3	1.0238 (2)	0.41261 (12)	0.3931 (2)	0.0644 (8)	
O4	0.8680 (2)	0.40136 (11)	0.4733 (2)	0.0645 (8)	

### 4-Phenylpiperazin-1-ium 4-iodobenzoate (4) Atomic displacement parameters (Å^2^)

**Table:**

	*U*^11^	*U*^22^	*U*^33^	*U*^12^	*U*^13^	*U*^23^
C1	0.043 (2)	0.033 (2)	0.0308 (18)	−0.0001 (16)	0.0052 (16)	−0.0052 (15)
C2	0.039 (2)	0.046 (2)	0.041 (2)	−0.0046 (17)	0.0016 (17)	−0.0035 (17)
C3	0.051 (2)	0.056 (3)	0.047 (2)	0.003 (2)	−0.0029 (19)	0.004 (2)
C4	0.071 (3)	0.055 (3)	0.046 (2)	0.009 (2)	0.004 (2)	0.0126 (19)
C5	0.059 (3)	0.062 (3)	0.055 (3)	−0.001 (2)	0.018 (2)	0.016 (2)
C6	0.045 (2)	0.053 (2)	0.047 (2)	0.0058 (19)	0.0084 (19)	0.0039 (19)
C7	0.039 (2)	0.040 (2)	0.038 (2)	−0.0020 (16)	0.0033 (16)	−0.0034 (16)
C8	0.049 (2)	0.043 (2)	0.047 (2)	−0.0008 (18)	0.0116 (19)	0.0044 (18)
C9	0.051 (2)	0.045 (2)	0.047 (2)	−0.0004 (19)	−0.0076 (19)	−0.0001 (18)
C10	0.045 (2)	0.040 (2)	0.041 (2)	−0.0041 (17)	−0.0035 (17)	−0.0029 (17)
C11	0.0287 (18)	0.034 (2)	0.045 (2)	−0.0064 (15)	−0.0052 (16)	0.0021 (16)
C12	0.052 (2)	0.041 (2)	0.052 (2)	0.0060 (18)	−0.0013 (19)	0.0017 (18)
C13	0.055 (3)	0.038 (2)	0.079 (3)	0.0053 (19)	−0.007 (2)	0.008 (2)
C14	0.055 (3)	0.056 (3)	0.063 (3)	−0.005 (2)	−0.006 (2)	0.025 (2)
C15	0.047 (2)	0.055 (3)	0.050 (2)	−0.011 (2)	0.0033 (19)	0.015 (2)
C16	0.038 (2)	0.042 (2)	0.048 (2)	−0.0036 (16)	0.0020 (18)	0.0056 (17)
C17	0.068 (3)	0.036 (2)	0.047 (2)	−0.0018 (19)	−0.011 (2)	0.0027 (17)
C18	0.069 (3)	0.035 (2)	0.045 (2)	0.0006 (18)	−0.001 (2)	0.0045 (17)
C19	0.047 (2)	0.042 (2)	0.040 (2)	0.0026 (17)	0.0040 (18)	0.0009 (17)
C20	0.054 (2)	0.048 (2)	0.037 (2)	0.0068 (19)	−0.0002 (18)	0.0008 (17)
C21	0.0347 (18)	0.0315 (19)	0.0323 (18)	−0.0013 (15)	0.0060 (15)	−0.0008 (15)
C22	0.042 (2)	0.045 (2)	0.0341 (19)	0.0010 (17)	0.0088 (16)	−0.0007 (16)
C23	0.050 (2)	0.035 (2)	0.043 (2)	0.0115 (16)	0.0121 (18)	−0.0007 (16)
C24	0.0397 (19)	0.0293 (19)	0.0396 (19)	−0.0034 (15)	−0.0018 (16)	0.0035 (15)
C25	0.051 (2)	0.045 (2)	0.0300 (18)	0.0019 (18)	0.0073 (17)	−0.0025 (16)
C26	0.049 (2)	0.043 (2)	0.040 (2)	0.0125 (17)	0.0076 (17)	−0.0043 (17)
C27	0.037 (2)	0.038 (2)	0.041 (2)	0.0022 (16)	0.0039 (17)	0.0011 (16)
C28	0.0345 (18)	0.0255 (18)	0.0378 (19)	−0.0007 (15)	0.0040 (16)	−0.0040 (14)
C29	0.040 (2)	0.038 (2)	0.057 (2)	0.0005 (17)	0.0202 (18)	−0.0016 (18)
C30	0.056 (2)	0.034 (2)	0.046 (2)	0.0014 (18)	0.0232 (19)	0.0025 (17)
C31	0.049 (2)	0.0234 (18)	0.0368 (19)	−0.0023 (15)	0.0091 (17)	−0.0032 (14)
C32	0.0384 (19)	0.041 (2)	0.045 (2)	0.0062 (16)	0.0121 (17)	0.0039 (17)
C33	0.047 (2)	0.040 (2)	0.039 (2)	0.0017 (17)	0.0147 (17)	0.0038 (16)
C34	0.043 (2)	0.032 (2)	0.048 (2)	−0.0049 (16)	0.0022 (19)	−0.0041 (17)
I1	0.0741 (2)	0.03693 (15)	0.05275 (17)	0.00927 (12)	−0.00227 (14)	0.00844 (12)
I2	0.07106 (19)	0.04819 (17)	0.04096 (15)	0.00624 (13)	0.00451 (13)	0.00842 (11)
N1	0.0384 (16)	0.0353 (17)	0.0340 (15)	−0.0029 (13)	0.0024 (13)	−0.0008 (13)
N2	0.055 (2)	0.039 (2)	0.0394 (18)	0.0156 (15)	0.0074 (16)	0.0050 (14)
N3	0.0403 (16)	0.0308 (16)	0.0344 (15)	−0.0016 (13)	−0.0020 (13)	−0.0011 (12)
N4	0.0415 (19)	0.050 (2)	0.0430 (19)	0.0044 (16)	0.0021 (16)	0.0144 (15)
O1	0.0706 (18)	0.0487 (17)	0.0495 (16)	0.0268 (14)	0.0076 (14)	−0.0019 (13)
O2	0.0652 (18)	0.0619 (18)	0.0362 (15)	0.0221 (14)	0.0001 (13)	0.0015 (13)
O3	0.0583 (18)	0.074 (2)	0.0632 (18)	0.0164 (15)	0.0181 (15)	0.0297 (16)
O4	0.0441 (16)	0.0659 (19)	0.085 (2)	0.0157 (14)	0.0169 (15)	0.0297 (16)

### 4-Phenylpiperazin-1-ium 4-iodobenzoate (4) Geometric parameters (Å, º)

**Table:**

C1—C2	1.380 (4)	C18—N4	1.472 (4)
C1—C6	1.393 (5)	C18—H18A	0.9700
C1—N1	1.422 (4)	C18—H18B	0.9700
C2—C3	1.383 (5)	C19—N3	1.450 (4)
C2—H2	0.9300	C19—C20	1.506 (5)
C3—C4	1.376 (5)	C19—H19A	0.9700
C3—H3	0.9300	C19—H19B	0.9700
C4—C5	1.377 (5)	C20—N4	1.479 (5)
C4—H4	0.9300	C20—H20A	0.9700
C5—C6	1.367 (5)	C20—H20B	0.9700
C5—H5	0.9300	C21—C26	1.377 (4)
C6—H6	0.9300	C21—C22	1.382 (4)
C7—N1	1.458 (4)	C21—C27	1.509 (4)
C7—C8	1.514 (4)	C22—C23	1.384 (4)
C7—H7A	0.9700	C22—H22	0.9300
C7—H7B	0.9700	C23—C24	1.378 (4)
C8—N2	1.485 (4)	C23—H23	0.9300
C8—H8A	0.9700	C24—C25	1.380 (5)
C8—H8B	0.9700	C24—I1	2.101 (3)
C9—N2	1.465 (5)	C25—C26	1.381 (4)
C9—C10	1.503 (5)	C25—H25	0.9300
C9—H9A	0.9700	C26—H26	0.9300
C9—H9B	0.9700	C27—O1	1.238 (4)
C10—N1	1.477 (4)	C27—O2	1.258 (4)
C10—H10A	0.9700	C28—C29	1.385 (4)
C10—H10B	0.9700	C28—C33	1.387 (4)
C11—C12	1.384 (5)	C28—C34	1.506 (5)
C11—C16	1.403 (5)	C29—C30	1.384 (5)
C11—N3	1.415 (4)	C29—H29	0.9300
C12—C13	1.394 (5)	C30—C31	1.380 (5)
C12—H12	0.9300	C30—H30	0.9300
C13—C14	1.378 (6)	C31—C32	1.366 (4)
C13—H13	0.9300	C31—I2	2.109 (3)
C14—C15	1.358 (5)	C32—C33	1.386 (4)
C14—H14	0.9300	C32—H32	0.9300
C15—C16	1.381 (5)	C33—H33	0.9300
C15—H15	0.9300	C34—O3	1.237 (4)
C16—H16	0.9300	C34—O4	1.253 (4)
C17—N3	1.463 (4)	N2—H21N	0.877 (18)
C17—C18	1.503 (5)	N2—H22N	0.897 (18)
C17—H17A	0.9700	N4—H41N	0.869 (18)
C17—H17B	0.9700	N4—H42N	0.851 (18)
			
C2—C1—C6	117.7 (3)	N3—C19—C20	110.5 (3)
C2—C1—N1	124.0 (3)	N3—C19—H19A	109.5
C6—C1—N1	118.3 (3)	C20—C19—H19A	109.5
C1—C2—C3	120.8 (3)	N3—C19—H19B	109.5
C1—C2—H2	119.6	C20—C19—H19B	109.5
C3—C2—H2	119.6	H19A—C19—H19B	108.1
C4—C3—C2	120.6 (4)	N4—C20—C19	112.5 (3)
C4—C3—H3	119.7	N4—C20—H20A	109.1
C2—C3—H3	119.7	C19—C20—H20A	109.1
C3—C4—C5	119.0 (4)	N4—C20—H20B	109.1
C3—C4—H4	120.5	C19—C20—H20B	109.1
C5—C4—H4	120.5	H20A—C20—H20B	107.8
C6—C5—C4	120.4 (4)	C26—C21—C22	118.3 (3)
C6—C5—H5	119.8	C26—C21—C27	120.6 (3)
C4—C5—H5	119.8	C22—C21—C27	121.0 (3)
C5—C6—C1	121.5 (3)	C21—C22—C23	120.9 (3)
C5—C6—H6	119.3	C21—C22—H22	119.5
C1—C6—H6	119.3	C23—C22—H22	119.5
N1—C7—C8	110.7 (3)	C24—C23—C22	119.5 (3)
N1—C7—H7A	109.5	C24—C23—H23	120.3
C8—C7—H7A	109.5	C22—C23—H23	120.3
N1—C7—H7B	109.5	C23—C24—C25	120.6 (3)
C8—C7—H7B	109.5	C23—C24—I1	120.8 (2)
H7A—C7—H7B	108.1	C25—C24—I1	118.5 (3)
N2—C8—C7	110.1 (3)	C24—C25—C26	118.8 (3)
N2—C8—H8A	109.6	C24—C25—H25	120.6
C7—C8—H8A	109.6	C26—C25—H25	120.6
N2—C8—H8B	109.6	C21—C26—C25	121.8 (3)
C7—C8—H8B	109.6	C21—C26—H26	119.1
H8A—C8—H8B	108.2	C25—C26—H26	119.1
N2—C9—C10	111.1 (3)	O1—C27—O2	125.0 (3)
N2—C9—H9A	109.4	O1—C27—C21	118.0 (3)
C10—C9—H9A	109.4	O2—C27—C21	116.9 (3)
N2—C9—H9B	109.4	C29—C28—C33	118.1 (3)
C10—C9—H9B	109.4	C29—C28—C34	120.8 (3)
H9A—C9—H9B	108.0	C33—C28—C34	121.1 (3)
N1—C10—C9	110.6 (3)	C30—C29—C28	121.3 (3)
N1—C10—H10A	109.5	C30—C29—H29	119.3
C9—C10—H10A	109.5	C28—C29—H29	119.3
N1—C10—H10B	109.5	C31—C30—C29	119.1 (3)
C9—C10—H10B	109.5	C31—C30—H30	120.5
H10A—C10—H10B	108.1	C29—C30—H30	120.5
C12—C11—C16	117.8 (3)	C32—C31—C30	120.8 (3)
C12—C11—N3	123.1 (3)	C32—C31—I2	120.2 (2)
C16—C11—N3	119.1 (3)	C30—C31—I2	119.0 (3)
C11—C12—C13	120.8 (4)	C31—C32—C33	119.5 (3)
C11—C12—H12	119.6	C31—C32—H32	120.2
C13—C12—H12	119.6	C33—C32—H32	120.2
C14—C13—C12	120.1 (4)	C32—C33—C28	121.1 (3)
C14—C13—H13	119.9	C32—C33—H33	119.5
C12—C13—H13	119.9	C28—C33—H33	119.5
C15—C14—C13	119.7 (4)	O3—C34—O4	124.4 (3)
C15—C14—H14	120.1	O3—C34—C28	119.7 (3)
C13—C14—H14	120.1	O4—C34—C28	115.9 (3)
C14—C15—C16	120.9 (4)	C1—N1—C7	115.5 (3)
C14—C15—H15	119.5	C1—N1—C10	112.3 (3)
C16—C15—H15	119.5	C7—N1—C10	111.1 (3)
C15—C16—C11	120.6 (4)	C9—N2—C8	110.8 (3)
C15—C16—H16	119.7	C9—N2—H21N	113 (2)
C11—C16—H16	119.7	C8—N2—H21N	106 (2)
N3—C17—C18	110.5 (3)	C9—N2—H22N	108 (2)
N3—C17—H17A	109.5	C8—N2—H22N	110 (2)
C18—C17—H17A	109.5	H21N—N2—H22N	109 (3)
N3—C17—H17B	109.5	C11—N3—C19	116.9 (3)
C18—C17—H17B	109.5	C11—N3—C17	114.5 (3)
H17A—C17—H17B	108.1	C19—N3—C17	109.1 (3)
N4—C18—C17	112.4 (3)	C18—N4—C20	111.3 (3)
N4—C18—H18A	109.1	C18—N4—H41N	108 (2)
C17—C18—H18A	109.1	C20—N4—H41N	108 (2)
N4—C18—H18B	109.1	C18—N4—H42N	112 (3)
C17—C18—H18B	109.1	C20—N4—H42N	109 (3)
H18A—C18—H18B	107.9	H41N—N4—H42N	109 (3)
			
C6—C1—C2—C3	−0.2 (5)	C33—C28—C29—C30	2.8 (5)
N1—C1—C2—C3	−179.4 (3)	C34—C28—C29—C30	−175.8 (3)
C1—C2—C3—C4	−0.6 (6)	C28—C29—C30—C31	−1.7 (5)
C2—C3—C4—C5	0.6 (6)	C29—C30—C31—C32	−1.0 (5)
C3—C4—C5—C6	0.2 (6)	C29—C30—C31—I2	179.0 (3)
C4—C5—C6—C1	−1.1 (6)	C30—C31—C32—C33	2.6 (5)
C2—C1—C6—C5	1.1 (5)	I2—C31—C32—C33	−177.5 (3)
N1—C1—C6—C5	−179.7 (3)	C31—C32—C33—C28	−1.4 (5)
N1—C7—C8—N2	56.9 (4)	C29—C28—C33—C32	−1.3 (5)
N2—C9—C10—N1	−56.4 (4)	C34—C28—C33—C32	177.3 (3)
C16—C11—C12—C13	−0.5 (5)	C29—C28—C34—O3	174.3 (3)
N3—C11—C12—C13	177.5 (3)	C33—C28—C34—O3	−4.3 (5)
C11—C12—C13—C14	1.1 (6)	C29—C28—C34—O4	−5.8 (5)
C12—C13—C14—C15	−0.8 (6)	C33—C28—C34—O4	175.6 (3)
C13—C14—C15—C16	−0.2 (6)	C2—C1—N1—C7	7.2 (4)
C14—C15—C16—C11	0.8 (5)	C6—C1—N1—C7	−172.0 (3)
C12—C11—C16—C15	−0.5 (5)	C2—C1—N1—C10	−121.7 (3)
N3—C11—C16—C15	−178.5 (3)	C6—C1—N1—C10	59.2 (4)
N3—C17—C18—N4	56.1 (4)	C8—C7—N1—C1	173.3 (3)
N3—C19—C20—N4	−55.9 (4)	C8—C7—N1—C10	−57.3 (3)
C26—C21—C22—C23	0.4 (5)	C9—C10—N1—C1	−172.1 (3)
C27—C21—C22—C23	−176.6 (3)	C9—C10—N1—C7	56.8 (4)
C21—C22—C23—C24	0.3 (5)	C10—C9—N2—C8	56.7 (4)
C22—C23—C24—C25	−0.6 (5)	C7—C8—N2—C9	−56.5 (4)
C22—C23—C24—I1	176.5 (2)	C12—C11—N3—C19	−3.6 (5)
C23—C24—C25—C26	0.2 (5)	C16—C11—N3—C19	174.3 (3)
I1—C24—C25—C26	−177.0 (3)	C12—C11—N3—C17	125.8 (4)
C22—C21—C26—C25	−0.9 (5)	C16—C11—N3—C17	−56.2 (4)
C27—C21—C26—C25	176.2 (3)	C20—C19—N3—C11	−167.4 (3)
C24—C25—C26—C21	0.6 (5)	C20—C19—N3—C17	60.7 (4)
C26—C21—C27—O1	13.6 (5)	C18—C17—N3—C11	165.9 (3)
C22—C21—C27—O1	−169.4 (3)	C18—C17—N3—C19	−60.9 (4)
C26—C21—C27—O2	−164.6 (3)	C17—C18—N4—C20	−50.0 (4)
C22—C21—C27—O2	12.4 (5)	C19—C20—N4—C18	49.9 (4)

### 4-Phenylpiperazin-1-ium 4-iodobenzoate (4) Hydrogen-bond geometry (Å, º)

**Table:**

*D*—H···*A*	*D*—H	H···*A*	*D*···*A*	*D*—H···*A*
C9—H9*A*···O3	0.97	2.63	3.218 (4)	119
N2—H21*N*···O1^i^	0.88 (2)	1.94 (2)	2.780 (4)	160 (3)
N2—H22*N*···O4	0.90 (2)	1.74 (2)	2.627 (4)	173 (3)
N4—H41*N*···O1	0.87 (2)	2.63 (3)	3.285 (4)	133 (3)
N4—H41*N*···O2	0.87 (2)	1.78 (2)	2.643 (4)	169 (3)
N4—H42*N*···O3^ii^	0.85 (2)	1.97 (2)	2.809 (4)	169 (4)

Symmetry codes: (i) −*x*+1, −*y*+1, −*z*+1; (ii) *x*−1, *y*, *z*.

### 4-Phenylpiperazin-1-ium 4-nitrobenzoate (5) Crystal data

**Table:**

C_10_H_15_N_2_^+^·C_7_H_4_NO_4_^−^	*F*(000) = 696
*M**_r_* = 329.35	*D*_x_ = 1.343 Mg m^−^^3^
Monoclinic, *P*2_1_/*c*	Mo *K*α radiation, λ = 0.71073 Å
*a* = 13.0683 (9) Å	Cell parameters from 4084 reflections
*b* = 15.7868 (9) Å	θ = 2.6–27.9°
*c* = 7.9255 (5) Å	µ = 0.10 mm^−^^1^
β = 95.137 (6)°	*T* = 293 K
*V* = 1628.52 (18) Å^3^	Prism, yellow
*Z* = 4	0.48 × 0.44 × 0.16 mm

### 4-Phenylpiperazin-1-ium 4-nitrobenzoate (5) Data collection

**Table:**

Oxford Diffraction Xcalibur with Sapphire CCD diffractometer	2088 reflections with *I* > 2σ(*I*)
Radiation source: Enhance (Mo) X-ray Source	*R*_int_ = 0.035
Rotation method data acquisition using ω scans.	θ_max_ = 28.0°, θ_min_ = 2.9°
Absorption correction: multi-scan (CrysalisRED; Oxford Diffraction, 2007)	*h* = −12→17
*T*_min_ = 0.790, *T*_max_ = 1.000	*k* = −19→20
11699 measured reflections	*l* = −10→10
3587 independent reflections	

### 4-Phenylpiperazin-1-ium 4-nitrobenzoate (5) Refinement

**Table:**

Refinement on *F*^2^	Primary atom site location: dual
Least-squares matrix: full	Secondary atom site location: difference Fourier map
*R*[*F*^2^ > 2σ(*F*^2^)] = 0.054	Hydrogen site location: mixed
*wR*(*F*^2^) = 0.117	H atoms treated by a mixture of independent and constrained refinement
*S* = 1.10	*w* = 1/[σ^2^(*F*_o_^2^) + (0.0406*P*)^2^ + 0.2296*P*] where *P* = (*F*_o_^2^ + 2*F*_c_^2^)/3
3587 reflections	(Δ/σ)_max_ < 0.001
251 parameters	Δρ_max_ = 0.15 e Å^−^^3^
83 restraints	Δρ_min_ = −0.13 e Å^−^^3^

### 4-Phenylpiperazin-1-ium 4-nitrobenzoate (5) Special details

**Table:**

Geometry. All esds (except the esd in the dihedral angle between two l.s. planes) are estimated using the full covariance matrix. The cell esds are taken into account individually in the estimation of esds in distances, angles and torsion angles; correlations between esds in cell parameters are only used when they are defined by crystal symmetry. An approximate (isotropic) treatment of cell esds is used for estimating esds involving l.s. planes.

### 4-Phenylpiperazin-1-ium 4-nitrobenzoate (5) Fractional atomic coordinates and isotropic or equivalent isotropic displacement parameters (Å^2^)

**Table:**

	*x*	*y*	*z*	*U*_iso_*/*U*_eq_	Occ. (<1)
N1	0.31713 (11)	0.63737 (9)	0.06853 (18)	0.0443 (4)	
N2	0.42384 (12)	0.61812 (10)	0.3978 (2)	0.0497 (4)	
H21	0.4059 (14)	0.5640 (10)	0.412 (2)	0.060*	
H22	0.4650 (13)	0.6358 (11)	0.493 (2)	0.060*	
C1	0.25304 (14)	0.61655 (11)	−0.0794 (2)	0.0451 (5)	
C2	0.16112 (15)	0.66029 (13)	−0.1177 (3)	0.0592 (5)	
H2	0.141977	0.702437	−0.044776	0.071*	
C3	0.09843 (17)	0.64191 (16)	−0.2618 (3)	0.0711 (7)	
H3	0.037601	0.671965	−0.285079	0.085*	
C4	0.12406 (19)	0.58034 (18)	−0.3709 (3)	0.0786 (7)	
H4	0.080984	0.567936	−0.467562	0.094*	
C5	0.21412 (19)	0.53709 (16)	−0.3360 (3)	0.0769 (7)	
H5	0.232309	0.495042	−0.409926	0.092*	
C6	0.27853 (16)	0.55500 (13)	−0.1925 (2)	0.0603 (6)	
H6	0.339789	0.525251	−0.171661	0.072*	
C7	0.41360 (13)	0.59047 (11)	0.0927 (2)	0.0482 (5)	
H7A	0.450391	0.596351	−0.007625	0.058*	
H7B	0.398842	0.530823	0.107119	0.058*	
C8	0.48078 (14)	0.62161 (12)	0.2451 (2)	0.0546 (5)	
H8A	0.541877	0.586642	0.261820	0.066*	
H8B	0.502144	0.679404	0.226049	0.066*	
C9	0.33189 (14)	0.67211 (12)	0.3727 (2)	0.0551 (5)	
H9A	0.352164	0.730174	0.353462	0.066*	
H9B	0.294913	0.670810	0.473427	0.066*	
C10	0.26333 (14)	0.64092 (12)	0.2229 (2)	0.0525 (5)	
H10A	0.238174	0.584849	0.247440	0.063*	
H10B	0.204495	0.678299	0.204255	0.063*	
O1	0.54550 (10)	0.66915 (8)	0.67450 (17)	0.0659 (4)	
O2	0.62576 (10)	0.55694 (8)	0.58251 (17)	0.0596 (4)	
N3	0.9330 (8)	0.6415 (18)	1.2296 (16)	0.0790 (18)	0.62 (3)
O3	0.9183 (12)	0.6862 (8)	1.3520 (19)	0.108 (3)	0.62 (3)
O4	1.0096 (8)	0.5973 (11)	1.2186 (12)	0.095 (2)	0.62 (3)
N3A	0.9219 (15)	0.647 (3)	1.244 (3)	0.082 (3)	0.38 (3)
O3A	0.8898 (15)	0.6659 (14)	1.380 (2)	0.095 (3)	0.38 (3)
O4A	1.0127 (12)	0.6352 (18)	1.223 (2)	0.101 (3)	0.38 (3)
C11	0.70169 (13)	0.62778 (10)	0.8251 (2)	0.0422 (4)	
C12	0.68311 (14)	0.67242 (11)	0.9690 (2)	0.0505 (5)	
H12	0.620157	0.699232	0.974825	0.061*	
C13	0.75707 (16)	0.67767 (12)	1.1045 (3)	0.0591 (5)	
H13	0.744175	0.706602	1.202574	0.071*	
C14	0.84977 (16)	0.63922 (13)	1.0907 (3)	0.0567 (5)	
C15	0.87192 (15)	0.59666 (13)	0.9479 (3)	0.0603 (6)	
H15	0.936114	0.572184	0.940687	0.072*	
C16	0.79686 (14)	0.59106 (12)	0.8155 (2)	0.0523 (5)	
H16	0.810377	0.562065	0.717750	0.063*	
C17	0.61816 (14)	0.61734 (11)	0.6823 (2)	0.0465 (5)	

### 4-Phenylpiperazin-1-ium 4-nitrobenzoate (5) Atomic displacement parameters (Å^2^)

**Table:**

	*U*^11^	*U*^22^	*U*^33^	*U*^12^	*U*^13^	*U*^23^
N1	0.0417 (9)	0.0431 (8)	0.0468 (9)	0.0018 (7)	−0.0038 (7)	0.0015 (7)
N2	0.0525 (10)	0.0434 (9)	0.0505 (10)	0.0009 (7)	−0.0110 (8)	−0.0029 (8)
C1	0.0461 (11)	0.0442 (10)	0.0437 (11)	−0.0081 (8)	−0.0027 (8)	0.0094 (9)
C2	0.0514 (12)	0.0637 (13)	0.0606 (13)	−0.0023 (10)	−0.0057 (10)	0.0071 (11)
C3	0.0526 (14)	0.0923 (17)	0.0657 (16)	−0.0093 (12)	−0.0104 (11)	0.0187 (14)
C4	0.0733 (17)	0.112 (2)	0.0476 (14)	−0.0251 (15)	−0.0141 (12)	0.0100 (14)
C5	0.092 (2)	0.0928 (18)	0.0446 (13)	−0.0129 (14)	−0.0014 (12)	−0.0059 (12)
C6	0.0661 (14)	0.0663 (13)	0.0472 (12)	0.0008 (10)	−0.0018 (10)	0.0001 (11)
C7	0.0458 (11)	0.0493 (10)	0.0489 (12)	0.0022 (9)	0.0014 (8)	0.0022 (9)
C8	0.0457 (12)	0.0565 (12)	0.0603 (13)	0.0002 (9)	−0.0025 (10)	0.0042 (10)
C9	0.0561 (12)	0.0510 (11)	0.0562 (12)	0.0111 (9)	−0.0070 (9)	−0.0086 (10)
C10	0.0478 (12)	0.0564 (12)	0.0522 (12)	0.0093 (9)	−0.0026 (9)	−0.0065 (9)
O1	0.0597 (9)	0.0622 (8)	0.0710 (10)	0.0133 (7)	−0.0213 (7)	−0.0130 (7)
O2	0.0715 (10)	0.0475 (7)	0.0567 (9)	0.0020 (7)	−0.0117 (7)	−0.0087 (7)
N3	0.068 (4)	0.100 (5)	0.065 (3)	−0.018 (4)	−0.019 (3)	0.005 (3)
O3	0.106 (5)	0.126 (5)	0.084 (5)	−0.009 (4)	−0.036 (4)	−0.028 (4)
O4	0.056 (3)	0.140 (6)	0.084 (3)	0.000 (4)	−0.0213 (18)	0.014 (4)
N3A	0.064 (5)	0.105 (6)	0.071 (5)	−0.007 (5)	−0.024 (5)	0.007 (5)
O3A	0.091 (7)	0.124 (7)	0.066 (5)	−0.011 (5)	−0.025 (4)	−0.011 (5)
O4A	0.050 (4)	0.146 (8)	0.102 (5)	−0.018 (6)	−0.023 (3)	0.020 (6)
C11	0.0450 (11)	0.0361 (9)	0.0444 (11)	−0.0045 (8)	−0.0029 (8)	0.0032 (8)
C12	0.0491 (12)	0.0475 (11)	0.0538 (12)	0.0021 (9)	−0.0014 (9)	−0.0022 (9)
C13	0.0690 (15)	0.0557 (12)	0.0510 (12)	−0.0014 (11)	−0.0046 (10)	−0.0104 (10)
C14	0.0520 (13)	0.0599 (13)	0.0550 (13)	−0.0116 (10)	−0.0127 (10)	0.0058 (10)
C15	0.0414 (12)	0.0722 (14)	0.0664 (15)	0.0017 (10)	−0.0008 (10)	0.0060 (12)
C16	0.0500 (12)	0.0564 (12)	0.0500 (12)	0.0033 (10)	0.0016 (9)	−0.0033 (9)
C17	0.0498 (12)	0.0389 (10)	0.0492 (12)	−0.0040 (9)	−0.0044 (9)	0.0045 (9)

### 4-Phenylpiperazin-1-ium 4-nitrobenzoate (5) Geometric parameters (Å, º)

**Table:**

N1—C1	1.417 (2)	C9—H9A	0.9700
N1—C7	1.460 (2)	C9—H9B	0.9700
N1—C10	1.466 (2)	C10—H10A	0.9700
N2—C9	1.472 (2)	C10—H10B	0.9700
N2—C8	1.477 (3)	O1—C17	1.250 (2)
N2—H21	0.896 (14)	O2—C17	1.248 (2)
N2—H22	0.931 (14)	N3—O4	1.229 (10)
C1—C6	1.383 (3)	N3—O3	1.229 (9)
C1—C2	1.395 (3)	N3—C14	1.477 (8)
C2—C3	1.376 (3)	N3A—O4A	1.229 (10)
C2—H2	0.9300	N3A—O3A	1.229 (9)
C3—C4	1.363 (3)	N3A—C14	1.474 (14)
C3—H3	0.9300	C11—C16	1.380 (2)
C4—C5	1.367 (3)	C11—C12	1.381 (2)
C4—H4	0.9300	C11—C17	1.510 (2)
C5—C6	1.383 (3)	C12—C13	1.381 (3)
C5—H5	0.9300	C12—H12	0.9300
C6—H6	0.9300	C13—C14	1.368 (3)
C7—C8	1.510 (2)	C13—H13	0.9300
C7—H7A	0.9700	C14—C15	1.370 (3)
C7—H7B	0.9700	C15—C16	1.374 (3)
C8—H8A	0.9700	C15—H15	0.9300
C8—H8B	0.9700	C16—H16	0.9300
C9—C10	1.504 (2)		
			
C1—N1—C7	115.50 (14)	N2—C9—H9A	109.7
C1—N1—C10	114.03 (14)	C10—C9—H9A	109.7
C7—N1—C10	112.51 (13)	N2—C9—H9B	109.7
C9—N2—C8	109.43 (15)	C10—C9—H9B	109.7
C9—N2—H21	110.4 (12)	H9A—C9—H9B	108.2
C8—N2—H21	107.1 (13)	N1—C10—C9	112.01 (16)
C9—N2—H22	110.0 (11)	N1—C10—H10A	109.2
C8—N2—H22	110.9 (12)	C9—C10—H10A	109.2
H21—N2—H22	108.9 (16)	N1—C10—H10B	109.2
C6—C1—C2	117.37 (17)	C9—C10—H10B	109.2
C6—C1—N1	122.71 (16)	H10A—C10—H10B	107.9
C2—C1—N1	119.91 (17)	O4—N3—O3	124.7 (4)
C3—C2—C1	120.9 (2)	O4—N3—C14	118.9 (9)
C3—C2—H2	119.6	O3—N3—C14	116.4 (9)
C1—C2—H2	119.6	O4A—N3A—O3A	124.6 (5)
C4—C3—C2	121.0 (2)	O4A—N3A—C14	115.4 (12)
C4—C3—H3	119.5	O3A—N3A—C14	120.0 (12)
C2—C3—H3	119.5	C16—C11—C12	119.07 (16)
C3—C4—C5	118.9 (2)	C16—C11—C17	120.49 (17)
C3—C4—H4	120.5	C12—C11—C17	120.42 (17)
C5—C4—H4	120.5	C11—C12—C13	120.68 (18)
C4—C5—C6	120.9 (2)	C11—C12—H12	119.7
C4—C5—H5	119.5	C13—C12—H12	119.7
C6—C5—H5	119.5	C14—C13—C12	118.33 (19)
C5—C6—C1	120.9 (2)	C14—C13—H13	120.8
C5—C6—H6	119.6	C12—C13—H13	120.8
C1—C6—H6	119.6	C13—C14—C15	122.52 (18)
N1—C7—C8	111.92 (15)	C13—C14—N3A	113.8 (8)
N1—C7—H7A	109.2	C15—C14—N3A	123.7 (8)
C8—C7—H7A	109.2	C13—C14—N3	121.9 (5)
N1—C7—H7B	109.2	C15—C14—N3	115.6 (5)
C8—C7—H7B	109.2	C14—C15—C16	118.29 (19)
H7A—C7—H7B	107.9	C14—C15—H15	120.9
N2—C8—C7	110.09 (16)	C16—C15—H15	120.9
N2—C8—H8A	109.6	C15—C16—C11	121.07 (19)
C7—C8—H8A	109.6	C15—C16—H16	119.5
N2—C8—H8B	109.6	C11—C16—H16	119.5
C7—C8—H8B	109.6	O2—C17—O1	124.86 (16)
H8A—C8—H8B	108.2	O2—C17—C11	117.72 (16)
N2—C9—C10	109.78 (15)	O1—C17—C11	117.41 (17)
			
C7—N1—C1—C6	1.1 (2)	C11—C12—C13—C14	1.5 (3)
C10—N1—C1—C6	−131.49 (19)	C12—C13—C14—C15	0.6 (3)
C7—N1—C1—C2	−177.35 (16)	C12—C13—C14—N3A	−179 (2)
C10—N1—C1—C2	50.0 (2)	C12—C13—C14—N3	−179.8 (14)
C6—C1—C2—C3	0.6 (3)	O4A—N3A—C14—C13	−160 (3)
N1—C1—C2—C3	179.11 (18)	O3A—N3A—C14—C13	18 (5)
C1—C2—C3—C4	0.2 (3)	O4A—N3A—C14—C15	21 (5)
C2—C3—C4—C5	−0.6 (3)	O3A—N3A—C14—C15	−161 (3)
C3—C4—C5—C6	0.1 (4)	O4—N3—C14—C13	171.1 (17)
C4—C5—C6—C1	0.7 (3)	O3—N3—C14—C13	−7 (3)
C2—C1—C6—C5	−1.0 (3)	O4—N3—C14—C15	−9 (3)
N1—C1—C6—C5	−179.50 (18)	O3—N3—C14—C15	173.1 (18)
C1—N1—C7—C8	175.30 (15)	C13—C14—C15—C16	−1.5 (3)
C10—N1—C7—C8	−51.4 (2)	N3A—C14—C15—C16	177 (2)
C9—N2—C8—C7	−60.00 (19)	N3—C14—C15—C16	178.8 (13)
N1—C7—C8—N2	55.4 (2)	C14—C15—C16—C11	0.4 (3)
C8—N2—C9—C10	60.4 (2)	C12—C11—C16—C15	1.5 (3)
C1—N1—C10—C9	−173.98 (14)	C17—C11—C16—C15	−176.86 (17)
C7—N1—C10—C9	52.0 (2)	C16—C11—C17—O2	20.4 (3)
N2—C9—C10—N1	−56.4 (2)	C12—C11—C17—O2	−157.93 (17)
C16—C11—C12—C13	−2.5 (3)	C16—C11—C17—O1	−160.75 (18)
C17—C11—C12—C13	175.88 (17)	C12—C11—C17—O1	20.9 (3)

### 4-Phenylpiperazin-1-ium 4-nitrobenzoate (5) Hydrogen-bond geometry (Å, º)

**Table:**

*D*—H···*A*	*D*—H	H···*A*	*D*···*A*	*D*—H···*A*
N2—H21···O2^i^	0.90 (1)	1.96 (2)	2.846 (2)	173 (2)
N2—H22···O1	0.93 (1)	1.78 (2)	2.7135 (19)	179 (2)
N2—H22···O2	0.93 (1)	2.49 (2)	3.057 (2)	120 (1)
C8—H8*B*···O1^ii^	0.97	2.50	3.468 (2)	176
C10—H10*B*···O4*A*^iii^	0.97	2.61	3.276 (15)	126

Symmetry codes: (i) −*x*+1, −*y*+1, −*z*+1; (ii) *x*, −*y*+3/2, *z*−1/2; (iii) *x*−1, *y*, *z*−1.

### 4-Phenylpiperazin-1-ium 2-hydroxy-4,6-dinitrophenolate (6) Crystal data

**Table:**

C_10_H_15_N_2_^+^·C_7_H_3_N_2_O_7_^−^	*F*(000) = 816
*M**_r_* = 390.35	*D*_x_ = 1.444 Mg m^−^^3^
Monoclinic, *P*2_1_/*c*	Mo *K*α radiation, λ = 0.71073 Å
*a* = 7.779 (3) Å	Cell parameters from 1200 reflections
*b* = 7.411 (3) Å	θ = 2.6–27.9°
*c* = 31.357 (9) Å	µ = 0.11 mm^−^^1^
β = 96.82 (3)°	*T* = 293 K
*V* = 1794.9 (11) Å^3^	Prism, yellow
*Z* = 4	0.20 × 0.18 × 0.12 mm

### 4-Phenylpiperazin-1-ium 2-hydroxy-4,6-dinitrophenolate (6) Data collection

**Table:**

Oxford Diffraction Xcalibur with Sapphire CCD diffractometer	1590 reflections with *I* > 2σ(*I*)
Radiation source: Enhance (Mo) X-ray Source	*R*_int_ = 0.055
Rotation method data acquisition using ω scans.	θ_max_ = 28.0°, θ_min_ = 2.6°
Absorption correction: multi-scan (CrysalisRED; Oxford Diffraction, 2007)	*h* = −9→9
*T*_min_ = 0.959, *T*_max_ = 1.000	*k* = −9→9
7737 measured reflections	*l* = −40→26
3882 independent reflections	

### 4-Phenylpiperazin-1-ium 2-hydroxy-4,6-dinitrophenolate (6) Refinement

**Table:**

Refinement on *F*^2^	Primary atom site location: dual
Least-squares matrix: full	Secondary atom site location: difference Fourier map
*R*[*F*^2^ > 2σ(*F*^2^)] = 0.085	Hydrogen site location: mixed
*wR*(*F*^2^) = 0.155	H atoms treated by a mixture of independent and constrained refinement
*S* = 1.03	*w* = 1/[σ^2^(*F*_o_^2^) + (0.0396*P*)^2^ + 0.7929*P*] where *P* = (*F*_o_^2^ + 2*F*_c_^2^)/3
3882 reflections	(Δ/σ)_max_ < 0.001
321 parameters	Δρ_max_ = 0.18 e Å^−^^3^
288 restraints	Δρ_min_ = −0.20 e Å^−^^3^

### 4-Phenylpiperazin-1-ium 2-hydroxy-4,6-dinitrophenolate (6) Special details

**Table:**

Geometry. All esds (except the esd in the dihedral angle between two l.s. planes) are estimated using the full covariance matrix. The cell esds are taken into account individually in the estimation of esds in distances, angles and torsion angles; correlations between esds in cell parameters are only used when they are defined by crystal symmetry. An approximate (isotropic) treatment of cell esds is used for estimating esds involving l.s. planes.

### 4-Phenylpiperazin-1-ium 2-hydroxy-4,6-dinitrophenolate (6) Fractional atomic coordinates and isotropic or equivalent isotropic displacement parameters (Å^2^)

**Table:**

	*x*	*y*	*z*	*U*_iso_*/*U*_eq_	Occ. (<1)
O1	0.2174 (3)	0.5690 (4)	0.40623 (8)	0.0678 (9)	
O2	0.3815 (3)	0.5542 (4)	0.46760 (8)	0.0644 (9)	
H2O	0.372 (5)	0.581 (6)	0.4933 (7)	0.077*	
O3	0.2737 (3)	0.6604 (4)	0.53481 (7)	0.0538 (8)	
N3	0.0028 (7)	0.8237 (11)	0.57913 (17)	0.0504 (16)	0.690 (11)
O4	0.1059 (7)	0.7315 (9)	0.60356 (18)	0.069 (2)	0.690 (11)
O5	−0.0948 (7)	0.9353 (9)	0.5930 (2)	0.0602 (19)	0.690 (11)
N3A	−0.0015 (11)	0.825 (2)	0.5769 (3)	0.053 (3)	0.310 (11)
O4A	0.1438 (10)	0.818 (2)	0.5983 (4)	0.065 (4)	0.310 (11)
O5A	−0.1263 (11)	0.898 (2)	0.5910 (5)	0.053 (4)	0.310 (11)
O6	−0.4591 (3)	0.9346 (4)	0.45916 (8)	0.0725 (9)	
O7	−0.3746 (3)	0.8095 (4)	0.40327 (8)	0.0738 (9)	
N4	−0.3514 (4)	0.8515 (5)	0.44113 (10)	0.0517 (9)	
C11	0.0978 (4)	0.6775 (5)	0.46853 (11)	0.0390 (9)	
C12	0.1281 (4)	0.7048 (5)	0.51389 (11)	0.0418 (9)	
C13	−0.0142 (4)	0.7862 (5)	0.53288 (10)	0.0400 (9)	
C14	−0.1672 (4)	0.8346 (5)	0.50932 (11)	0.0398 (9)	
H14	−0.254887	0.888430	0.522579	0.048*	
C15	−0.1890 (4)	0.8024 (5)	0.46600 (11)	0.0393 (9)	
C16	−0.0579 (4)	0.7232 (5)	0.44552 (11)	0.0413 (10)	
H16	−0.075920	0.701222	0.416128	0.050*	
C17	0.2359 (5)	0.5974 (5)	0.44453 (12)	0.0477 (10)	
N1	0.6156 (4)	0.6931 (4)	0.70321 (9)	0.0467 (8)	
N2	0.4692 (4)	0.6095 (6)	0.61759 (11)	0.0666 (11)	
H21	0.387 (4)	0.620 (5)	0.5958 (9)	0.080*	
H22	0.548 (4)	0.538 (4)	0.6091 (11)	0.080*	
C1	0.7285 (5)	0.6958 (7)	0.74341 (14)	0.0502 (14)	0.687 (10)
C2	0.8534 (7)	0.8298 (8)	0.75105 (14)	0.0631 (17)	0.687 (10)
H2A	0.863427	0.918478	0.730495	0.076*	0.687 (10)
C3	0.9631 (6)	0.8314 (9)	0.78940 (16)	0.0671 (17)	0.687 (10)
H3A	1.046662	0.921115	0.794514	0.081*	0.687 (10)
C4	0.9481 (6)	0.6989 (9)	0.82012 (14)	0.0613 (17)	0.687 (10)
H4A	1.021562	0.699993	0.845781	0.074*	0.687 (10)
C5	0.8233 (7)	0.5649 (7)	0.81248 (16)	0.0652 (17)	0.687 (10)
H5A	0.813228	0.476232	0.833028	0.078*	0.687 (10)
C6	0.7135 (7)	0.5633 (7)	0.77412 (17)	0.0585 (16)	0.687 (10)
H6A	0.629992	0.473592	0.769009	0.070*	0.687 (10)
C1A	0.7263 (12)	0.7159 (17)	0.7414 (3)	0.054 (2)	0.313 (10)
C2A	0.8039 (15)	0.8840 (16)	0.7482 (3)	0.062 (2)	0.313 (10)
H2AA	0.782198	0.974477	0.727712	0.075*	0.313 (10)
C3A	0.9139 (16)	0.9170 (17)	0.7856 (4)	0.066 (2)	0.313 (10)
H3AA	0.965778	1.029454	0.790219	0.080*	0.313 (10)
C4A	0.9463 (13)	0.782 (2)	0.8163 (3)	0.064 (2)	0.313 (10)
H4AA	1.019913	0.803779	0.841325	0.076*	0.313 (10)
C5A	0.8688 (16)	0.6136 (17)	0.8094 (4)	0.059 (2)	0.313 (10)
H5AA	0.890467	0.523125	0.829924	0.070*	0.313 (10)
C6A	0.7588 (16)	0.5806 (15)	0.7720 (4)	0.059 (2)	0.313 (10)
H6AA	0.706886	0.468144	0.767417	0.071*	0.313 (10)
C7	0.5365 (5)	0.5189 (6)	0.69241 (12)	0.0624 (12)	
H7A	0.488024	0.471054	0.717189	0.075*	
H7B	0.624264	0.435314	0.685038	0.075*	
C8	0.3961 (5)	0.5342 (6)	0.65535 (12)	0.0674 (13)	
H8A	0.346810	0.416157	0.648371	0.081*	
H8B	0.304749	0.612321	0.663122	0.081*	
C9	0.5459 (5)	0.7883 (6)	0.62835 (12)	0.0652 (13)	
H9A	0.456601	0.870777	0.635373	0.078*	
H9B	0.595482	0.836462	0.603764	0.078*	
C10	0.6842 (5)	0.7725 (6)	0.66592 (12)	0.0645 (13)	
H10A	0.777785	0.697905	0.657962	0.077*	
H10B	0.730882	0.891256	0.673425	0.077*	

### 4-Phenylpiperazin-1-ium 2-hydroxy-4,6-dinitrophenolate (6) Atomic displacement parameters (Å^2^)

**Table:**

	*U*^11^	*U*^22^	*U*^33^	*U*^12^	*U*^13^	*U*^23^
O1	0.0641 (18)	0.097 (2)	0.0424 (17)	0.0242 (16)	0.0060 (13)	−0.0135 (18)
O2	0.0490 (17)	0.099 (2)	0.0463 (17)	0.0219 (16)	0.0089 (14)	0.0024 (19)
O3	0.0412 (15)	0.077 (2)	0.0425 (16)	0.0074 (14)	0.0000 (12)	0.0002 (15)
N3	0.047 (3)	0.068 (4)	0.037 (3)	−0.003 (3)	0.006 (3)	−0.006 (3)
O4	0.062 (3)	0.104 (5)	0.040 (3)	0.015 (3)	0.000 (2)	0.005 (3)
O5	0.059 (3)	0.070 (4)	0.053 (3)	−0.002 (3)	0.015 (3)	−0.018 (3)
N3A	0.045 (5)	0.072 (6)	0.042 (6)	−0.001 (5)	0.003 (5)	−0.006 (6)
O4A	0.053 (5)	0.093 (8)	0.045 (6)	0.009 (6)	−0.008 (5)	−0.005 (6)
O5A	0.049 (5)	0.078 (8)	0.032 (6)	−0.002 (5)	0.006 (5)	−0.021 (6)
O6	0.0612 (18)	0.096 (2)	0.0589 (19)	0.0335 (17)	0.0024 (14)	−0.0098 (18)
O7	0.074 (2)	0.103 (3)	0.0416 (17)	0.0259 (17)	−0.0089 (13)	−0.0162 (18)
N4	0.049 (2)	0.059 (2)	0.047 (2)	0.0105 (18)	0.0045 (16)	−0.004 (2)
C11	0.041 (2)	0.039 (2)	0.037 (2)	0.0007 (18)	0.0059 (17)	0.001 (2)
C12	0.040 (2)	0.044 (2)	0.042 (2)	−0.0043 (19)	0.0037 (17)	−0.001 (2)
C13	0.048 (2)	0.043 (3)	0.030 (2)	−0.0043 (19)	0.0082 (17)	−0.007 (2)
C14	0.041 (2)	0.036 (2)	0.044 (2)	0.0019 (18)	0.0103 (17)	−0.002 (2)
C15	0.038 (2)	0.043 (2)	0.037 (2)	0.0041 (18)	0.0025 (16)	−0.002 (2)
C16	0.051 (2)	0.040 (3)	0.032 (2)	0.0016 (19)	0.0056 (17)	−0.0001 (19)
C17	0.042 (2)	0.056 (3)	0.046 (3)	0.006 (2)	0.0065 (19)	0.001 (2)
N1	0.0485 (18)	0.055 (2)	0.0368 (18)	−0.0090 (17)	0.0049 (14)	0.0003 (18)
N2	0.054 (2)	0.103 (3)	0.042 (2)	0.022 (2)	−0.0011 (16)	−0.015 (2)
C1	0.047 (3)	0.067 (3)	0.038 (3)	−0.006 (3)	0.010 (2)	−0.006 (3)
C2	0.061 (3)	0.076 (4)	0.050 (3)	−0.016 (3)	−0.004 (3)	0.007 (3)
C3	0.065 (3)	0.080 (4)	0.055 (3)	−0.019 (3)	−0.001 (3)	0.008 (3)
C4	0.069 (3)	0.076 (4)	0.038 (3)	−0.015 (3)	0.003 (2)	−0.004 (3)
C5	0.064 (3)	0.089 (4)	0.043 (3)	−0.020 (3)	0.011 (3)	0.009 (3)
C6	0.057 (3)	0.081 (3)	0.038 (3)	−0.014 (3)	0.008 (2)	0.007 (3)
C1A	0.051 (4)	0.075 (4)	0.037 (4)	−0.011 (4)	0.011 (4)	0.003 (4)
C2A	0.058 (4)	0.081 (5)	0.049 (4)	−0.014 (4)	0.010 (4)	−0.004 (4)
C3A	0.062 (4)	0.084 (5)	0.053 (4)	−0.013 (4)	0.007 (4)	−0.001 (4)
C4A	0.064 (4)	0.084 (5)	0.043 (4)	−0.025 (4)	0.011 (4)	0.002 (4)
C5A	0.060 (4)	0.080 (4)	0.037 (4)	−0.023 (4)	0.008 (4)	0.009 (4)
C6A	0.057 (4)	0.079 (4)	0.041 (4)	−0.020 (4)	0.005 (4)	0.005 (4)
C7	0.073 (3)	0.067 (3)	0.046 (3)	−0.008 (2)	−0.001 (2)	0.003 (2)
C8	0.069 (3)	0.081 (4)	0.051 (3)	−0.013 (2)	0.002 (2)	−0.009 (3)
C9	0.059 (3)	0.086 (4)	0.050 (3)	−0.001 (3)	0.0037 (19)	0.018 (3)
C10	0.058 (3)	0.089 (4)	0.045 (2)	−0.013 (2)	0.0022 (19)	0.015 (3)

### 4-Phenylpiperazin-1-ium 2-hydroxy-4,6-dinitrophenolate (6) Geometric parameters (Å, º)

**Table:**

O1—C17	1.211 (4)	C1—C6	1.3900
O2—C17	1.309 (4)	C2—C3	1.3900
O2—H2O	0.841 (18)	C2—H2A	0.9300
O3—C12	1.283 (4)	C3—C4	1.3900
N3—O5	1.236 (4)	C3—H3A	0.9300
N3—O4	1.246 (5)	C4—C5	1.3900
N3—C13	1.467 (6)	C4—H4A	0.9300
N3A—O5A	1.237 (4)	C5—C6	1.3900
N3A—O4A	1.246 (5)	C5—H5A	0.9300
N3A—C13	1.401 (10)	C6—H6A	0.9300
O6—N4	1.230 (3)	C1A—C2A	1.3900
O7—N4	1.220 (3)	C1A—C6A	1.3900
N4—C15	1.450 (4)	C2A—C3A	1.3900
C11—C16	1.376 (4)	C2A—H2AA	0.9300
C11—C12	1.428 (4)	C3A—C4A	1.3900
C11—C17	1.505 (5)	C3A—H3AA	0.9300
C12—C13	1.449 (5)	C4A—C5A	1.3900
C13—C14	1.372 (4)	C4A—H4AA	0.9300
C14—C15	1.370 (4)	C5A—C6A	1.3900
C14—H14	0.9300	C5A—H5AA	0.9300
C15—C16	1.398 (4)	C6A—H6AA	0.9300
C16—H16	0.9300	C7—C8	1.501 (5)
N1—C1A	1.399 (9)	C7—H7A	0.9700
N1—C1	1.448 (5)	C7—H7B	0.9700
N1—C7	1.453 (5)	C8—H8A	0.9700
N1—C10	1.465 (4)	C8—H8B	0.9700
N2—C9	1.476 (5)	C9—C10	1.503 (5)
N2—C8	1.482 (5)	C9—H9A	0.9700
N2—H21	0.884 (18)	C9—H9B	0.9700
N2—H22	0.874 (18)	C10—H10A	0.9700
C1—C2	1.3900	C10—H10B	0.9700
			
C17—O2—H2O	108 (3)	C4—C3—H3A	120.0
O5—N3—O4	121.9 (4)	C5—C4—C3	120.0
O5—N3—C13	119.2 (5)	C5—C4—H4A	120.0
O4—N3—C13	118.7 (5)	C3—C4—H4A	120.0
O5A—N3A—O4A	121.8 (6)	C6—C5—C4	120.0
O5A—N3A—C13	118.4 (8)	C6—C5—H5A	120.0
O4A—N3A—C13	118.4 (8)	C4—C5—H5A	120.0
O7—N4—O6	123.1 (3)	C5—C6—C1	120.0
O7—N4—C15	118.5 (3)	C5—C6—H6A	120.0
O6—N4—C15	118.4 (3)	C1—C6—H6A	120.0
C16—C11—C12	121.2 (3)	C2A—C1A—C6A	120.0
C16—C11—C17	118.1 (3)	C2A—C1A—N1	116.8 (8)
C12—C11—C17	120.7 (3)	C6A—C1A—N1	123.1 (8)
O3—C12—C11	120.3 (3)	C1A—C2A—C3A	120.0
O3—C12—C13	124.6 (3)	C1A—C2A—H2AA	120.0
C11—C12—C13	115.1 (3)	C3A—C2A—H2AA	120.0
C14—C13—N3A	115.6 (4)	C4A—C3A—C2A	120.0
C14—C13—C12	122.9 (3)	C4A—C3A—H3AA	120.0
N3A—C13—C12	121.5 (4)	C2A—C3A—H3AA	120.0
C14—C13—N3	117.0 (3)	C3A—C4A—C5A	120.0
C12—C13—N3	120.1 (3)	C3A—C4A—H4AA	120.0
C15—C14—C13	119.1 (3)	C5A—C4A—H4AA	120.0
C15—C14—H14	120.5	C4A—C5A—C6A	120.0
C13—C14—H14	120.5	C4A—C5A—H5AA	120.0
C14—C15—C16	121.2 (3)	C6A—C5A—H5AA	120.0
C14—C15—N4	119.1 (3)	C5A—C6A—C1A	120.0
C16—C15—N4	119.7 (3)	C5A—C6A—H6AA	120.0
C11—C16—C15	120.5 (3)	C1A—C6A—H6AA	120.0
C11—C16—H16	119.8	N1—C7—C8	111.2 (3)
C15—C16—H16	119.8	N1—C7—H7A	109.4
O1—C17—O2	119.9 (3)	C8—C7—H7A	109.4
O1—C17—C11	123.8 (3)	N1—C7—H7B	109.4
O2—C17—C11	116.2 (3)	C8—C7—H7B	109.4
C1A—N1—C7	120.5 (6)	H7A—C7—H7B	108.0
C1—N1—C7	114.3 (3)	N2—C8—C7	109.5 (3)
C1A—N1—C10	112.9 (5)	N2—C8—H8A	109.8
C1—N1—C10	116.6 (3)	C7—C8—H8A	109.8
C7—N1—C10	110.8 (3)	N2—C8—H8B	109.8
C9—N2—C8	109.8 (3)	C7—C8—H8B	109.8
C9—N2—H21	109 (3)	H8A—C8—H8B	108.2
C8—N2—H21	110 (3)	N2—C9—C10	110.0 (4)
C9—N2—H22	109 (3)	N2—C9—H9A	109.7
C8—N2—H22	111 (3)	C10—C9—H9A	109.7
H21—N2—H22	107 (4)	N2—C9—H9B	109.7
C2—C1—C6	120.0	C10—C9—H9B	109.7
C2—C1—N1	119.9 (3)	H9A—C9—H9B	108.2
C6—C1—N1	120.1 (3)	N1—C10—C9	111.2 (3)
C3—C2—C1	120.0	N1—C10—H10A	109.4
C3—C2—H2A	120.0	C9—C10—H10A	109.4
C1—C2—H2A	120.0	N1—C10—H10B	109.4
C2—C3—C4	120.0	C9—C10—H10B	109.4
C2—C3—H3A	120.0	H10A—C10—H10B	108.0
			
C16—C11—C12—O3	179.6 (3)	C7—N1—C1—C2	159.1 (3)
C17—C11—C12—O3	−0.4 (5)	C10—N1—C1—C2	27.7 (5)
C16—C11—C12—C13	−1.2 (5)	C7—N1—C1—C6	−20.2 (4)
C17—C11—C12—C13	178.8 (3)	C10—N1—C1—C6	−151.6 (3)
O5A—N3A—C13—C14	0.4 (19)	C6—C1—C2—C3	0.0
O4A—N3A—C13—C14	−166.2 (13)	N1—C1—C2—C3	−179.3 (4)
O5A—N3A—C13—C12	178.7 (12)	C1—C2—C3—C4	0.0
O4A—N3A—C13—C12	12 (2)	C2—C3—C4—C5	0.0
O3—C12—C13—C14	179.0 (4)	C3—C4—C5—C6	0.0
C11—C12—C13—C14	−0.2 (5)	C4—C5—C6—C1	0.0
O3—C12—C13—N3A	0.8 (10)	C2—C1—C6—C5	0.0
C11—C12—C13—N3A	−178.4 (9)	N1—C1—C6—C5	179.3 (4)
O3—C12—C13—N3	0.2 (6)	C7—N1—C1A—C2A	176.7 (5)
C11—C12—C13—N3	−179.0 (5)	C10—N1—C1A—C2A	42.6 (8)
O5—N3—C13—C14	−18.9 (9)	C7—N1—C1A—C6A	−4.1 (9)
O4—N3—C13—C14	155.4 (6)	C10—N1—C1A—C6A	−138.2 (6)
O5—N3—C13—C12	160.0 (6)	C6A—C1A—C2A—C3A	0.0
O4—N3—C13—C12	−25.7 (9)	N1—C1A—C2A—C3A	179.2 (8)
N3A—C13—C14—C15	179.4 (9)	C1A—C2A—C3A—C4A	0.0
C12—C13—C14—C15	1.1 (5)	C2A—C3A—C4A—C5A	0.0
N3—C13—C14—C15	179.9 (5)	C3A—C4A—C5A—C6A	0.0
C13—C14—C15—C16	−0.6 (6)	C4A—C5A—C6A—C1A	0.0
C13—C14—C15—N4	179.3 (3)	C2A—C1A—C6A—C5A	0.0
O7—N4—C15—C14	−174.3 (4)	N1—C1A—C6A—C5A	−179.2 (9)
O6—N4—C15—C14	6.1 (5)	C1A—N1—C7—C8	168.3 (6)
O7—N4—C15—C16	5.6 (5)	C1—N1—C7—C8	168.9 (3)
O6—N4—C15—C16	−174.0 (3)	C10—N1—C7—C8	−56.9 (4)
C12—C11—C16—C15	1.7 (5)	C9—N2—C8—C7	−58.8 (4)
C17—C11—C16—C15	−178.3 (3)	N1—C7—C8—N2	58.3 (5)
C14—C15—C16—C11	−0.8 (6)	C8—N2—C9—C10	58.3 (4)
N4—C15—C16—C11	179.3 (3)	C1A—N1—C10—C9	−165.4 (6)
C16—C11—C17—O1	−0.9 (6)	C1—N1—C10—C9	−170.9 (4)
C12—C11—C17—O1	179.1 (4)	C7—N1—C10—C9	56.1 (5)
C16—C11—C17—O2	−179.4 (3)	N2—C9—C10—N1	−57.0 (5)
C12—C11—C17—O2	0.6 (5)		

### 4-Phenylpiperazin-1-ium 2-hydroxy-4,6-dinitrophenolate (6) Hydrogen-bond geometry (Å, º)

**Table:**

*D*—H···*A*	*D*—H	H···*A*	*D*···*A*	*D*—H···*A*
O2—H2*O*···O3	0.84 (2)	1.69 (2)	2.487 (3)	156 (4)
N2—H21···O3	0.88 (2)	2.03 (2)	2.873 (4)	159 (3)
N2—H21···O4	0.88 (2)	2.37 (3)	2.950 (6)	123 (3)
N2—H21···O4*A*	0.88 (2)	2.40 (4)	2.966 (10)	122 (3)
N2—H22···O1^i^	0.87 (2)	2.10 (2)	2.947 (4)	164 (4)
N2—H22···O2^i^	0.87 (2)	2.62 (3)	3.270 (4)	132 (3)
C8—H8*A*···O7^ii^	0.97	2.36	3.134 (5)	137
C8—H8*B*···O4	0.97	2.44	3.000 (6)	116
C9—H9*A*···O4*A*	0.97	2.60	3.166 (8)	118
C9—H9*B*···O5^iii^	0.97	2.58	3.311 (8)	132
C9—H9*B*···O5*A*^iii^	0.97	2.29	3.040 (13)	133

Symmetry codes: (i) −*x*+1, −*y*+1, −*z*+1; (ii) −*x*, −*y*+1, −*z*+1; (iii) *x*+1, *y*, *z*.

### 4-Phenylpiperazin-1-ium 2-hydroxy-4,6-dinitrophenolate (7) Crystal data

**Table:**

C_10_H_15_N_2_^+^·C_7_H_3_N_2_O_6_^−^	*Z* = 2
*M**_r_* = 374.35	*F*(000) = 392
Triclinic, *P*1	*D*_x_ = 1.379 Mg m^−^^3^
*a* = 5.707 (2) Å	Mo *K*α radiation, λ = 0.71073 Å
*b* = 12.505 (3) Å	Cell parameters from 838 reflections
*c* = 13.116 (3) Å	θ = 3.1–28.1°
α = 97.41 (2)°	µ = 0.11 mm^−^^1^
β = 93.28 (2)°	*T* = 293 K
γ = 102.82 (2)°	Needle, yellow
*V* = 901.5 (4) Å^3^	0.48 × 0.08 × 0.04 mm

### 4-Phenylpiperazin-1-ium 2-hydroxy-4,6-dinitrophenolate (7) Data collection

**Table:**

Oxford Diffraction Xcalibur with Sapphire CCD diffractometer	2647 reflections with *I* > 2σ(*I*)
Radiation source: Enhance (Mo) X-ray Source	*R*_int_ = 0.087
Rotation method data acquisition using ω scans.	θ_max_ = 28.1°, θ_min_ = 3.2°
Absorption correction: multi-scan (CrysalisRED; Oxford Diffraction, 2007)	*h* = −7→7
*T*_min_ = 0.647, *T*_max_ = 1.000	*k* = −16→15
7800 measured reflections	*l* = −16→17
7800 independent reflections	

### 4-Phenylpiperazin-1-ium 2-hydroxy-4,6-dinitrophenolate (7) Refinement

**Table:**

Refinement on *F*^2^	Primary atom site location: dual
Least-squares matrix: full	Secondary atom site location: difference Fourier map
*R*[*F*^2^ > 2σ(*F*^2^)] = 0.147	Hydrogen site location: mixed
*wR*(*F*^2^) = 0.297	H atoms treated by a mixture of independent and constrained refinement
*S* = 1.13	*w* = 1/[σ^2^(*F*_o_^2^) + (0.0451*P*)^2^ + 3.195*P*] where *P* = (*F*_o_^2^ + 2*F*_c_^2^)/3
7800 reflections	(Δ/σ)_max_ < 0.001
251 parameters	Δρ_max_ = 0.28 e Å^−^^3^
2 restraints	Δρ_min_ = −0.30 e Å^−^^3^

### 4-Phenylpiperazin-1-ium 2-hydroxy-4,6-dinitrophenolate (7) Special details

**Table:**

Geometry. All esds (except the esd in the dihedral angle between two l.s. planes) are estimated using the full covariance matrix. The cell esds are taken into account individually in the estimation of esds in distances, angles and torsion angles; correlations between esds in cell parameters are only used when they are defined by crystal symmetry. An approximate (isotropic) treatment of cell esds is used for estimating esds involving l.s. planes.
Refinement. Refined as a 2-component twin.

### 4-Phenylpiperazin-1-ium 2-hydroxy-4,6-dinitrophenolate (7) Fractional atomic coordinates and isotropic or equivalent isotropic displacement parameters (Å^2^)

**Table:**

	*x*	*y*	*z*	*U*_iso_*/*U*_eq_	
C1	1.122 (2)	0.6276 (12)	0.8824 (9)	0.051 (4)	
C2	1.091 (2)	0.7342 (12)	0.9003 (10)	0.062 (4)	
H2	0.954536	0.750440	0.869306	0.074*	
C3	1.256 (3)	0.8180 (14)	0.9629 (11)	0.089 (5)	
H3	1.229141	0.889054	0.974939	0.107*	
C4	1.462 (3)	0.7954 (17)	1.0076 (11)	0.090 (6)	
H4	1.577146	0.850571	1.049126	0.108*	
C5	1.491 (3)	0.6892 (17)	0.9888 (11)	0.084 (5)	
H5	1.626416	0.672664	1.019709	0.101*	
C6	1.329 (2)	0.6064 (13)	0.9265 (10)	0.066 (4)	
H6	1.358012	0.535803	0.913969	0.079*	
C7	0.956 (2)	0.4298 (10)	0.8199 (9)	0.055 (4)	
H7A	0.973803	0.416389	0.890828	0.066*	
H7B	1.095257	0.415218	0.786306	0.066*	
C8	0.729 (2)	0.3532 (10)	0.7652 (9)	0.056 (4)	
H8A	0.738219	0.276750	0.765936	0.067*	
H8B	0.590703	0.365439	0.800390	0.067*	
C9	0.700 (2)	0.4918 (11)	0.6524 (9)	0.063 (4)	
H9A	0.557086	0.508210	0.681256	0.076*	
H9B	0.694830	0.504487	0.580979	0.076*	
C10	0.920 (2)	0.5662 (10)	0.7114 (9)	0.055 (4)	
H10A	1.061481	0.555114	0.677531	0.066*	
H10B	0.911569	0.642726	0.711107	0.066*	
C11	−0.018 (2)	0.1731 (10)	0.4335 (9)	0.041 (3)	
C12	0.037 (2)	0.0713 (10)	0.4039 (8)	0.046 (3)	
H12	0.169558	0.053646	0.436202	0.055*	
C13	−0.106 (2)	−0.0027 (11)	0.3270 (10)	0.046 (3)	
C14	−0.307 (2)	0.0161 (10)	0.2780 (9)	0.054 (4)	
H14	−0.406231	−0.036584	0.227934	0.065*	
C15	−0.353 (2)	0.1182 (12)	0.3079 (10)	0.052 (4)	
C16	−0.219 (2)	0.1950 (10)	0.3858 (9)	0.047 (3)	
H16	−0.264440	0.261054	0.406096	0.056*	
C17	0.144 (3)	0.2554 (12)	0.5164 (10)	0.056 (4)	
N1	0.9454 (16)	0.5450 (8)	0.8180 (7)	0.046 (3)	
N2	0.7030 (19)	0.3750 (10)	0.6575 (9)	0.060 (3)	
H21	0.585 (14)	0.323 (8)	0.625 (8)	0.072*	
H22	0.817 (15)	0.354 (9)	0.624 (8)	0.072*	
N3	−0.040 (2)	−0.1092 (10)	0.2942 (9)	0.063 (3)	
N4	−0.560 (2)	0.1460 (14)	0.2536 (10)	0.075 (4)	
O1	0.0892 (15)	0.3455 (7)	0.5441 (7)	0.069 (3)	
O2	0.3327 (15)	0.2290 (7)	0.5475 (7)	0.071 (3)	
O3	0.1442 (18)	−0.1237 (7)	0.3359 (7)	0.076 (3)	
O4	−0.1697 (18)	−0.1757 (8)	0.2251 (7)	0.099 (4)	
O5	−0.5922 (18)	0.2403 (11)	0.2752 (8)	0.094 (4)	
O6	−0.6948 (17)	0.0739 (10)	0.1901 (8)	0.090 (4)	

### 4-Phenylpiperazin-1-ium 2-hydroxy-4,6-dinitrophenolate (7) Atomic displacement parameters (Å^2^)

**Table:**

	*U*^11^	*U*^22^	*U*^33^	*U*^12^	*U*^13^	*U*^23^
C1	0.045 (9)	0.059 (11)	0.043 (8)	0.005 (8)	−0.004 (7)	−0.001 (7)
C2	0.068 (10)	0.053 (11)	0.064 (10)	0.012 (9)	0.006 (8)	0.005 (8)
C3	0.102 (14)	0.073 (13)	0.072 (11)	−0.006 (12)	0.001 (10)	−0.014 (9)
C4	0.074 (13)	0.108 (17)	0.057 (10)	−0.029 (12)	−0.011 (9)	−0.012 (11)
C5	0.082 (13)	0.111 (16)	0.055 (10)	0.012 (13)	0.004 (9)	0.017 (10)
C6	0.054 (10)	0.080 (13)	0.053 (9)	0.002 (9)	−0.004 (8)	−0.001 (8)
C7	0.058 (9)	0.061 (11)	0.052 (8)	0.023 (8)	0.007 (7)	0.012 (7)
C8	0.064 (10)	0.039 (9)	0.062 (9)	0.008 (7)	0.011 (8)	0.000 (7)
C9	0.069 (10)	0.055 (11)	0.062 (9)	0.011 (8)	−0.010 (8)	0.006 (7)
C10	0.055 (9)	0.047 (9)	0.058 (9)	0.013 (7)	−0.005 (7)	−0.005 (7)
C11	0.035 (7)	0.026 (8)	0.058 (8)	0.002 (6)	0.010 (7)	0.006 (6)
C12	0.048 (8)	0.048 (9)	0.036 (8)	0.000 (7)	−0.004 (7)	0.013 (6)
C13	0.035 (8)	0.055 (10)	0.050 (8)	0.015 (7)	−0.001 (7)	0.010 (7)
C14	0.050 (9)	0.038 (10)	0.061 (9)	−0.012 (8)	0.004 (8)	0.000 (7)
C15	0.039 (9)	0.067 (11)	0.047 (8)	0.001 (8)	−0.006 (7)	0.018 (8)
C16	0.038 (8)	0.042 (9)	0.068 (9)	0.019 (7)	0.016 (7)	0.010 (7)
C17	0.058 (10)	0.050 (11)	0.052 (9)	0.004 (8)	0.016 (8)	−0.008 (8)
N1	0.048 (7)	0.045 (8)	0.045 (7)	0.015 (6)	0.001 (6)	0.004 (5)
N2	0.050 (8)	0.058 (10)	0.061 (9)	0.005 (6)	0.005 (6)	−0.015 (6)
N3	0.078 (10)	0.053 (9)	0.062 (8)	0.026 (8)	0.003 (7)	−0.002 (7)
N4	0.055 (9)	0.111 (14)	0.069 (9)	0.028 (10)	0.012 (8)	0.028 (9)
O1	0.070 (7)	0.048 (7)	0.083 (7)	0.008 (5)	0.019 (5)	−0.009 (5)
O2	0.051 (6)	0.063 (7)	0.087 (7)	0.012 (5)	−0.013 (6)	−0.021 (5)
O3	0.079 (8)	0.056 (7)	0.096 (8)	0.028 (6)	0.000 (6)	0.001 (5)
O4	0.136 (9)	0.061 (8)	0.083 (7)	0.022 (7)	−0.022 (7)	−0.032 (6)
O5	0.077 (8)	0.130 (12)	0.100 (9)	0.060 (8)	0.019 (6)	0.042 (8)
O6	0.056 (7)	0.132 (11)	0.080 (7)	0.017 (7)	−0.009 (6)	0.029 (7)

### 4-Phenylpiperazin-1-ium 2-hydroxy-4,6-dinitrophenolate (7) Geometric parameters (Å, º)

**Table:**

C1—C6	1.377 (15)	C10—N1	1.461 (13)
C1—C2	1.377 (16)	C10—H10A	0.9700
C1—N1	1.418 (14)	C10—H10B	0.9700
C2—C3	1.384 (17)	C11—C16	1.374 (14)
C2—H2	0.9300	C11—C12	1.390 (15)
C3—C4	1.385 (19)	C11—C17	1.507 (15)
C3—H3	0.9300	C12—C13	1.367 (14)
C4—C5	1.37 (2)	C12—H12	0.9300
C4—H4	0.9300	C13—C14	1.363 (14)
C5—C6	1.365 (18)	C13—N3	1.481 (15)
C5—H5	0.9300	C14—C15	1.373 (16)
C6—H6	0.9300	C14—H14	0.9300
C7—N1	1.459 (13)	C15—C16	1.372 (14)
C7—C8	1.507 (14)	C15—N4	1.473 (15)
C7—H7A	0.9700	C16—H16	0.9300
C7—H7B	0.9700	C17—O1	1.251 (14)
C8—N2	1.479 (15)	C17—O2	1.256 (14)
C8—H8A	0.9700	N2—H21	0.87 (3)
C8—H8B	0.9700	N2—H22	0.87 (3)
C9—N2	1.475 (16)	N3—O3	1.219 (11)
C9—C10	1.492 (14)	N3—O4	1.230 (12)
C9—H9A	0.9700	N4—O5	1.233 (15)
C9—H9B	0.9700	N4—O6	1.234 (14)
			
C6—C1—C2	118.0 (13)	N1—C10—H10B	109.2
C6—C1—N1	122.7 (14)	C9—C10—H10B	109.2
C2—C1—N1	119.3 (13)	H10A—C10—H10B	107.9
C1—C2—C3	121.9 (15)	C16—C11—C12	118.5 (11)
C1—C2—H2	119.1	C16—C11—C17	122.3 (12)
C3—C2—H2	119.1	C12—C11—C17	119.2 (12)
C2—C3—C4	119.5 (17)	C13—C12—C11	119.5 (11)
C2—C3—H3	120.2	C13—C12—H12	120.2
C4—C3—H3	120.2	C11—C12—H12	120.2
C5—C4—C3	117.8 (16)	C14—C13—C12	123.6 (13)
C5—C4—H4	121.1	C14—C13—N3	117.6 (12)
C3—C4—H4	121.1	C12—C13—N3	118.7 (12)
C6—C5—C4	122.9 (17)	C13—C14—C15	115.2 (12)
C6—C5—H5	118.5	C13—C14—H14	122.4
C4—C5—H5	118.5	C15—C14—H14	122.4
C5—C6—C1	119.8 (15)	C16—C15—C14	123.7 (13)
C5—C6—H6	120.1	C16—C15—N4	118.4 (14)
C1—C6—H6	120.1	C14—C15—N4	117.9 (13)
N1—C7—C8	110.1 (10)	C15—C16—C11	119.2 (12)
N1—C7—H7A	109.6	C15—C16—H16	120.4
C8—C7—H7A	109.6	C11—C16—H16	120.4
N1—C7—H7B	109.6	O1—C17—O2	125.5 (13)
C8—C7—H7B	109.6	O1—C17—C11	118.5 (14)
H7A—C7—H7B	108.2	O2—C17—C11	115.8 (13)
N2—C8—C7	109.2 (10)	C1—N1—C7	117.2 (11)
N2—C8—H8A	109.8	C1—N1—C10	113.8 (10)
C7—C8—H8A	109.8	C7—N1—C10	110.0 (9)
N2—C8—H8B	109.8	C9—N2—C8	111.8 (9)
C7—C8—H8B	109.8	C9—N2—H21	119 (9)
H8A—C8—H8B	108.3	C8—N2—H21	107 (8)
N2—C9—C10	109.9 (10)	C9—N2—H22	112 (8)
N2—C9—H9A	109.7	C8—N2—H22	109 (8)
C10—C9—H9A	109.7	H21—N2—H22	96 (10)
N2—C9—H9B	109.7	O3—N3—O4	124.1 (13)
C10—C9—H9B	109.7	O3—N3—C13	117.2 (12)
H9A—C9—H9B	108.2	O4—N3—C13	118.6 (13)
N1—C10—C9	111.9 (11)	O5—N4—O6	123.1 (15)
N1—C10—H10A	109.2	O5—N4—C15	118.1 (15)
C9—C10—H10A	109.2	O6—N4—C15	118.8 (15)
			
C6—C1—C2—C3	−2 (2)	C16—C11—C17—O1	1.7 (18)
N1—C1—C2—C3	179.6 (12)	C12—C11—C17—O1	−178.2 (12)
C1—C2—C3—C4	1 (2)	C16—C11—C17—O2	−174.2 (12)
C2—C3—C4—C5	−1 (2)	C12—C11—C17—O2	5.8 (17)
C3—C4—C5—C6	1 (3)	C6—C1—N1—C7	15.3 (16)
C4—C5—C6—C1	−2 (2)	C2—C1—N1—C7	−166.4 (11)
C2—C1—C6—C5	2.3 (19)	C6—C1—N1—C10	−115.1 (13)
N1—C1—C6—C5	−179.3 (12)	C2—C1—N1—C10	63.3 (15)
N1—C7—C8—N2	58.8 (12)	C8—C7—N1—C1	168.3 (10)
N2—C9—C10—N1	−55.4 (13)	C8—C7—N1—C10	−59.6 (12)
C16—C11—C12—C13	1.7 (17)	C9—C10—N1—C1	−167.8 (10)
C17—C11—C12—C13	−178.4 (11)	C9—C10—N1—C7	58.3 (13)
C11—C12—C13—C14	−1.8 (18)	C10—C9—N2—C8	55.0 (13)
C11—C12—C13—N3	178.0 (11)	C7—C8—N2—C9	−56.9 (13)
C12—C13—C14—C15	2.8 (18)	C14—C13—N3—O3	177.5 (12)
N3—C13—C14—C15	−177.0 (12)	C12—C13—N3—O3	−2.3 (17)
C13—C14—C15—C16	−4.0 (19)	C14—C13—N3—O4	−0.4 (18)
C13—C14—C15—N4	176.5 (11)	C12—C13—N3—O4	179.8 (12)
C14—C15—C16—C11	4.2 (19)	C16—C15—N4—O5	5.4 (18)
N4—C15—C16—C11	−176.4 (11)	C14—C15—N4—O5	−175.0 (14)
C12—C11—C16—C15	−2.8 (16)	C16—C15—N4—O6	−173.4 (13)
C17—C11—C16—C15	177.3 (11)	C14—C15—N4—O6	6.1 (17)

### 4-Phenylpiperazin-1-ium 2-hydroxy-4,6-dinitrophenolate (7) Hydrogen-bond geometry (Å, º)

**Table:**

*D*—H···*A*	*D*—H	H···*A*	*D*···*A*	*D*—H···*A*
C8—H8*A*···O3^i^	0.97	2.43	3.250 (14)	142
C10—H10*B*···O5^ii^	0.97	2.58	3.366 (16)	138
N2—H21···O2	0.87 (3)	1.81 (4)	2.672 (13)	172 (13)
N2—H22···O1^iii^	0.87 (3)	1.94 (4)	2.792 (13)	166 (12)

Symmetry codes: (i) −*x*+1, −*y*, −*z*+1; (ii) −*x*, −*y*+1, −*z*+1; (iii) *x*+1, *y*, *z*.

### 4-Phenylpiperazin-1-ium 2,4,6-trinitrophenolate (8) Crystal data

**Table:**

C_10_H_15_N_2_^+^·C_6_H_2_N_3_O_7_^−^	*F*(000) = 816
*M**_r_* = 391.34	*D*_x_ = 1.484 Mg m^−^^3^
Monoclinic, *P*2_1_/*c*	Mo *K*α radiation, λ = 0.71073 Å
*a* = 8.517 (1) Å	Cell parameters from 2640 reflections
*b* = 6.825 (1) Å	θ = 2.6–27.9°
*c* = 30.265 (4) Å	µ = 0.12 mm^−^^1^
β = 95.33 (1)°	*T* = 293 K
*V* = 1751.7 (4) Å^3^	Prism, yellow
*Z* = 4	0.50 × 0.36 × 0.20 mm

### 4-Phenylpiperazin-1-ium 2,4,6-trinitrophenolate (8) Data collection

**Table:**

Oxford Diffraction Xcalibur with Sapphire CCD diffractometer	2389 reflections with *I* > 2σ(*I*)
Radiation source: Enhance (Mo) X-ray Source	*R*_int_ = 0.076
ω and φ scans	θ_max_ = 28.0°, θ_min_ = 2.6°
Absorption correction: multi-scan (CrysalisRED; Oxford Diffraction, 2007)	*h* = −10→10
*T*_min_ = 0.835, *T*_max_ = 1.000	*k* = −8→4
12427 measured reflections	*l* = −34→38
3893 independent reflections	

### 4-Phenylpiperazin-1-ium 2,4,6-trinitrophenolate (8) Refinement

**Table:**

Refinement on *F*^2^	Secondary atom site location: difference Fourier map
Least-squares matrix: full	Hydrogen site location: mixed
*R*[*F*^2^ > 2σ(*F*^2^)] = 0.064	H atoms treated by a mixture of independent and constrained refinement
*wR*(*F*^2^) = 0.149	*w* = 1/[σ^2^(*F*_o_^2^) + (0.0504*P*)^2^ + 0.8725*P*] where *P* = (*F*_o_^2^ + 2*F*_c_^2^)/3
*S* = 1.05	(Δ/σ)_max_ < 0.001
3893 reflections	Δρ_max_ = 0.26 e Å^−^^3^
260 parameters	Δρ_min_ = −0.20 e Å^−^^3^
0 restraints	Extinction correction: SHELXL-2018/3 (Sheldrick 2018), Fc^*^=kFc[1+0.001xFc^2^λ^3^/sin(2θ)]^-1/4^
Primary atom site location: dual	Extinction coefficient: 0.0131 (17)

### 4-Phenylpiperazin-1-ium 2,4,6-trinitrophenolate (8) Special details

**Table:**

Geometry. All esds (except the esd in the dihedral angle between two l.s. planes) are estimated using the full covariance matrix. The cell esds are taken into account individually in the estimation of esds in distances, angles and torsion angles; correlations between esds in cell parameters are only used when they are defined by crystal symmetry. An approximate (isotropic) treatment of cell esds is used for estimating esds involving l.s. planes.

### 4-Phenylpiperazin-1-ium 2,4,6-trinitrophenolate (8) Fractional atomic coordinates and isotropic or equivalent isotropic displacement parameters (Å^2^)

**Table:**

	*x*	*y*	*z*	*U*_iso_*/*U*_eq_	
C1	0.7177 (3)	0.4179 (4)	0.26101 (7)	0.0433 (6)	
C2	0.7900 (3)	0.5991 (4)	0.25921 (9)	0.0570 (7)	
H2	0.780507	0.690261	0.281649	0.068*	
C3	0.8768 (3)	0.6449 (5)	0.22386 (10)	0.0688 (9)	
H3	0.923005	0.768012	0.222678	0.083*	
C4	0.8954 (3)	0.5133 (6)	0.19100 (10)	0.0694 (9)	
H4	0.953581	0.545868	0.167535	0.083*	
C5	0.8277 (3)	0.3334 (5)	0.19292 (9)	0.0669 (8)	
H5	0.841487	0.241929	0.170835	0.080*	
C6	0.7388 (3)	0.2847 (4)	0.22725 (8)	0.0556 (7)	
H6	0.692475	0.161451	0.227796	0.067*	
C7	0.6520 (3)	0.1721 (4)	0.31432 (9)	0.0593 (7)	
H7A	0.753277	0.171429	0.331875	0.071*	
H7B	0.656412	0.077286	0.290590	0.071*	
C8	0.5257 (3)	0.1140 (4)	0.34299 (9)	0.0608 (8)	
H8A	0.425199	0.106259	0.325154	0.073*	
H8B	0.549272	−0.014205	0.355756	0.073*	
C9	0.4869 (3)	0.4552 (5)	0.35981 (10)	0.0659 (8)	
H9A	0.485333	0.550341	0.383577	0.079*	
H9B	0.384923	0.457927	0.342652	0.079*	
C10	0.6136 (3)	0.5090 (4)	0.33044 (9)	0.0574 (7)	
H10A	0.591245	0.637189	0.317528	0.069*	
H10B	0.714497	0.515872	0.348122	0.069*	
C11	−0.1176 (3)	0.2795 (3)	0.46054 (8)	0.0401 (5)	
C12	0.0517 (3)	0.2586 (3)	0.46416 (7)	0.0378 (5)	
C13	0.1453 (3)	0.2289 (3)	0.50261 (8)	0.0403 (5)	
H13	0.253635	0.212835	0.502247	0.048*	
C14	0.0762 (3)	0.2232 (3)	0.54215 (7)	0.0393 (5)	
C15	−0.0855 (3)	0.2447 (3)	0.54264 (8)	0.0406 (6)	
H15	−0.131175	0.242523	0.569377	0.049*	
C16	−0.1766 (3)	0.2689 (3)	0.50371 (8)	0.0395 (5)	
N1	0.6227 (2)	0.3663 (3)	0.29532 (6)	0.0426 (5)	
N2	0.5157 (3)	0.2589 (4)	0.37874 (8)	0.0600 (7)	
H21	0.446 (4)	0.227 (4)	0.3950 (10)	0.072*	
H22	0.614 (4)	0.265 (4)	0.3984 (10)	0.072*	
N3	0.1308 (3)	0.2645 (3)	0.42356 (7)	0.0491 (5)	
N4	0.1728 (3)	0.1928 (3)	0.58323 (7)	0.0551 (6)	
N5	−0.3455 (3)	0.2877 (4)	0.50670 (8)	0.0561 (6)	
O1	−0.1980 (2)	0.3022 (3)	0.42443 (6)	0.0645 (6)	
O2	0.2413 (2)	0.1487 (3)	0.42128 (6)	0.0648 (6)	
O3	0.0884 (3)	0.3800 (3)	0.39444 (6)	0.0705 (6)	
O4	0.3160 (3)	0.1892 (4)	0.58218 (7)	0.0770 (7)	
O5	0.1098 (3)	0.1737 (3)	0.61764 (6)	0.0763 (7)	
O6	−0.3873 (3)	0.3692 (4)	0.53943 (8)	0.0982 (9)	
O7	−0.4365 (2)	0.2218 (4)	0.47735 (8)	0.0836 (7)	

### 4-Phenylpiperazin-1-ium 2,4,6-trinitrophenolate (8) Atomic displacement parameters (Å^2^)

**Table:**

	*U*^11^	*U*^22^	*U*^33^	*U*^12^	*U*^13^	*U*^23^
C1	0.0335 (12)	0.0615 (16)	0.0346 (12)	0.0028 (11)	0.0017 (10)	0.0009 (11)
C2	0.0524 (16)	0.0688 (19)	0.0505 (16)	−0.0122 (14)	0.0088 (13)	−0.0012 (14)
C3	0.0500 (17)	0.092 (2)	0.0650 (19)	−0.0185 (15)	0.0072 (14)	0.0147 (18)
C4	0.0457 (16)	0.117 (3)	0.0474 (17)	0.0019 (17)	0.0123 (13)	0.0096 (18)
C5	0.0599 (18)	0.098 (3)	0.0439 (16)	0.0138 (17)	0.0087 (13)	−0.0068 (16)
C6	0.0541 (16)	0.0721 (19)	0.0410 (14)	0.0017 (13)	0.0066 (12)	−0.0043 (14)
C7	0.0650 (18)	0.0619 (18)	0.0530 (16)	0.0100 (14)	0.0154 (13)	0.0096 (14)
C8	0.0599 (17)	0.0671 (19)	0.0548 (17)	−0.0037 (14)	0.0028 (13)	0.0169 (15)
C9	0.0609 (18)	0.079 (2)	0.0621 (18)	−0.0024 (15)	0.0273 (15)	−0.0079 (16)
C10	0.0589 (17)	0.0638 (18)	0.0524 (16)	−0.0096 (13)	0.0198 (13)	−0.0097 (14)
C11	0.0419 (13)	0.0392 (13)	0.0393 (13)	−0.0045 (10)	0.0040 (10)	−0.0039 (10)
C12	0.0437 (13)	0.0337 (12)	0.0375 (12)	−0.0070 (10)	0.0114 (10)	−0.0035 (10)
C13	0.0387 (12)	0.0326 (12)	0.0501 (14)	−0.0034 (10)	0.0059 (10)	−0.0017 (11)
C14	0.0481 (14)	0.0324 (12)	0.0369 (13)	−0.0025 (10)	0.0012 (10)	0.0030 (10)
C15	0.0519 (14)	0.0337 (12)	0.0377 (13)	−0.0024 (10)	0.0119 (11)	−0.0022 (10)
C16	0.0401 (12)	0.0337 (12)	0.0458 (14)	−0.0007 (10)	0.0094 (10)	−0.0039 (10)
N1	0.0411 (11)	0.0501 (12)	0.0374 (10)	−0.0014 (9)	0.0082 (8)	−0.0003 (9)
N2	0.0369 (12)	0.102 (2)	0.0419 (13)	−0.0107 (12)	0.0070 (10)	0.0129 (13)
N3	0.0497 (13)	0.0538 (14)	0.0456 (13)	−0.0141 (11)	0.0136 (10)	−0.0062 (11)
N4	0.0633 (15)	0.0533 (14)	0.0476 (13)	−0.0044 (11)	−0.0016 (11)	0.0095 (11)
N5	0.0466 (13)	0.0710 (16)	0.0522 (14)	0.0037 (11)	0.0115 (11)	−0.0040 (12)
O1	0.0492 (11)	0.0992 (16)	0.0439 (11)	−0.0128 (10)	−0.0022 (8)	0.0049 (10)
O2	0.0475 (11)	0.0813 (15)	0.0695 (13)	−0.0011 (10)	0.0256 (9)	−0.0093 (11)
O3	0.0900 (16)	0.0759 (15)	0.0487 (11)	−0.0030 (11)	0.0225 (11)	0.0104 (11)
O4	0.0533 (13)	0.1053 (18)	0.0699 (14)	0.0007 (11)	−0.0073 (10)	0.0184 (12)
O5	0.0829 (15)	0.1026 (18)	0.0430 (11)	−0.0078 (12)	0.0044 (10)	0.0215 (11)
O6	0.0651 (15)	0.161 (3)	0.0722 (15)	0.0199 (15)	0.0266 (12)	−0.0310 (16)
O7	0.0460 (12)	0.131 (2)	0.0738 (15)	−0.0125 (12)	0.0081 (11)	−0.0182 (14)

### 4-Phenylpiperazin-1-ium 2,4,6-trinitrophenolate (8) Geometric parameters (Å, º)

**Table:**

C1—C2	1.385 (4)	C10—N1	1.449 (3)
C1—C6	1.392 (3)	C10—H10A	0.9700
C1—N1	1.419 (3)	C10—H10B	0.9700
C2—C3	1.391 (4)	C11—O1	1.244 (3)
C2—H2	0.9300	C11—C12	1.443 (3)
C3—C4	1.361 (4)	C11—C16	1.445 (3)
C3—H3	0.9300	C12—C13	1.363 (3)
C4—C5	1.359 (4)	C12—N3	1.456 (3)
C4—H4	0.9300	C13—C14	1.382 (3)
C5—C6	1.382 (4)	C13—H13	0.9300
C5—H5	0.9300	C14—C15	1.386 (3)
C6—H6	0.9300	C14—N4	1.441 (3)
C7—N1	1.457 (3)	C15—C16	1.360 (3)
C7—C8	1.497 (4)	C15—H15	0.9300
C7—H7A	0.9700	C16—N5	1.455 (3)
C7—H7B	0.9700	N2—H21	0.83 (3)
C8—N2	1.474 (4)	N2—H22	0.98 (3)
C8—H8A	0.9700	N3—O3	1.212 (3)
C8—H8B	0.9700	N3—O2	1.236 (3)
C9—N2	1.469 (4)	N4—O5	1.222 (3)
C9—C10	1.506 (3)	N4—O4	1.223 (3)
C9—H9A	0.9700	N5—O7	1.210 (3)
C9—H9B	0.9700	N5—O6	1.218 (3)
			
C2—C1—C6	117.8 (2)	N1—C10—H10B	109.4
C2—C1—N1	122.4 (2)	C9—C10—H10B	109.4
C6—C1—N1	119.8 (2)	H10A—C10—H10B	108.0
C1—C2—C3	120.1 (3)	O1—C11—C12	122.9 (2)
C1—C2—H2	120.0	O1—C11—C16	126.2 (2)
C3—C2—H2	120.0	C12—C11—C16	110.8 (2)
C4—C3—C2	121.3 (3)	C13—C12—C11	125.5 (2)
C4—C3—H3	119.3	C13—C12—N3	116.4 (2)
C2—C3—H3	119.3	C11—C12—N3	118.1 (2)
C5—C4—C3	119.1 (3)	C12—C13—C14	118.7 (2)
C5—C4—H4	120.4	C12—C13—H13	120.6
C3—C4—H4	120.4	C14—C13—H13	120.6
C4—C5—C6	120.9 (3)	C13—C14—C15	120.6 (2)
C4—C5—H5	119.6	C13—C14—N4	119.7 (2)
C6—C5—H5	119.6	C15—C14—N4	119.6 (2)
C5—C6—C1	120.8 (3)	C16—C15—C14	119.4 (2)
C5—C6—H6	119.6	C16—C15—H15	120.3
C1—C6—H6	119.6	C14—C15—H15	120.3
N1—C7—C8	111.3 (2)	C15—C16—C11	124.8 (2)
N1—C7—H7A	109.4	C15—C16—N5	116.4 (2)
C8—C7—H7A	109.4	C11—C16—N5	118.7 (2)
N1—C7—H7B	109.4	C1—N1—C10	116.3 (2)
C8—C7—H7B	109.4	C1—N1—C7	115.2 (2)
H7A—C7—H7B	108.0	C10—N1—C7	109.9 (2)
N2—C8—C7	110.0 (2)	C9—N2—C8	110.2 (2)
N2—C8—H8A	109.7	C9—N2—H21	112 (2)
C7—C8—H8A	109.7	C8—N2—H21	111 (2)
N2—C8—H8B	109.7	C9—N2—H22	107.5 (17)
C7—C8—H8B	109.7	C8—N2—H22	111.7 (17)
H8A—C8—H8B	108.2	H21—N2—H22	105 (3)
N2—C9—C10	110.5 (2)	O3—N3—O2	123.5 (2)
N2—C9—H9A	109.6	O3—N3—C12	120.1 (2)
C10—C9—H9A	109.6	O2—N3—C12	116.3 (2)
N2—C9—H9B	109.6	O5—N4—O4	122.5 (2)
C10—C9—H9B	109.6	O5—N4—C14	119.4 (2)
H9A—C9—H9B	108.1	O4—N4—C14	118.1 (2)
N1—C10—C9	111.0 (2)	O7—N5—O6	123.4 (2)
N1—C10—H10A	109.4	O7—N5—C16	119.4 (2)
C9—C10—H10A	109.4	O6—N5—C16	117.1 (2)
			
C6—C1—C2—C3	1.7 (4)	O1—C11—C16—N5	−0.6 (4)
N1—C1—C2—C3	−177.4 (2)	C12—C11—C16—N5	179.8 (2)
C1—C2—C3—C4	−1.3 (4)	C2—C1—N1—C10	−4.1 (3)
C2—C3—C4—C5	−0.1 (5)	C6—C1—N1—C10	176.8 (2)
C3—C4—C5—C6	1.1 (5)	C2—C1—N1—C7	−134.9 (3)
C4—C5—C6—C1	−0.7 (4)	C6—C1—N1—C7	46.0 (3)
C2—C1—C6—C5	−0.7 (4)	C9—C10—N1—C1	169.3 (2)
N1—C1—C6—C5	178.5 (2)	C9—C10—N1—C7	−57.6 (3)
N1—C7—C8—N2	−58.0 (3)	C8—C7—N1—C1	−168.0 (2)
N2—C9—C10—N1	57.4 (3)	C8—C7—N1—C10	58.3 (3)
O1—C11—C12—C13	−178.4 (2)	C10—C9—N2—C8	−56.6 (3)
C16—C11—C12—C13	1.2 (3)	C7—C8—N2—C9	56.8 (3)
O1—C11—C12—N3	0.0 (3)	C13—C12—N3—O3	−141.4 (2)
C16—C11—C12—N3	179.6 (2)	C11—C12—N3—O3	40.1 (3)
C11—C12—C13—C14	−2.0 (3)	C13—C12—N3—O2	37.9 (3)
N3—C12—C13—C14	179.6 (2)	C11—C12—N3—O2	−140.6 (2)
C12—C13—C14—C15	0.8 (3)	C13—C14—N4—O5	−174.7 (2)
C12—C13—C14—N4	−179.8 (2)	C15—C14—N4—O5	4.7 (3)
C13—C14—C15—C16	0.9 (3)	C13—C14—N4—O4	6.0 (3)
N4—C14—C15—C16	−178.5 (2)	C15—C14—N4—O4	−174.6 (2)
C14—C15—C16—C11	−1.7 (4)	C15—C16—N5—O7	−146.1 (3)
C14—C15—C16—N5	179.1 (2)	C11—C16—N5—O7	34.6 (3)
O1—C11—C16—C15	−179.7 (2)	C15—C16—N5—O6	33.4 (3)
C12—C11—C16—C15	0.6 (3)	C11—C16—N5—O6	−145.9 (3)

### 4-Phenylpiperazin-1-ium 2,4,6-trinitrophenolate (8) Hydrogen-bond geometry (Å, º)

**Table:**

*D*—H···*A*	*D*—H	H···*A*	*D*···*A*	*D*—H···*A*
C8—H8*B*···O4^i^	0.97	2.42	3.265 (4)	145
C9—H9*A*···O4^ii^	0.97	2.60	3.353 (4)	134
C9—H9*A*···O6^iii^	0.97	2.61	3.455 (4)	146
N2—H21···O2	0.83 (3)	2.06 (3)	2.871 (3)	166 (3)
N2—H21···O7^iv^	0.83 (3)	2.60 (3)	2.985 (3)	110 (2)
N2—H22···O1^iv^	0.98 (3)	1.74 (3)	2.705 (3)	168 (3)
N2—H21···O7^iv^	0.83 (3)	2.60 (3)	2.985 (3)	110 (2)

Symmetry codes: (i) −*x*+1, −*y*, −*z*+1; (ii) −*x*+1, −*y*+1, −*z*+1; (iii) −*x*, −*y*+1, −*z*+1; (iv) *x*+1, *y*, *z*.

### 4-Phenylpiperazin-1-ium benzoate monohydrate (9) Crystal data

**Table:**

C_10_H_15_N_2_^+^·C_7_H_5_O_2_^−^·H_2_O	*F*(000) = 648
*M**_r_* = 302.36	*D*_x_ = 1.236 Mg m^−^^3^
Monoclinic, *P*2_1_/*c*	Mo *K*α radiation, λ = 0.71073 Å
*a* = 6.202 (2) Å	Cell parameters from 885 reflections
*b* = 34.573 (9) Å	θ = 2.7–27.8°
*c* = 7.596 (2) Å	µ = 0.09 mm^−^^1^
β = 93.83 (2)°	*T* = 293 K
*V* = 1625.1 (8) Å^3^	Rod, colourless
*Z* = 4	0.32 × 0.20 × 0.16 mm

### 4-Phenylpiperazin-1-ium benzoate monohydrate (9) Data collection

**Table:**

Oxford Diffraction Xcalibur with Sapphire CCD diffractometer	1387 reflections with *I* > 2σ(*I*)
Radiation source: Enhance (Mo) X-ray Source	*R*_int_ = 0.039
ω and φ scans	θ_max_ = 27.8°, θ_min_ = 2.9°
Absorption correction: multi-scan (CrysalisRED; Oxford Diffraction, 2007)	*h* = −7→8
*T*_min_ = 0.985, *T*_max_ = 1.000	*k* = −32→44
6075 measured reflections	*l* = −8→9
3492 independent reflections	

### 4-Phenylpiperazin-1-ium benzoate monohydrate (9) Refinement

**Table:**

Refinement on *F*^2^	Primary atom site location: dual
Least-squares matrix: full	Secondary atom site location: difference Fourier map
*R*[*F*^2^ > 2σ(*F*^2^)] = 0.065	Hydrogen site location: mixed
*wR*(*F*^2^) = 0.144	H atoms treated by a mixture of independent and constrained refinement
*S* = 0.95	*w* = 1/[σ^2^(*F*_o_^2^) + (0.0549*P*)^2^] where *P* = (*F*_o_^2^ + 2*F*_c_^2^)/3
3492 reflections	(Δ/σ)_max_ < 0.001
211 parameters	Δρ_max_ = 0.24 e Å^−^^3^
4 restraints	Δρ_min_ = −0.16 e Å^−^^3^

### 4-Phenylpiperazin-1-ium benzoate monohydrate (9) Special details

**Table:**

Geometry. All esds (except the esd in the dihedral angle between two l.s. planes) are estimated using the full covariance matrix. The cell esds are taken into account individually in the estimation of esds in distances, angles and torsion angles; correlations between esds in cell parameters are only used when they are defined by crystal symmetry. An approximate (isotropic) treatment of cell esds is used for estimating esds involving l.s. planes.

### 4-Phenylpiperazin-1-ium benzoate monohydrate (9) Fractional atomic coordinates and isotropic or equivalent isotropic displacement parameters (Å^2^)

**Table:**

	*x*	*y*	*z*	*U*_iso_*/*U*_eq_	
N1	0.2813 (3)	0.60989 (6)	0.7704 (3)	0.0434 (6)	
N2	0.2143 (4)	0.52769 (7)	0.7340 (3)	0.0540 (7)	
H21	0.175 (4)	0.5046 (6)	0.776 (3)	0.065*	
H22	0.244 (4)	0.5249 (8)	0.621 (2)	0.065*	
C1	0.3322 (4)	0.64902 (8)	0.7364 (3)	0.0458 (7)	
C2	0.1985 (5)	0.67263 (9)	0.6301 (4)	0.0732 (9)	
H2	0.073519	0.662304	0.574143	0.088*	
C3	0.2469 (6)	0.71136 (9)	0.6050 (5)	0.0890 (11)	
H3	0.153321	0.726509	0.533416	0.107*	
C4	0.4287 (7)	0.72754 (10)	0.6834 (5)	0.0871 (11)	
H4	0.460834	0.753486	0.666273	0.105*	
C5	0.5623 (6)	0.70463 (10)	0.7878 (5)	0.0849 (11)	
H5	0.687062	0.715266	0.842693	0.102*	
C6	0.5172 (5)	0.66585 (9)	0.8144 (4)	0.0684 (9)	
H6	0.612551	0.650963	0.885571	0.082*	
C7	0.4623 (4)	0.58257 (7)	0.7779 (4)	0.0500 (7)	
H7A	0.579852	0.592960	0.854289	0.060*	
H7B	0.513636	0.579710	0.660752	0.060*	
C8	0.4003 (4)	0.54349 (7)	0.8453 (4)	0.0554 (8)	
H8A	0.522296	0.525995	0.842923	0.067*	
H8B	0.361408	0.545749	0.966498	0.067*	
C9	0.0303 (4)	0.55502 (8)	0.7319 (4)	0.0586 (8)	
H9A	−0.018777	0.557418	0.849982	0.070*	
H9B	−0.088519	0.545041	0.655591	0.070*	
C10	0.0951 (4)	0.59416 (8)	0.6670 (4)	0.0544 (7)	
H10A	0.129289	0.592100	0.544591	0.065*	
H10B	−0.025699	0.611834	0.672570	0.065*	
O1	0.0826 (4)	0.45352 (6)	0.8352 (3)	0.0739 (6)	
O2	0.3636 (4)	0.44271 (8)	0.6833 (4)	0.1271 (11)	
C11	0.1746 (4)	0.38836 (9)	0.7732 (3)	0.0488 (7)	
C12	0.3158 (5)	0.36281 (11)	0.6979 (4)	0.0710 (9)	
H12	0.434100	0.372529	0.643180	0.085*	
C13	0.2834 (6)	0.32351 (12)	0.7030 (4)	0.0850 (11)	
H13	0.378578	0.306980	0.650576	0.102*	
C14	0.1131 (7)	0.30867 (10)	0.7841 (5)	0.0841 (10)	
H14	0.092772	0.282041	0.788617	0.101*	
C15	−0.0285 (5)	0.33305 (10)	0.8592 (4)	0.0758 (10)	
H15	−0.145442	0.322946	0.914408	0.091*	
C16	0.0017 (4)	0.37282 (8)	0.8532 (4)	0.0566 (8)	
H16	−0.096028	0.389140	0.903761	0.068*	
C17	0.2108 (5)	0.43133 (10)	0.7647 (4)	0.0605 (8)	
O3	0.7231 (3)	0.47794 (7)	0.6283 (3)	0.0737 (7)	
H31	0.816 (5)	0.4679 (11)	0.697 (4)	0.111*	
H32	0.609 (4)	0.4665 (9)	0.645 (5)	0.111*	

### 4-Phenylpiperazin-1-ium benzoate monohydrate (9) Atomic displacement parameters (Å^2^)

**Table:**

	*U*^11^	*U*^22^	*U*^33^	*U*^12^	*U*^13^	*U*^23^
N1	0.0406 (13)	0.0418 (14)	0.0472 (14)	−0.0004 (11)	−0.0012 (10)	−0.0001 (11)
N2	0.0689 (17)	0.0449 (15)	0.0493 (16)	−0.0092 (14)	0.0112 (14)	0.0018 (14)
C1	0.0524 (17)	0.0389 (16)	0.0463 (17)	−0.0012 (14)	0.0049 (13)	−0.0033 (14)
C2	0.074 (2)	0.050 (2)	0.092 (2)	−0.0010 (17)	−0.0224 (19)	0.0050 (19)
C3	0.109 (3)	0.048 (2)	0.106 (3)	0.004 (2)	−0.026 (2)	0.010 (2)
C4	0.133 (3)	0.044 (2)	0.082 (3)	−0.014 (2)	−0.012 (2)	0.000 (2)
C5	0.105 (3)	0.060 (2)	0.086 (3)	−0.031 (2)	−0.024 (2)	0.000 (2)
C6	0.074 (2)	0.054 (2)	0.075 (2)	−0.0112 (18)	−0.0153 (17)	0.0072 (17)
C7	0.0442 (16)	0.0485 (18)	0.0566 (18)	−0.0019 (14)	−0.0008 (13)	−0.0016 (15)
C8	0.0595 (18)	0.0498 (18)	0.0556 (18)	0.0000 (15)	−0.0066 (15)	0.0025 (15)
C9	0.0512 (17)	0.0590 (19)	0.0659 (19)	−0.0085 (16)	0.0071 (14)	−0.0031 (17)
C10	0.0434 (16)	0.0507 (18)	0.068 (2)	−0.0008 (14)	−0.0024 (14)	0.0025 (16)
O1	0.0960 (16)	0.0529 (14)	0.0741 (15)	−0.0102 (12)	0.0157 (13)	0.0002 (12)
O2	0.0889 (17)	0.104 (2)	0.195 (3)	−0.0264 (15)	0.0568 (19)	0.035 (2)
C11	0.0447 (16)	0.0594 (19)	0.0417 (16)	0.0016 (15)	−0.0014 (13)	0.0053 (15)
C12	0.062 (2)	0.089 (3)	0.063 (2)	0.017 (2)	0.0069 (16)	0.0160 (19)
C13	0.106 (3)	0.077 (3)	0.072 (2)	0.040 (2)	0.007 (2)	0.002 (2)
C14	0.125 (3)	0.053 (2)	0.073 (2)	0.003 (2)	−0.009 (2)	−0.004 (2)
C15	0.087 (2)	0.062 (2)	0.079 (2)	−0.023 (2)	0.0130 (19)	0.000 (2)
C16	0.0602 (18)	0.055 (2)	0.0557 (18)	−0.0061 (16)	0.0122 (15)	−0.0043 (15)
C17	0.056 (2)	0.066 (2)	0.059 (2)	−0.0128 (18)	−0.0014 (16)	0.0198 (18)
O3	0.0697 (16)	0.0830 (17)	0.0700 (15)	−0.0213 (13)	0.0159 (12)	−0.0070 (13)

### 4-Phenylpiperazin-1-ium benzoate monohydrate (9) Geometric parameters (Å, º)

**Table:**

N1—C1	1.417 (3)	C8—H8B	0.9700
N1—C10	1.458 (3)	C9—C10	1.504 (3)
N1—C7	1.465 (3)	C9—H9A	0.9700
N2—C9	1.481 (3)	C9—H9B	0.9700
N2—C8	1.488 (3)	C10—H10A	0.9700
N2—H21	0.898 (17)	C10—H10B	0.9700
N2—H22	0.893 (16)	O1—C17	1.251 (3)
C1—C6	1.384 (3)	O2—C17	1.230 (3)
C1—C2	1.384 (3)	C11—C16	1.376 (3)
C2—C3	1.388 (4)	C11—C12	1.393 (4)
C2—H2	0.9300	C11—C17	1.504 (4)
C3—C4	1.360 (4)	C12—C13	1.375 (4)
C3—H3	0.9300	C12—H12	0.9300
C4—C5	1.362 (4)	C13—C14	1.358 (4)
C4—H4	0.9300	C13—H13	0.9300
C5—C6	1.387 (4)	C14—C15	1.369 (4)
C5—H5	0.9300	C14—H14	0.9300
C6—H6	0.9300	C15—C16	1.389 (4)
C7—C8	1.504 (3)	C15—H15	0.9300
C7—H7A	0.9700	C16—H16	0.9300
C7—H7B	0.9700	O3—H31	0.830 (18)
C8—H8A	0.9700	O3—H32	0.826 (18)
			
C1—N1—C10	115.8 (2)	C7—C8—H8B	109.7
C1—N1—C7	116.32 (19)	H8A—C8—H8B	108.2
C10—N1—C7	110.9 (2)	N2—C9—C10	110.8 (2)
C9—N2—C8	109.8 (2)	N2—C9—H9A	109.5
C9—N2—H21	110.3 (17)	C10—C9—H9A	109.5
C8—N2—H21	110.0 (17)	N2—C9—H9B	109.5
C9—N2—H22	105.5 (17)	C10—C9—H9B	109.5
C8—N2—H22	112.5 (17)	H9A—C9—H9B	108.1
H21—N2—H22	109 (3)	N1—C10—C9	112.3 (2)
C6—C1—C2	116.7 (3)	N1—C10—H10A	109.2
C6—C1—N1	120.8 (2)	C9—C10—H10A	109.2
C2—C1—N1	122.5 (3)	N1—C10—H10B	109.2
C1—C2—C3	121.5 (3)	C9—C10—H10B	109.2
C1—C2—H2	119.3	H10A—C10—H10B	107.9
C3—C2—H2	119.3	C16—C11—C12	117.6 (3)
C4—C3—C2	121.2 (3)	C16—C11—C17	121.8 (3)
C4—C3—H3	119.4	C12—C11—C17	120.6 (3)
C2—C3—H3	119.4	C13—C12—C11	121.2 (3)
C3—C4—C5	118.0 (3)	C13—C12—H12	119.4
C3—C4—H4	121.0	C11—C12—H12	119.4
C5—C4—H4	121.0	C14—C13—C12	120.4 (3)
C4—C5—C6	121.7 (3)	C14—C13—H13	119.8
C4—C5—H5	119.1	C12—C13—H13	119.8
C6—C5—H5	119.1	C13—C14—C15	119.7 (3)
C1—C6—C5	120.9 (3)	C13—C14—H14	120.1
C1—C6—H6	119.5	C15—C14—H14	120.1
C5—C6—H6	119.5	C14—C15—C16	120.3 (3)
N1—C7—C8	112.3 (2)	C14—C15—H15	119.9
N1—C7—H7A	109.1	C16—C15—H15	119.9
C8—C7—H7A	109.1	C11—C16—C15	120.8 (3)
N1—C7—H7B	109.1	C11—C16—H16	119.6
C8—C7—H7B	109.1	C15—C16—H16	119.6
H7A—C7—H7B	107.9	O2—C17—O1	123.5 (3)
N2—C8—C7	110.0 (2)	O2—C17—C11	117.4 (3)
N2—C8—H8A	109.7	O1—C17—C11	119.1 (3)
C7—C8—H8A	109.7	H31—O3—H32	106 (4)
N2—C8—H8B	109.7		
			
C10—N1—C1—C6	−173.8 (2)	C8—N2—C9—C10	57.0 (3)
C7—N1—C1—C6	−40.8 (3)	C1—N1—C10—C9	−170.5 (2)
C10—N1—C1—C2	8.5 (3)	C7—N1—C10—C9	54.1 (3)
C7—N1—C1—C2	141.5 (3)	N2—C9—C10—N1	−56.0 (3)
C6—C1—C2—C3	−0.8 (4)	C16—C11—C12—C13	0.1 (4)
N1—C1—C2—C3	177.0 (3)	C17—C11—C12—C13	−179.3 (3)
C1—C2—C3—C4	0.4 (5)	C11—C12—C13—C14	−0.8 (5)
C2—C3—C4—C5	−0.1 (5)	C12—C13—C14—C15	0.9 (5)
C3—C4—C5—C6	0.2 (5)	C13—C14—C15—C16	−0.2 (5)
C2—C1—C6—C5	0.8 (4)	C12—C11—C16—C15	0.5 (4)
N1—C1—C6—C5	−177.0 (3)	C17—C11—C16—C15	179.9 (3)
C4—C5—C6—C1	−0.6 (5)	C14—C15—C16—C11	−0.5 (5)
C1—N1—C7—C8	170.0 (2)	C16—C11—C17—O2	−176.6 (3)
C10—N1—C7—C8	−54.7 (3)	C12—C11—C17—O2	2.8 (4)
C9—N2—C8—C7	−57.2 (3)	C16—C11—C17—O1	1.4 (4)
N1—C7—C8—N2	56.6 (3)	C12—C11—C17—O1	−179.2 (3)

### 4-Phenylpiperazin-1-ium benzoate monohydrate (9) Hydrogen-bond geometry (Å, º)

**Table:**

*D*—H···*A*	*D*—H	H···*A*	*D*···*A*	*D*—H···*A*
N2—H21···O1	0.90 (2)	1.92 (2)	2.813 (3)	173 (2)
N2—H21···O2	0.90 (2)	2.56 (2)	3.112 (4)	121 (2)
N2—H22···O3^i^	0.89 (2)	1.92 (2)	2.812 (3)	173 (2)
C9—H9*A*···O1^ii^	0.97	2.48	3.420 (4)	164
C9—H9*B*···O3^iii^	0.97	2.60	3.340 (4)	133
O3—H31···O1^iv^	0.83 (2)	1.96 (2)	2.772 (3)	166 (4)
O3—H32···O2	0.83 (2)	1.77 (2)	2.599 (3)	179 (4)

Symmetry codes: (i) −*x*+1, −*y*+1, −*z*+1; (ii) −*x*, −*y*+1, −*z*+2; (iii) *x*−1, *y*, *z*; (iv) *x*+1, *y*, *z*.

### 4-Phenylpiperazin-1-ium 4-methylbenzenesulfonate (10) Crystal data

**Table:**

C_10_H_15_N_2_^+^·C_7_H_7_O_3_S^−^	*F*(000) = 712
*M**_r_* = 334.42	*D*_x_ = 1.325 Mg m^−^^3^
Monoclinic, *P*2_1_	Mo *K*α radiation, λ = 0.71073 Å
*a* = 8.325 (1) Å	Cell parameters from 2252 reflections
*b* = 10.949 (2) Å	θ = 2.9–27.6°
*c* = 18.418 (4) Å	µ = 0.21 mm^−^^1^
β = 92.67 (2)°	*T* = 293 K
*V* = 1677.0 (5) Å^3^	Plate, colourless
*Z* = 4	0.50 × 0.36 × 0.14 mm

### 4-Phenylpiperazin-1-ium 4-methylbenzenesulfonate (10) Data collection

**Table:**

Oxford Diffraction Xcalibur with Sapphire CCD diffractometer	2767 reflections with *I* > 2σ(*I*)
Radiation source: Enhance (Mo) X-ray Source	*R*_int_ = 0.044
Rotation method data acquisition using ω scans.	θ_max_ = 27.7°, θ_min_ = 2.9°
Absorption correction: multi-scan (CrysalisRED; Oxford Diffraction, 2007)	*h* = −10→9
*T*_min_ = 0.696, *T*_max_ = 1.000	*k* = −14→11
6123 measured reflections	*l* = −24→20
4918 independent reflections	

### 4-Phenylpiperazin-1-ium 4-methylbenzenesulfonate (10) Refinement

**Table:**

Refinement on *F*^2^	Secondary atom site location: difference Fourier map
Least-squares matrix: full	Hydrogen site location: inferred from neighbouring sites
*R*[*F*^2^ > 2σ(*F*^2^)] = 0.126	H-atom parameters constrained
*wR*(*F*^2^) = 0.298	*w* = 1/[σ^2^(*F*_o_^2^) + (0.0541*P*)^2^ + 11.2654*P*] where *P* = (*F*_o_^2^ + 2*F*_c_^2^)/3
*S* = 1.12	(Δ/σ)_max_ < 0.001
4918 reflections	Δρ_max_ = 1.08 e Å^−^^3^
480 parameters	Δρ_min_ = −0.41 e Å^−^^3^
853 restraints	Absolute structure: Flack *x* determined using 597 quotients [(*I*^+^)-(*I*^-^)]/[(*I*^+^)+(*I*^-^)] (Parsons *et al.*, 2013)
Primary atom site location: dual	Absolute structure parameter: 0.00 (11)

### 4-Phenylpiperazin-1-ium 4-methylbenzenesulfonate (10) Special details

**Table:**

Geometry. All esds (except the esd in the dihedral angle between two l.s. planes) are estimated using the full covariance matrix. The cell esds are taken into account individually in the estimation of esds in distances, angles and torsion angles; correlations between esds in cell parameters are only used when they are defined by crystal symmetry. An approximate (isotropic) treatment of cell esds is used for estimating esds involving l.s. planes.
Refinement. Refined as a 2-component twin.

### 4-Phenylpiperazin-1-ium 4-methylbenzenesulfonate (10) Fractional atomic coordinates and isotropic or equivalent isotropic displacement parameters (Å^2^)

**Table:**

	*x*	*y*	*z*	*U*_iso_*/*U*_eq_	Occ. (<1)
N1	0.3198 (15)	0.0451 (12)	0.1981 (8)	0.044 (3)	
N2	0.3315 (13)	−0.0070 (11)	0.0453 (8)	0.041 (3)	
H21N	0.301100	0.008772	−0.000733	0.050*	
H22N	0.400866	−0.068854	0.045658	0.050*	
C1	0.345 (5)	0.050 (4)	0.2756 (14)	0.056 (4)	0.49 (7)
C2	0.244 (4)	−0.010 (4)	0.322 (2)	0.058 (4)	0.49 (7)
H2	0.163707	−0.061632	0.302457	0.069*	0.49 (7)
C3	0.263 (4)	0.006 (5)	0.3965 (19)	0.060 (4)	0.49 (7)
H3	0.195547	−0.034965	0.427260	0.073*	0.49 (7)
C4	0.383 (5)	0.082 (4)	0.4252 (14)	0.062 (4)	0.49 (7)
H4	0.395978	0.092749	0.475215	0.074*	0.49 (7)
C5	0.484 (6)	0.143 (3)	0.3791 (18)	0.061 (4)	0.49 (7)
H5	0.564573	0.193797	0.398368	0.074*	0.49 (7)
C6	0.465 (5)	0.127 (4)	0.3044 (17)	0.058 (4)	0.49 (7)
H6	0.532736	0.167132	0.273565	0.070*	0.49 (7)
C1A	0.338 (4)	0.047 (3)	0.2748 (13)	0.055 (3)	0.51 (7)
C2A	0.259 (5)	−0.042 (4)	0.3132 (19)	0.057 (4)	0.51 (7)
H2A	0.204660	−0.104759	0.288426	0.069*	0.51 (7)
C3A	0.261 (4)	−0.038 (4)	0.3887 (19)	0.061 (4)	0.51 (7)
H3A	0.207972	−0.097323	0.414372	0.073*	0.51 (7)
C4A	0.342 (5)	0.056 (4)	0.4258 (13)	0.062 (4)	0.51 (7)
H4A	0.342913	0.059306	0.476246	0.075*	0.51 (7)
C5A	0.420 (7)	0.146 (2)	0.3874 (18)	0.060 (4)	0.51 (7)
H5A	0.474543	0.208501	0.412173	0.072*	0.51 (7)
C6A	0.418 (5)	0.141 (3)	0.3119 (18)	0.059 (4)	0.51 (7)
H6A	0.471233	0.201067	0.286226	0.070*	0.51 (7)
C7	0.4521 (18)	0.0840 (15)	0.1564 (9)	0.049 (4)	
H7A	0.537245	0.023733	0.161521	0.059*	
H7B	0.493647	0.160291	0.176389	0.059*	
C8	0.411 (2)	0.1017 (15)	0.0780 (9)	0.052 (4)	
H8A	0.339429	0.171509	0.071858	0.063*	
H8B	0.507744	0.119164	0.052790	0.063*	
C9	0.1907 (17)	−0.0413 (16)	0.0857 (9)	0.046 (4)	
H9A	0.144478	−0.115915	0.065528	0.056*	
H9B	0.110126	0.022516	0.080775	0.056*	
C10	0.2369 (19)	−0.0603 (15)	0.1653 (10)	0.049 (4)	
H10A	0.140631	−0.076696	0.191354	0.059*	
H10B	0.306204	−0.131345	0.170331	0.059*	
S1	0.1579 (4)	0.7481 (4)	−0.1011 (2)	0.0439 (10)	
O1	0.1787 (12)	0.6159 (9)	−0.0944 (6)	0.049 (3)	
O2	0.2928 (18)	0.8175 (11)	−0.0705 (7)	0.076 (4)	
O3	0.0028 (16)	0.7862 (13)	−0.0762 (7)	0.082 (5)	
C11	0.1506 (16)	0.7750 (13)	−0.1938 (8)	0.036 (3)	
C12	0.2354 (19)	0.8746 (17)	−0.2240 (10)	0.054 (4)	
H12	0.298418	0.925481	−0.193938	0.065*	
C13	0.224 (2)	0.8952 (19)	−0.2971 (11)	0.067 (5)	
H13	0.283222	0.959399	−0.315160	0.081*	
C14	0.133 (2)	0.830 (2)	−0.3450 (11)	0.070 (5)	
C15	0.042 (2)	0.735 (2)	−0.3146 (10)	0.069 (5)	
H15	−0.025524	0.687927	−0.344707	0.083*	
C16	0.051 (2)	0.7130 (15)	−0.2414 (9)	0.060 (5)	
H16	−0.013532	0.652069	−0.223331	0.072*	
C17	0.121 (3)	0.853 (3)	−0.4249 (11)	0.101 (8)	
H17A	0.138438	0.778329	−0.450528	0.152*	
H17B	0.200838	0.911924	−0.437227	0.152*	
H17C	0.015970	0.884299	−0.438319	0.152*	
N3	0.2216 (15)	0.5462 (13)	0.1956 (8)	0.046 (3)	
N4	0.1795 (13)	0.4911 (11)	0.0439 (8)	0.041 (3)	
H41N	0.110853	0.428961	0.045763	0.049*	
H42N	0.200180	0.504089	−0.002420	0.049*	
C18	0.212 (4)	0.553 (3)	0.2730 (13)	0.052 (3)	0.53 (7)
C19	0.321 (4)	0.490 (4)	0.3187 (18)	0.055 (4)	0.53 (7)
H19	0.395669	0.437535	0.299289	0.066*	0.53 (7)
C20	0.319 (3)	0.506 (4)	0.3935 (17)	0.058 (4)	0.53 (7)
H20	0.392116	0.464559	0.424139	0.070*	0.53 (7)
C21	0.208 (5)	0.585 (3)	0.4226 (13)	0.060 (4)	0.53 (7)
H21	0.206786	0.596177	0.472606	0.071*	0.53 (7)
C22	0.099 (6)	0.648 (3)	0.3768 (17)	0.058 (4)	0.53 (7)
H22	0.025007	0.700772	0.396224	0.070*	0.53 (7)
C23	0.101 (5)	0.632 (3)	0.3020 (16)	0.056 (4)	0.53 (7)
H23	0.028557	0.673749	0.271374	0.067*	0.53 (7)
C18A	0.222 (5)	0.547 (4)	0.2737 (15)	0.053 (3)	0.47 (7)
C19A	0.308 (5)	0.459 (4)	0.313 (2)	0.054 (4)	0.47 (7)
H19A	0.355887	0.394865	0.289012	0.065*	0.47 (7)
C20A	0.322 (4)	0.466 (4)	0.388 (2)	0.057 (4)	0.47 (7)
H20A	0.379451	0.406632	0.414826	0.069*	0.47 (7)
C21A	0.250 (6)	0.562 (4)	0.4245 (15)	0.059 (4)	0.47 (7)
H21A	0.259318	0.566251	0.474911	0.071*	0.47 (7)
C22A	0.164 (7)	0.650 (3)	0.385 (2)	0.058 (4)	0.47 (7)
H22A	0.115621	0.714105	0.409183	0.070*	0.47 (7)
C23A	0.150 (6)	0.643 (3)	0.310 (2)	0.057 (4)	0.47 (7)
H23A	0.092056	0.702341	0.283368	0.068*	0.47 (7)
C24	0.2995 (19)	0.4424 (17)	0.1647 (10)	0.053 (4)	
H24A	0.233128	0.370513	0.170301	0.064*	
H24B	0.401590	0.428310	0.190816	0.064*	
C25	0.3281 (16)	0.4609 (14)	0.0851 (9)	0.042 (3)	
H25A	0.405263	0.526314	0.079855	0.051*	
H25B	0.373358	0.386948	0.065500	0.051*	
C26	0.107 (2)	0.6013 (14)	0.0745 (9)	0.049 (4)	
H26A	0.005379	0.618567	0.048646	0.058*	
H26B	0.177715	0.670632	0.068350	0.058*	
C27	0.0796 (19)	0.5836 (16)	0.1541 (9)	0.050 (4)	
H27A	0.040882	0.659632	0.173988	0.059*	
H27B	−0.003443	0.522492	0.159258	0.059*	
S2	0.3197 (4)	0.2483 (4)	−0.1000 (2)	0.0434 (10)	
O4	0.4827 (15)	0.2874 (13)	−0.0758 (7)	0.082 (5)	
O5	0.1897 (18)	0.3143 (12)	−0.0704 (7)	0.076 (4)	
O6	0.3029 (13)	0.1184 (10)	−0.0925 (6)	0.055 (3)	
C28	0.3101 (15)	0.2744 (12)	−0.1926 (7)	0.030 (3)	
C29	0.398 (2)	0.2104 (14)	−0.2397 (9)	0.056 (4)	
H29	0.464748	0.148626	−0.221269	0.067*	
C30	0.393 (2)	0.2318 (19)	−0.3126 (9)	0.069 (5)	
H30	0.454820	0.183365	−0.341942	0.083*	
C31	0.299 (3)	0.3227 (19)	−0.3444 (11)	0.067 (5)	
C32	0.213 (2)	0.3916 (17)	−0.2971 (11)	0.063 (5)	
H32	0.149972	0.455303	−0.315993	0.076*	
C33	0.2151 (19)	0.3708 (15)	−0.2229 (11)	0.051 (4)	
H33	0.154699	0.419715	−0.193132	0.061*	
C34	0.291 (3)	0.347 (3)	−0.4236 (12)	0.108 (9)	
H34A	0.211715	0.408803	−0.434536	0.163*	
H34B	0.261493	0.273269	−0.449234	0.163*	
H34C	0.393878	0.374298	−0.438393	0.163*	

### 4-Phenylpiperazin-1-ium 4-methylbenzenesulfonate (10) Atomic displacement parameters (Å^2^)

**Table:**

	*U*^11^	*U*^22^	*U*^33^	*U*^12^	*U*^13^	*U*^23^
N1	0.035 (6)	0.025 (6)	0.073 (6)	−0.004 (5)	0.003 (5)	0.004 (5)
N2	0.022 (6)	0.028 (7)	0.075 (9)	−0.001 (5)	0.003 (5)	−0.001 (6)
C1	0.057 (8)	0.041 (8)	0.070 (6)	−0.002 (7)	0.006 (6)	0.002 (6)
C2	0.060 (8)	0.043 (8)	0.070 (7)	−0.006 (7)	0.006 (6)	0.000 (7)
C3	0.065 (8)	0.046 (9)	0.071 (7)	−0.008 (8)	0.007 (6)	−0.001 (7)
C4	0.065 (9)	0.049 (9)	0.072 (7)	−0.009 (8)	0.005 (7)	0.000 (7)
C5	0.062 (9)	0.049 (8)	0.073 (7)	−0.007 (8)	0.006 (7)	−0.002 (7)
C6	0.059 (9)	0.045 (8)	0.071 (6)	−0.004 (7)	0.007 (7)	−0.001 (7)
C1A	0.056 (8)	0.041 (7)	0.070 (6)	−0.002 (7)	0.007 (6)	0.002 (6)
C2A	0.058 (8)	0.044 (8)	0.071 (6)	−0.004 (7)	0.007 (6)	0.001 (7)
C3A	0.065 (8)	0.046 (9)	0.071 (7)	−0.009 (8)	0.007 (7)	0.001 (7)
C4A	0.065 (9)	0.050 (8)	0.072 (7)	−0.009 (8)	0.005 (7)	0.001 (7)
C5A	0.061 (9)	0.048 (8)	0.072 (7)	−0.007 (8)	0.006 (7)	−0.001 (7)
C6A	0.059 (9)	0.046 (8)	0.071 (6)	−0.006 (7)	0.007 (7)	0.000 (6)
C7	0.033 (7)	0.041 (10)	0.073 (7)	−0.010 (7)	0.004 (6)	−0.009 (8)
C8	0.051 (9)	0.029 (8)	0.077 (8)	−0.016 (7)	0.004 (7)	0.005 (8)
C9	0.031 (7)	0.036 (9)	0.073 (8)	−0.007 (6)	0.009 (6)	−0.010 (8)
C10	0.038 (9)	0.035 (9)	0.076 (8)	−0.012 (7)	0.012 (7)	0.003 (7)
S1	0.042 (2)	0.029 (2)	0.061 (3)	0.009 (2)	0.0026 (17)	−0.003 (2)
O1	0.046 (7)	0.018 (5)	0.083 (8)	0.002 (5)	0.006 (5)	0.004 (5)
O2	0.114 (10)	0.036 (7)	0.076 (9)	−0.037 (7)	−0.030 (8)	0.007 (7)
O3	0.077 (8)	0.095 (11)	0.078 (8)	0.057 (8)	0.038 (7)	0.019 (8)
C11	0.031 (4)	0.034 (4)	0.043 (4)	0.001 (3)	0.002 (3)	−0.003 (3)
C12	0.037 (9)	0.057 (10)	0.068 (8)	−0.018 (8)	0.000 (7)	0.005 (8)
C13	0.064 (12)	0.068 (13)	0.070 (9)	−0.016 (9)	0.011 (8)	0.013 (9)
C14	0.067 (12)	0.080 (13)	0.062 (9)	0.005 (9)	0.002 (8)	0.000 (9)
C15	0.076 (11)	0.064 (12)	0.067 (8)	−0.009 (10)	−0.017 (8)	−0.012 (9)
C16	0.069 (10)	0.039 (11)	0.069 (8)	−0.017 (8)	−0.013 (8)	−0.002 (7)
C17	0.108 (19)	0.13 (2)	0.068 (10)	0.026 (17)	0.009 (11)	0.013 (12)
N3	0.033 (6)	0.037 (7)	0.067 (5)	−0.005 (5)	−0.002 (5)	0.001 (6)
N4	0.024 (6)	0.023 (6)	0.076 (8)	−0.006 (5)	0.006 (5)	0.005 (6)
C18	0.048 (8)	0.040 (7)	0.067 (6)	−0.001 (7)	−0.002 (5)	0.004 (6)
C19	0.052 (7)	0.044 (8)	0.068 (6)	0.004 (7)	−0.003 (6)	0.002 (7)
C20	0.058 (8)	0.046 (9)	0.069 (6)	0.007 (8)	−0.002 (6)	0.003 (7)
C21	0.058 (9)	0.049 (8)	0.071 (7)	0.008 (8)	−0.002 (6)	0.003 (7)
C22	0.055 (9)	0.048 (8)	0.071 (7)	0.007 (8)	−0.003 (7)	0.000 (7)
C23	0.051 (8)	0.045 (7)	0.071 (6)	0.005 (7)	−0.003 (6)	0.001 (7)
C18A	0.049 (8)	0.041 (7)	0.067 (6)	−0.001 (7)	−0.002 (6)	0.003 (6)
C19A	0.051 (8)	0.043 (8)	0.068 (6)	0.002 (7)	−0.002 (6)	0.003 (7)
C20A	0.057 (8)	0.045 (9)	0.069 (6)	0.008 (8)	−0.003 (6)	0.003 (7)
C21A	0.058 (9)	0.048 (8)	0.071 (7)	0.009 (8)	−0.001 (7)	0.002 (7)
C22A	0.056 (9)	0.047 (8)	0.072 (7)	0.007 (8)	−0.002 (7)	0.000 (7)
C23A	0.053 (9)	0.045 (7)	0.071 (6)	0.003 (8)	−0.003 (7)	0.000 (6)
C24	0.029 (8)	0.052 (10)	0.079 (8)	0.010 (7)	0.000 (7)	0.004 (8)
C25	0.022 (6)	0.022 (8)	0.083 (8)	−0.004 (6)	0.003 (6)	−0.005 (7)
C26	0.052 (9)	0.021 (7)	0.073 (8)	0.012 (7)	0.003 (7)	0.008 (7)
C27	0.047 (4)	0.047 (5)	0.054 (4)	0.005 (3)	0.001 (3)	−0.002 (3)
S2	0.041 (2)	0.030 (2)	0.059 (3)	−0.014 (2)	0.0019 (17)	−0.002 (2)
O4	0.072 (7)	0.087 (11)	0.082 (9)	−0.056 (7)	−0.035 (6)	0.031 (8)
O5	0.107 (10)	0.048 (8)	0.076 (9)	0.008 (7)	0.035 (8)	0.000 (7)
O6	0.058 (7)	0.026 (6)	0.082 (9)	−0.017 (5)	−0.004 (6)	0.009 (6)
C28	0.025 (4)	0.025 (4)	0.039 (4)	−0.008 (3)	0.002 (3)	−0.003 (3)
C29	0.075 (11)	0.033 (10)	0.061 (8)	0.011 (7)	0.011 (8)	−0.004 (7)
C30	0.097 (13)	0.053 (12)	0.059 (8)	0.003 (9)	0.018 (8)	−0.015 (8)
C31	0.069 (12)	0.063 (12)	0.068 (9)	−0.021 (8)	−0.005 (8)	0.005 (8)
C32	0.058 (12)	0.044 (11)	0.087 (9)	0.000 (8)	−0.008 (8)	0.019 (9)
C33	0.035 (8)	0.031 (8)	0.087 (9)	0.005 (6)	0.001 (8)	0.007 (8)
C34	0.11 (2)	0.13 (2)	0.076 (10)	−0.039 (18)	−0.012 (11)	0.022 (13)

### 4-Phenylpiperazin-1-ium 4-methylbenzenesulfonate (10) Geometric parameters (Å, º)

**Table:**

N1—C1A	1.41 (3)	N3—C18	1.43 (3)
N1—C1	1.44 (3)	N3—C27	1.437 (19)
N1—C7	1.437 (19)	N3—C18A	1.44 (3)
N1—C10	1.461 (19)	N3—C24	1.44 (2)
N2—C9	1.467 (18)	N4—C25	1.459 (19)
N2—C8	1.475 (19)	N4—C26	1.473 (18)
N2—H21N	0.8900	N4—H41N	0.8900
N2—H22N	0.8900	N4—H42N	0.8900
C1—C2	1.3900	C18—C19	1.3900
C1—C6	1.3900	C18—C23	1.3900
C2—C3	1.3900	C19—C20	1.3900
C2—H2	0.9300	C19—H19	0.9300
C3—C4	1.3900	C20—C21	1.3900
C3—H3	0.9300	C20—H20	0.9300
C4—C5	1.3900	C21—C22	1.3900
C4—H4	0.9300	C21—H21	0.9300
C5—C6	1.3900	C22—C23	1.3900
C5—H5	0.9300	C22—H22	0.9300
C6—H6	0.9300	C23—H23	0.9300
C1A—C2A	1.3900	C18A—C19A	1.3900
C1A—C6A	1.3900	C18A—C23A	1.3900
C2A—C3A	1.3900	C19A—C20A	1.3900
C2A—H2A	0.9300	C19A—H19A	0.9300
C3A—C4A	1.3900	C20A—C21A	1.3900
C3A—H3A	0.9300	C20A—H20A	0.9300
C4A—C5A	1.3900	C21A—C22A	1.3900
C4A—H4A	0.9300	C21A—H21A	0.9300
C5A—C6A	1.3900	C22A—C23A	1.3900
C5A—H5A	0.9300	C22A—H22A	0.9300
C6A—H6A	0.9300	C23A—H23A	0.9300
C7—C8	1.48 (2)	C24—C25	1.51 (2)
C7—H7A	0.9700	C24—H24A	0.9700
C7—H7B	0.9700	C24—H24B	0.9700
C8—H8A	0.9700	C25—H25A	0.9700
C8—H8B	0.9700	C25—H25B	0.9700
C9—C10	1.51 (2)	C26—C27	1.51 (2)
C9—H9A	0.9700	C26—H26A	0.9700
C9—H9B	0.9700	C26—H26B	0.9700
C10—H10A	0.9700	C27—H27A	0.9700
C10—H10B	0.9700	C27—H27B	0.9700
S1—O2	1.448 (12)	S2—O5	1.430 (13)
S1—O3	1.451 (12)	S2—O6	1.436 (11)
S1—O1	1.462 (11)	S2—O4	1.473 (11)
S1—C11	1.731 (15)	S2—C28	1.728 (14)
C11—C16	1.358 (19)	C28—C29	1.355 (19)
C11—C12	1.43 (2)	C28—C33	1.42 (2)
C12—C13	1.36 (2)	C29—C30	1.36 (2)
C12—H12	0.9300	C29—H29	0.9300
C13—C14	1.34 (3)	C30—C31	1.38 (3)
C13—H13	0.9300	C30—H30	0.9300
C14—C15	1.41 (3)	C31—C32	1.38 (3)
C14—C17	1.49 (3)	C31—C34	1.48 (3)
C15—C16	1.37 (2)	C32—C33	1.39 (3)
C15—H15	0.9300	C32—H32	0.9300
C16—H16	0.9300	C33—H33	0.9300
C17—H17A	0.9600	C34—H34A	0.9600
C17—H17B	0.9600	C34—H34B	0.9600
C17—H17C	0.9600	C34—H34C	0.9600
			
C1A—N1—C7	118.8 (19)	C18—N3—C27	115.7 (18)
C1—N1—C7	116.1 (19)	C27—N3—C18A	119 (2)
C1A—N1—C10	116.9 (19)	C18—N3—C24	118.7 (19)
C1—N1—C10	119.1 (19)	C27—N3—C24	112.9 (14)
C7—N1—C10	111.9 (13)	C18A—N3—C24	115 (2)
C9—N2—C8	110.6 (13)	C25—N4—C26	109.8 (13)
C9—N2—H21N	109.5	C25—N4—H41N	109.7
C8—N2—H21N	109.5	C26—N4—H41N	109.7
C9—N2—H22N	109.5	C25—N4—H42N	109.7
C8—N2—H22N	109.5	C26—N4—H42N	109.7
H21N—N2—H22N	108.1	H41N—N4—H42N	108.2
C2—C1—C6	120.0	C19—C18—C23	120.0
C2—C1—N1	122 (2)	C19—C18—N3	121 (2)
C6—C1—N1	118 (2)	C23—C18—N3	119 (2)
C3—C2—C1	120.0	C20—C19—C18	120.0
C3—C2—H2	120.0	C20—C19—H19	120.0
C1—C2—H2	120.0	C18—C19—H19	120.0
C2—C3—C4	120.0	C21—C20—C19	120.0
C2—C3—H3	120.0	C21—C20—H20	120.0
C4—C3—H3	120.0	C19—C20—H20	120.0
C3—C4—C5	120.0	C22—C21—C20	120.0
C3—C4—H4	120.0	C22—C21—H21	120.0
C5—C4—H4	120.0	C20—C21—H21	120.0
C6—C5—C4	120.0	C21—C22—C23	120.0
C6—C5—H5	120.0	C21—C22—H22	120.0
C4—C5—H5	120.0	C23—C22—H22	120.0
C5—C6—C1	120.0	C22—C23—C18	120.0
C5—C6—H6	120.0	C22—C23—H23	120.0
C1—C6—H6	120.0	C18—C23—H23	120.0
C2A—C1A—C6A	120.0	C19A—C18A—C23A	120.0
C2A—C1A—N1	118 (2)	C19A—C18A—N3	119 (3)
C6A—C1A—N1	122 (2)	C23A—C18A—N3	120 (3)
C1A—C2A—C3A	120.0	C20A—C19A—C18A	120.0
C1A—C2A—H2A	120.0	C20A—C19A—H19A	120.0
C3A—C2A—H2A	120.0	C18A—C19A—H19A	120.0
C4A—C3A—C2A	120.0	C19A—C20A—C21A	120.0
C4A—C3A—H3A	120.0	C19A—C20A—H20A	120.0
C2A—C3A—H3A	120.0	C21A—C20A—H20A	120.0
C3A—C4A—C5A	120.0	C20A—C21A—C22A	120.0
C3A—C4A—H4A	120.0	C20A—C21A—H21A	120.0
C5A—C4A—H4A	120.0	C22A—C21A—H21A	120.0
C6A—C5A—C4A	120.0	C23A—C22A—C21A	120.0
C6A—C5A—H5A	120.0	C23A—C22A—H22A	120.0
C4A—C5A—H5A	120.0	C21A—C22A—H22A	120.0
C5A—C6A—C1A	120.0	C22A—C23A—C18A	120.0
C5A—C6A—H6A	120.0	C22A—C23A—H23A	120.0
C1A—C6A—H6A	120.0	C18A—C23A—H23A	120.0
N1—C7—C8	114.2 (13)	N3—C24—C25	111.9 (14)
N1—C7—H7A	108.7	N3—C24—H24A	109.2
C8—C7—H7A	108.7	C25—C24—H24A	109.2
N1—C7—H7B	108.7	N3—C24—H24B	109.2
C8—C7—H7B	108.7	C25—C24—H24B	109.2
H7A—C7—H7B	107.6	H24A—C24—H24B	107.9
N2—C8—C7	111.8 (13)	N4—C25—C24	111.5 (12)
N2—C8—H8A	109.3	N4—C25—H25A	109.3
C7—C8—H8A	109.3	C24—C25—H25A	109.3
N2—C8—H8B	109.3	N4—C25—H25B	109.3
C7—C8—H8B	109.3	C24—C25—H25B	109.3
H8A—C8—H8B	107.9	H25A—C25—H25B	108.0
N2—C9—C10	110.8 (12)	N4—C26—C27	110.5 (13)
N2—C9—H9A	109.5	N4—C26—H26A	109.6
C10—C9—H9A	109.5	C27—C26—H26A	109.6
N2—C9—H9B	109.5	N4—C26—H26B	109.6
C10—C9—H9B	109.5	C27—C26—H26B	109.6
H9A—C9—H9B	108.1	H26A—C26—H26B	108.1
N1—C10—C9	112.7 (13)	N3—C27—C26	113.3 (13)
N1—C10—H10A	109.1	N3—C27—H27A	108.9
C9—C10—H10A	109.1	C26—C27—H27A	108.9
N1—C10—H10B	109.1	N3—C27—H27B	108.9
C9—C10—H10B	109.1	C26—C27—H27B	108.9
H10A—C10—H10B	107.8	H27A—C27—H27B	107.7
O2—S1—O3	114.3 (10)	O5—S2—O6	112.7 (8)
O2—S1—O1	113.6 (8)	O5—S2—O4	116.2 (9)
O3—S1—O1	111.2 (8)	O6—S2—O4	110.6 (8)
O2—S1—C11	106.6 (7)	O5—S2—C28	106.9 (8)
O3—S1—C11	105.8 (7)	O6—S2—C28	105.0 (7)
O1—S1—C11	104.5 (7)	O4—S2—C28	104.5 (7)
C16—C11—C12	115.3 (15)	C29—C28—C33	116.1 (14)
C16—C11—S1	123.0 (12)	C29—C28—S2	123.0 (12)
C12—C11—S1	121.3 (12)	C33—C28—S2	120.7 (12)
C13—C12—C11	119.8 (17)	C28—C29—C30	123.4 (16)
C13—C12—H12	120.1	C28—C29—H29	118.3
C11—C12—H12	120.1	C30—C29—H29	118.3
C14—C13—C12	124.9 (19)	C29—C30—C31	122.3 (18)
C14—C13—H13	117.5	C29—C30—H30	118.9
C12—C13—H13	117.5	C31—C30—H30	118.9
C13—C14—C15	115.2 (19)	C30—C31—C32	115.3 (19)
C13—C14—C17	124 (2)	C30—C31—C34	123 (2)
C15—C14—C17	120 (2)	C32—C31—C34	121 (2)
C16—C15—C14	121.0 (18)	C31—C32—C33	123.4 (18)
C16—C15—H15	119.5	C31—C32—H32	118.3
C14—C15—H15	119.5	C33—C32—H32	118.3
C11—C16—C15	123.5 (17)	C32—C33—C28	119.5 (16)
C11—C16—H16	118.3	C32—C33—H33	120.2
C15—C16—H16	118.3	C28—C33—H33	120.2
C14—C17—H17A	109.5	C31—C34—H34A	109.5
C14—C17—H17B	109.5	C31—C34—H34B	109.5
H17A—C17—H17B	109.5	H34A—C34—H34B	109.5
C14—C17—H17C	109.5	C31—C34—H34C	109.5
H17A—C17—H17C	109.5	H34A—C34—H34C	109.5
H17B—C17—H17C	109.5	H34B—C34—H34C	109.5
			
C7—N1—C1—C2	−165 (2)	C27—N3—C18—C19	163 (2)
C10—N1—C1—C2	−26 (3)	C24—N3—C18—C19	23 (3)
C7—N1—C1—C6	21 (3)	C27—N3—C18—C23	−23 (3)
C10—N1—C1—C6	159 (2)	C24—N3—C18—C23	−162 (2)
C6—C1—C2—C3	0.0	C23—C18—C19—C20	0.0
N1—C1—C2—C3	−174 (3)	N3—C18—C19—C20	174 (3)
C1—C2—C3—C4	0.0	C18—C19—C20—C21	0.0
C2—C3—C4—C5	0.0	C19—C20—C21—C22	0.0
C3—C4—C5—C6	0.0	C20—C21—C22—C23	0.0
C4—C5—C6—C1	0.0	C21—C22—C23—C18	0.0
C2—C1—C6—C5	0.0	C19—C18—C23—C22	0.0
N1—C1—C6—C5	174 (3)	N3—C18—C23—C22	−174 (3)
C7—N1—C1A—C2A	−147 (2)	C27—N3—C18A—C19A	146 (2)
C10—N1—C1A—C2A	−8 (3)	C24—N3—C18A—C19A	6 (3)
C7—N1—C1A—C6A	39 (3)	C27—N3—C18A—C23A	−41 (3)
C10—N1—C1A—C6A	178 (2)	C24—N3—C18A—C23A	−180 (2)
C6A—C1A—C2A—C3A	0.0	C23A—C18A—C19A—C20A	0.0
N1—C1A—C2A—C3A	−174 (3)	N3—C18A—C19A—C20A	174 (3)
C1A—C2A—C3A—C4A	0.0	C18A—C19A—C20A—C21A	0.0
C2A—C3A—C4A—C5A	0.0	C19A—C20A—C21A—C22A	0.0
C3A—C4A—C5A—C6A	0.0	C20A—C21A—C22A—C23A	0.0
C4A—C5A—C6A—C1A	0.0	C21A—C22A—C23A—C18A	0.0
C2A—C1A—C6A—C5A	0.0	C19A—C18A—C23A—C22A	0.0
N1—C1A—C6A—C5A	174 (3)	N3—C18A—C23A—C22A	−174 (3)
C1A—N1—C7—C8	−168 (2)	C18—N3—C24—C25	−168.7 (18)
C1—N1—C7—C8	−168 (2)	C27—N3—C24—C25	51.1 (17)
C10—N1—C7—C8	50.7 (18)	C18A—N3—C24—C25	−167 (2)
C9—N2—C8—C7	54.6 (17)	C26—N4—C25—C24	57.4 (16)
N1—C7—C8—N2	−52.8 (19)	N3—C24—C25—N4	−54.9 (17)
C8—N2—C9—C10	−55.4 (17)	C25—N4—C26—C27	−56.2 (16)
C1A—N1—C10—C9	167 (2)	C18—N3—C27—C26	167.3 (19)
C1—N1—C10—C9	169 (2)	C18A—N3—C27—C26	169 (2)
C7—N1—C10—C9	−51.2 (17)	C24—N3—C27—C26	−51.3 (19)
N2—C9—C10—N1	54.4 (18)	N4—C26—C27—N3	53.6 (19)
O2—S1—C11—C16	171.6 (14)	O5—S2—C28—C29	−168.7 (13)
O3—S1—C11—C16	−66.3 (15)	O6—S2—C28—C29	−48.8 (14)
O1—S1—C11—C16	51.1 (15)	O4—S2—C28—C29	67.7 (14)
O2—S1—C11—C12	−16.1 (15)	O5—S2—C28—C33	15.2 (14)
O3—S1—C11—C12	105.9 (14)	O6—S2—C28—C33	135.1 (12)
O1—S1—C11—C12	−136.7 (13)	O4—S2—C28—C33	−108.5 (13)
C16—C11—C12—C13	−5 (2)	C33—C28—C29—C30	−3 (2)
S1—C11—C12—C13	−178.3 (14)	S2—C28—C29—C30	−178.9 (14)
C11—C12—C13—C14	2 (3)	C28—C29—C30—C31	1 (3)
C12—C13—C14—C15	2 (3)	C29—C30—C31—C32	1 (3)
C12—C13—C14—C17	180 (2)	C29—C30—C31—C34	−179.4 (19)
C13—C14—C15—C16	−2 (3)	C30—C31—C32—C33	−1 (3)
C17—C14—C15—C16	−179.9 (19)	C34—C31—C32—C33	178.8 (19)
C12—C11—C16—C15	6 (3)	C31—C32—C33—C28	0 (3)
S1—C11—C16—C15	178.4 (15)	C29—C28—C33—C32	2 (2)
C14—C15—C16—C11	−2 (3)	S2—C28—C33—C32	178.3 (13)

### 4-Phenylpiperazin-1-ium 4-methylbenzenesulfonate (10) Hydrogen-bond geometry (Å, º)

**Table:**

*D*—H···*A*	*D*—H	H···*A*	*D*···*A*	*D*—H···*A*
N2—H21*N*···O6	0.89	2.07	2.884 (18)	151
N2—H22*N*···O4^i^	0.89	1.92	2.774 (17)	161
C9—H9*B*···O1^ii^	0.97	2.64	3.534 (19)	154
N4—H41*N*···O3^ii^	0.89	1.92	2.788 (16)	163
N4—H42*N*···O1	0.89	2.09	2.890 (18)	149
N4—H42*N*···O5	0.89	2.43	2.865 (18)	111
C25—H25*A*···O6^iii^	0.97	2.63	3.520 (18)	153

Symmetry codes: (i) −*x*+1, *y*−1/2, −*z*; (ii) −*x*, *y*−1/2, −*z*; (iii) −*x*+1, *y*+1/2, −*z*.

### 4-Phenylpiperazin-1-ium 4-carboxy-2,3-dihydroxybutanoate monohydrate (11) Crystal data

**Table:**

C_10_H_15_N_2_^+^·C_4_H_5_O_6_^−^·H_2_O	*D*_x_ = 1.367 Mg m^−^^3^
*M**_r_* = 330.33	Mo *K*α radiation, λ = 0.71073 Å
Orthorhombic, *P*2_1_2_1_2_1_	Cell parameters from 3790 reflections
*a* = 7.1185 (7) Å	θ = 2.9–27.8°
*b* = 7.5255 (8) Å	µ = 0.11 mm^−^^1^
*c* = 29.955 (3) Å	*T* = 293 K
*V* = 1604.7 (3) Å^3^	Prism, colourless
*Z* = 4	0.42 × 0.32 × 0.24 mm
*F*(000) = 704	

### 4-Phenylpiperazin-1-ium 4-carboxy-2,3-dihydroxybutanoate monohydrate (11) Data collection

**Table:**

Oxford Diffraction Xcalibur with Sapphire CCD diffractometer	2808 reflections with *I* > 2σ(*I*)
Radiation source: Enhance (Mo) X-ray Source	*R*_int_ = 0.019
Rotation method data acquisition using ω scans.	θ_max_ = 27.8°, θ_min_ = 2.9°
Absorption correction: multi-scan (CrysalisRED; Oxford Diffraction, 2007)	*h* = −9→9
*T*_min_ = 0.883, *T*_max_ = 1.000	*k* = −6→9
6773 measured reflections	*l* = −36→38
3354 independent reflections	

### 4-Phenylpiperazin-1-ium 4-carboxy-2,3-dihydroxybutanoate monohydrate (11) Refinement

**Table:**

Refinement on *F*^2^	Secondary atom site location: difference Fourier map
Least-squares matrix: full	Hydrogen site location: mixed
*R*[*F*^2^ > 2σ(*F*^2^)] = 0.045	H atoms treated by a mixture of independent and constrained refinement
*wR*(*F*^2^) = 0.100	*w* = 1/[σ^2^(*F*_o_^2^) + (0.0359*P*)^2^ + 0.5036*P*] where *P* = (*F*_o_^2^ + 2*F*_c_^2^)/3
*S* = 1.09	(Δ/σ)_max_ < 0.001
3354 reflections	Δρ_max_ = 0.20 e Å^−^^3^
260 parameters	Δρ_min_ = −0.16 e Å^−^^3^
211 restraints	Absolute structure: Flack *x* determined using 912 quotients [(*I*^+^)-(*I*^-^)]/[(*I*^+^)+(*I*^-^)] (Parsons *et al.*, 2013)
Primary atom site location: dual	Absolute structure parameter: −0.2 (5)

### 4-Phenylpiperazin-1-ium 4-carboxy-2,3-dihydroxybutanoate monohydrate (11) Special details

**Table:**

Geometry. All esds (except the esd in the dihedral angle between two l.s. planes) are estimated using the full covariance matrix. The cell esds are taken into account individually in the estimation of esds in distances, angles and torsion angles; correlations between esds in cell parameters are only used when they are defined by crystal symmetry. An approximate (isotropic) treatment of cell esds is used for estimating esds involving l.s. planes.

### 4-Phenylpiperazin-1-ium 4-carboxy-2,3-dihydroxybutanoate monohydrate (11) Fractional atomic coordinates and isotropic or equivalent isotropic displacement parameters (Å^2^)

**Table:**

	*x*	*y*	*z*	*U*_iso_*/*U*_eq_	Occ. (<1)
N1	0.2138 (4)	0.3376 (4)	0.69293 (7)	0.0428 (6)	
N2	0.0481 (4)	0.3028 (5)	0.60649 (9)	0.0515 (8)	
H21	0.057 (6)	0.189 (3)	0.6004 (13)	0.062*	
H22	−0.007 (5)	0.338 (5)	0.5825 (9)	0.062*	
C1	0.3025 (8)	0.3071 (7)	0.7352 (2)	0.0393 (16)	0.611 (13)
C2	0.4926 (8)	0.2687 (11)	0.73967 (16)	0.0489 (16)	0.611 (13)
H2	0.566167	0.250879	0.714417	0.059*	0.611 (13)
C3	0.5726 (8)	0.2568 (12)	0.78189 (19)	0.0596 (18)	0.611 (13)
H3	0.699769	0.231094	0.784885	0.071*	0.611 (13)
C4	0.4626 (12)	0.2834 (8)	0.81965 (16)	0.0629 (19)	0.611 (13)
H4	0.516140	0.275436	0.847893	0.076*	0.611 (13)
C5	0.2725 (11)	0.3218 (11)	0.8152 (2)	0.0565 (19)	0.611 (13)
H5	0.198908	0.339564	0.840434	0.068*	0.611 (13)
C6	0.1925 (8)	0.3336 (10)	0.7730 (3)	0.0469 (17)	0.611 (13)
H6	0.065303	0.359351	0.769967	0.056*	0.611 (13)
C1A	0.3005 (14)	0.3277 (13)	0.7366 (4)	0.044 (2)	0.389 (13)
C2A	0.4913 (13)	0.3634 (19)	0.7408 (3)	0.054 (2)	0.389 (13)
H2A	0.561166	0.395753	0.715828	0.065*	0.389 (13)
C3A	0.5777 (13)	0.3508 (18)	0.7823 (3)	0.058 (2)	0.389 (13)
H3A	0.705291	0.374729	0.785092	0.069*	0.389 (13)
C4A	0.4732 (18)	0.3026 (13)	0.8196 (3)	0.060 (3)	0.389 (13)
H4A	0.531021	0.294126	0.847328	0.073*	0.389 (13)
C5A	0.2825 (18)	0.2669 (16)	0.8154 (4)	0.052 (2)	0.389 (13)
H5A	0.212626	0.234547	0.840300	0.062*	0.389 (13)
C6A	0.1961 (13)	0.2795 (15)	0.7739 (5)	0.044 (2)	0.389 (13)
H6A	0.068497	0.255570	0.771037	0.053*	0.389 (13)
C7	0.0322 (4)	0.2513 (6)	0.68665 (10)	0.0600 (10)	
H7A	−0.044378	0.269546	0.713032	0.072*	
H7B	0.051046	0.124480	0.683019	0.072*	
C8	−0.0694 (5)	0.3230 (6)	0.64654 (11)	0.0615 (11)	
H8A	−0.186632	0.259277	0.642510	0.074*	
H8B	−0.098445	0.447573	0.651123	0.074*	
C9	0.2345 (5)	0.3864 (6)	0.61270 (11)	0.0626 (11)	
H9A	0.219400	0.513976	0.615495	0.075*	
H9B	0.311852	0.363336	0.586672	0.075*	
C10	0.3310 (4)	0.3153 (6)	0.65361 (9)	0.0516 (9)	
H10A	0.358743	0.190224	0.649445	0.062*	
H10B	0.448994	0.377545	0.657941	0.062*	
O1	0.3807 (3)	0.8465 (3)	0.42742 (7)	0.0391 (5)	
O2	0.0905 (3)	0.9424 (3)	0.44164 (9)	0.0536 (6)	
O3	−0.0601 (3)	0.6256 (3)	0.44303 (8)	0.0403 (5)	
H3O	−0.097 (5)	0.721 (3)	0.4540 (10)	0.048*	
O4	0.1952 (3)	0.6293 (3)	0.51830 (6)	0.0405 (5)	
H4O	0.091 (3)	0.613 (5)	0.5267 (11)	0.049*	
O5	0.0826 (3)	0.2841 (3)	0.51284 (7)	0.0513 (6)	
O6	0.1893 (3)	0.2612 (2)	0.44300 (6)	0.0369 (5)	
H6O	0.153 (4)	0.156 (3)	0.4444 (11)	0.044*	
C11	0.2097 (4)	0.8232 (3)	0.43575 (9)	0.0323 (6)	
C12	0.1380 (3)	0.6322 (3)	0.43867 (9)	0.0277 (5)	
H12	0.173538	0.569709	0.411225	0.033*	
C13	0.2286 (4)	0.5366 (3)	0.47836 (8)	0.0284 (6)	
H13	0.364670	0.533612	0.473374	0.034*	
C14	0.1588 (4)	0.3466 (4)	0.48051 (8)	0.0311 (6)	
O7	−0.3916 (4)	0.5658 (4)	0.39633 (9)	0.0629 (7)	
H71O	−0.286 (4)	0.586 (6)	0.4077 (13)	0.075*	
H72O	−0.462 (5)	0.649 (4)	0.4054 (14)	0.075*	

### 4-Phenylpiperazin-1-ium 4-carboxy-2,3-dihydroxybutanoate monohydrate (11) Atomic displacement parameters (Å^2^)

**Table:**

	*U*^11^	*U*^22^	*U*^33^	*U*^12^	*U*^13^	*U*^23^
N1	0.0367 (13)	0.0643 (16)	0.0275 (11)	−0.0036 (13)	−0.0040 (10)	0.0023 (12)
N2	0.0418 (14)	0.083 (2)	0.0299 (13)	−0.0080 (17)	−0.0080 (11)	0.0064 (15)
C1	0.042 (3)	0.047 (3)	0.029 (3)	0.000 (3)	−0.004 (3)	−0.009 (3)
C2	0.044 (3)	0.067 (4)	0.037 (2)	0.012 (3)	−0.003 (2)	0.001 (3)
C3	0.056 (3)	0.075 (5)	0.048 (3)	0.012 (4)	−0.017 (2)	0.003 (4)
C4	0.074 (4)	0.079 (4)	0.036 (3)	0.007 (4)	−0.016 (3)	0.002 (3)
C5	0.067 (3)	0.069 (5)	0.033 (3)	0.005 (4)	−0.002 (3)	−0.002 (3)
C6	0.048 (3)	0.056 (4)	0.037 (3)	0.009 (3)	−0.002 (2)	−0.005 (3)
C1A	0.044 (4)	0.051 (5)	0.036 (4)	0.003 (4)	−0.005 (4)	0.010 (4)
C2A	0.050 (4)	0.072 (5)	0.039 (4)	−0.001 (5)	−0.003 (3)	0.006 (4)
C3A	0.052 (4)	0.078 (6)	0.043 (4)	0.000 (5)	−0.016 (3)	−0.001 (5)
C4A	0.068 (5)	0.078 (5)	0.036 (4)	0.001 (5)	−0.017 (4)	−0.002 (4)
C5A	0.065 (4)	0.058 (5)	0.032 (4)	0.005 (4)	0.001 (4)	0.001 (4)
C6A	0.048 (4)	0.048 (5)	0.036 (4)	0.002 (4)	−0.001 (3)	0.003 (4)
C7	0.0388 (17)	0.108 (3)	0.0335 (16)	−0.015 (2)	0.0016 (13)	0.0005 (19)
C8	0.0405 (18)	0.101 (3)	0.0431 (18)	0.007 (2)	−0.0058 (14)	−0.010 (2)
C9	0.051 (2)	0.097 (3)	0.0398 (17)	−0.023 (2)	−0.0084 (15)	0.0173 (19)
C10	0.0357 (16)	0.085 (3)	0.0343 (15)	−0.0079 (18)	−0.0012 (12)	0.0073 (17)
O1	0.0305 (10)	0.0412 (11)	0.0458 (11)	−0.0059 (9)	0.0039 (9)	−0.0013 (10)
O2	0.0379 (12)	0.0274 (10)	0.0954 (18)	0.0020 (9)	0.0058 (12)	−0.0045 (12)
O3	0.0255 (10)	0.0319 (10)	0.0635 (14)	−0.0007 (9)	−0.0047 (10)	−0.0087 (11)
O4	0.0355 (11)	0.0538 (12)	0.0323 (10)	−0.0080 (11)	0.0028 (9)	−0.0162 (10)
O5	0.0641 (15)	0.0544 (13)	0.0354 (11)	−0.0131 (12)	0.0138 (10)	0.0052 (10)
O6	0.0472 (12)	0.0257 (9)	0.0379 (10)	−0.0018 (9)	0.0084 (9)	−0.0025 (9)
C11	0.0325 (14)	0.0308 (14)	0.0336 (14)	−0.0019 (13)	−0.0026 (12)	−0.0016 (12)
C12	0.0231 (12)	0.0276 (13)	0.0326 (13)	0.0017 (11)	−0.0013 (11)	−0.0044 (12)
C13	0.0249 (13)	0.0353 (14)	0.0248 (12)	−0.0001 (12)	0.0015 (11)	−0.0045 (11)
C14	0.0291 (14)	0.0349 (14)	0.0293 (13)	0.0013 (12)	0.0002 (11)	0.0009 (12)
O7	0.0411 (13)	0.0784 (19)	0.0691 (16)	0.0049 (14)	−0.0092 (12)	−0.0320 (15)

### 4-Phenylpiperazin-1-ium 4-carboxy-2,3-dihydroxybutanoate monohydrate (11) Geometric parameters (Å, º)

**Table:**

N1—C1	1.434 (6)	C5A—H5A	0.9300
N1—C1A	1.448 (9)	C6A—H6A	0.9300
N1—C10	1.453 (4)	C7—C8	1.502 (5)
N1—C7	1.459 (4)	C7—H7A	0.9700
N2—C8	1.470 (4)	C7—H7B	0.9700
N2—C9	1.480 (4)	C8—H8A	0.9700
N2—H21	0.88 (2)	C8—H8B	0.9700
N2—H22	0.86 (2)	C9—C10	1.503 (4)
C1—C2	1.3900	C9—H9A	0.9700
C1—C6	1.3900	C9—H9B	0.9700
C2—C3	1.3900	C10—H10A	0.9700
C2—H2	0.9300	C10—H10B	0.9700
C3—C4	1.3900	O1—C11	1.255 (3)
C3—H3	0.9300	O2—C11	1.247 (3)
C4—C5	1.3900	O3—C12	1.417 (3)
C4—H4	0.9300	O3—H3O	0.83 (2)
C5—C6	1.3900	O4—C13	1.405 (3)
C5—H5	0.9300	O4—H4O	0.79 (2)
C6—H6	0.9300	O5—C14	1.205 (3)
C1A—C2A	1.3900	O6—C14	1.312 (3)
C1A—C6A	1.3900	O6—H6O	0.83 (2)
C2A—C3A	1.3900	C11—C12	1.528 (4)
C2A—H2A	0.9300	C12—C13	1.532 (4)
C3A—C4A	1.3900	C12—H12	0.9800
C3A—H3A	0.9300	C13—C14	1.516 (4)
C4A—C5A	1.3900	C13—H13	0.9800
C4A—H4A	0.9300	O7—H71O	0.84 (2)
C5A—C6A	1.3900	O7—H72O	0.85 (2)
			
C1—N1—C10	116.4 (3)	N1—C7—C8	111.7 (3)
C1A—N1—C10	118.8 (5)	N1—C7—H7A	109.3
C1—N1—C7	115.6 (3)	C8—C7—H7A	109.3
C1A—N1—C7	118.1 (5)	N1—C7—H7B	109.3
C10—N1—C7	110.7 (2)	C8—C7—H7B	109.3
C8—N2—C9	111.3 (3)	H7A—C7—H7B	107.9
C8—N2—H21	108 (3)	N2—C8—C7	110.0 (3)
C9—N2—H21	112 (3)	N2—C8—H8A	109.7
C8—N2—H22	113 (3)	C7—C8—H8A	109.7
C9—N2—H22	112 (3)	N2—C8—H8B	109.7
H21—N2—H22	99 (4)	C7—C8—H8B	109.7
C2—C1—C6	120.0	H8A—C8—H8B	108.2
C2—C1—N1	123.2 (5)	N2—C9—C10	111.2 (3)
C6—C1—N1	116.6 (5)	N2—C9—H9A	109.4
C1—C2—C3	120.0	C10—C9—H9A	109.4
C1—C2—H2	120.0	N2—C9—H9B	109.4
C3—C2—H2	120.0	C10—C9—H9B	109.4
C4—C3—C2	120.0	H9A—C9—H9B	108.0
C4—C3—H3	120.0	N1—C10—C9	110.9 (3)
C2—C3—H3	120.0	N1—C10—H10A	109.5
C3—C4—C5	120.0	C9—C10—H10A	109.5
C3—C4—H4	120.0	N1—C10—H10B	109.5
C5—C4—H4	120.0	C9—C10—H10B	109.5
C4—C5—C6	120.0	H10A—C10—H10B	108.0
C4—C5—H5	120.0	C12—O3—H3O	109 (2)
C6—C5—H5	120.0	C13—O4—H4O	111 (3)
C5—C6—C1	120.0	C14—O6—H6O	112 (2)
C5—C6—H6	120.0	O2—C11—O1	126.0 (3)
C1—C6—H6	120.0	O2—C11—C12	116.2 (2)
C2A—C1A—C6A	120.0	O1—C11—C12	117.8 (2)
C2A—C1A—N1	119.3 (7)	O3—C12—C11	111.7 (2)
C6A—C1A—N1	120.7 (7)	O3—C12—C13	109.3 (2)
C1A—C2A—C3A	120.0	C11—C12—C13	110.2 (2)
C1A—C2A—H2A	120.0	O3—C12—H12	108.5
C3A—C2A—H2A	120.0	C11—C12—H12	108.5
C4A—C3A—C2A	120.0	C13—C12—H12	108.5
C4A—C3A—H3A	120.0	O4—C13—C14	112.1 (2)
C2A—C3A—H3A	120.0	O4—C13—C12	110.9 (2)
C3A—C4A—C5A	120.0	C14—C13—C12	109.7 (2)
C3A—C4A—H4A	120.0	O4—C13—H13	108.0
C5A—C4A—H4A	120.0	C14—C13—H13	108.0
C4A—C5A—C6A	120.0	C12—C13—H13	108.0
C4A—C5A—H5A	120.0	O5—C14—O6	124.9 (3)
C6A—C5A—H5A	120.0	O5—C14—C13	123.3 (2)
C5A—C6A—C1A	120.0	O6—C14—C13	111.8 (2)
C5A—C6A—H6A	120.0	H71O—O7—H72O	105 (4)
C1A—C6A—H6A	120.0		
			
C10—N1—C1—C2	3.8 (6)	N1—C1A—C6A—C5A	178.6 (8)
C7—N1—C1—C2	136.3 (5)	C1—N1—C7—C8	166.9 (4)
C10—N1—C1—C6	178.1 (4)	C1A—N1—C7—C8	160.3 (5)
C7—N1—C1—C6	−49.5 (5)	C10—N1—C7—C8	−58.0 (4)
C6—C1—C2—C3	0.0	C9—N2—C8—C7	−54.9 (5)
N1—C1—C2—C3	174.0 (5)	N1—C7—C8—N2	56.7 (5)
C1—C2—C3—C4	0.0	C8—N2—C9—C10	54.9 (5)
C2—C3—C4—C5	0.0	C1—N1—C10—C9	−168.4 (4)
C3—C4—C5—C6	0.0	C1A—N1—C10—C9	−161.7 (5)
C4—C5—C6—C1	0.0	C7—N1—C10—C9	56.8 (4)
C2—C1—C6—C5	0.0	N2—C9—C10—N1	−55.5 (4)
N1—C1—C6—C5	−174.4 (5)	O2—C11—C12—O3	6.3 (3)
C10—N1—C1A—C2A	23.7 (8)	O1—C11—C12—O3	−173.2 (2)
C7—N1—C1A—C2A	162.3 (6)	O2—C11—C12—C13	−115.4 (3)
C10—N1—C1A—C6A	−154.9 (6)	O1—C11—C12—C13	65.1 (3)
C7—N1—C1A—C6A	−16.3 (9)	O3—C12—C13—O4	−66.8 (3)
C6A—C1A—C2A—C3A	0.0	C11—C12—C13—O4	56.4 (3)
N1—C1A—C2A—C3A	−178.6 (8)	O3—C12—C13—C14	57.7 (3)
C1A—C2A—C3A—C4A	0.0	C11—C12—C13—C14	−179.2 (2)
C2A—C3A—C4A—C5A	0.0	O4—C13—C14—O5	0.4 (4)
C3A—C4A—C5A—C6A	0.0	C12—C13—C14—O5	−123.3 (3)
C4A—C5A—C6A—C1A	0.0	O4—C13—C14—O6	179.5 (2)
C2A—C1A—C6A—C5A	0.0	C12—C13—C14—O6	55.8 (3)

### 4-Phenylpiperazin-1-ium 4-carboxy-2,3-dihydroxybutanoate monohydrate (11) Hydrogen-bond geometry (Å, º)

**Table:**

*D*—H···*A*	*D*—H	H···*A*	*D*···*A*	*D*—H···*A*
N2—H21···O7^i^	0.88 (2)	1.95 (2)	2.808 (5)	164 (4)
N2—H22···O1^ii^	0.86 (2)	2.52 (3)	3.069 (4)	122 (3)
N2—H22···O5	0.86 (2)	2.22 (3)	2.820 (3)	127 (3)
N2—H22···O6^iii^	0.86 (2)	2.41 (3)	2.992 (3)	125 (3)
C9—H9*B*···O2^iv^	0.97	2.61	3.276 (4)	126
O3—H3*O*···O2	0.83 (2)	2.17 (3)	2.614 (3)	114 (3)
O3—H3*O*···O4^ii^	0.83 (2)	2.04 (2)	2.789 (3)	150 (3)
O4—H4*O*···O1^ii^	0.79 (2)	2.06 (3)	2.773 (3)	151 (3)
O6—H6*O*···O2^v^	0.83 (2)	1.67 (2)	2.501 (3)	174 (3)
O7—H71*O*···O3	0.84 (2)	1.95 (2)	2.780 (3)	171 (4)
O7—H72*O*···O1^vi^	0.85 (2)	1.97 (2)	2.821 (3)	178 (4)

Symmetry codes: (i) *x*+1/2, −*y*+1/2, −*z*+1; (ii) *x*−1/2, −*y*+3/2, −*z*+1; (iii) *x*−1/2, −*y*+1/2, −*z*+1; (iv) *x*+1/2, −*y*+3/2, −*z*+1; (v) *x*, *y*−1, *z*; (vi) *x*−1, *y*, *z*.

### 4-Phenylpiperazin-1-ium fumarate (12) Crystal data

**Table:**

C_10_H_15_N_2_^+^·C_4_H_3_O_4_^−^	*D*_x_ = 1.287 Mg m^−^^3^
*M**_r_* = 278.30	Mo *K*α radiation, λ = 0.71073 Å
Orthorhombic, *P**c**a*2_1_	Cell parameters from 6248 reflections
*a* = 26.702 (1) Å	θ = 2.7–27.8°
*b* = 7.9626 (3) Å	µ = 0.10 mm^−^^1^
*c* = 6.7571 (3) Å	*T* = 293 K
*V* = 1436.68 (10) Å^3^	Prism, light brown
*Z* = 4	0.48 × 0.44 × 0.40 mm
*F*(000) = 592	

### 4-Phenylpiperazin-1-ium fumarate (12) Data collection

**Table:**

Oxford Diffraction Xcalibur with Sapphire CCD diffractometer	2770 reflections with *I* > 2σ(*I*)
Radiation source: Enhance (Mo) X-ray Source	*R*_int_ = 0.018
Rotation method data acquisition using ω scans.	θ_max_ = 27.9°, θ_min_ = 3.0°
Absorption correction: multi-scan (CrysalisRED; Oxford Diffraction, 2007)	*h* = −33→33
*T*_min_ = 0.894, *T*_max_ = 1.000	*k* = −10→5
9534 measured reflections	*l* = −8→8
3127 independent reflections	

### 4-Phenylpiperazin-1-ium fumarate (12) Refinement

**Table:**

Refinement on *F*^2^	Hydrogen site location: mixed
Least-squares matrix: full	H atoms treated by a mixture of independent and constrained refinement
*R*[*F*^2^ > 2σ(*F*^2^)] = 0.034	*w* = 1/[σ^2^(*F*_o_^2^) + (0.0296*P*)^2^ + 0.3161*P*] where *P* = (*F*_o_^2^ + 2*F*_c_^2^)/3
*wR*(*F*^2^) = 0.077	(Δ/σ)_max_ < 0.001
*S* = 1.06	Δρ_max_ = 0.17 e Å^−^^3^
3127 reflections	Δρ_min_ = −0.13 e Å^−^^3^
191 parameters	Extinction correction: SHELXL-2018/3 (Sheldrick 2018), Fc^*^=kFc[1+0.001xFc^2^λ^3^/sin(2θ)]^-1/4^
4 restraints	Extinction coefficient: 0.024 (5)
Primary atom site location: dual	Absolute structure: Flack *x* determined using 1130 quotients [(*I*^+^)-(*I*^-^)]/[(*I*^+^)+(*I*^-^)] (Parsons *et al.*, 2013)
Secondary atom site location: difference Fourier map	Absolute structure parameter: 0.3 (3)

### 4-Phenylpiperazin-1-ium fumarate (12) Special details

**Table:**

Geometry. All esds (except the esd in the dihedral angle between two l.s. planes) are estimated using the full covariance matrix. The cell esds are taken into account individually in the estimation of esds in distances, angles and torsion angles; correlations between esds in cell parameters are only used when they are defined by crystal symmetry. An approximate (isotropic) treatment of cell esds is used for estimating esds involving l.s. planes.

### 4-Phenylpiperazin-1-ium fumarate (12) Fractional atomic coordinates and isotropic or equivalent isotropic displacement parameters (Å^2^)

**Table:**

	*x*	*y*	*z*	*U*_iso_*/*U*_eq_	
N1	0.07139 (6)	0.0974 (2)	0.4560 (3)	0.0396 (4)	
N2	0.15289 (7)	0.1889 (2)	0.2018 (3)	0.0359 (4)	
H21	0.1639 (9)	0.258 (3)	0.111 (3)	0.043*	
H22	0.1758 (8)	0.112 (3)	0.231 (4)	0.043*	
C1	0.02675 (7)	0.1920 (3)	0.4495 (3)	0.0341 (5)	
C2	−0.01118 (8)	0.1565 (3)	0.3155 (4)	0.0460 (5)	
H2	−0.006524	0.072842	0.221209	0.055*	
C3	−0.05581 (9)	0.2442 (3)	0.3210 (5)	0.0554 (6)	
H3	−0.080837	0.217775	0.230469	0.066*	
C4	−0.06407 (10)	0.3685 (3)	0.4555 (5)	0.0581 (8)	
H4	−0.094144	0.427343	0.457231	0.070*	
C5	−0.02681 (11)	0.4041 (4)	0.5880 (5)	0.0605 (8)	
H5	−0.031873	0.488166	0.681492	0.073*	
C6	0.01794 (9)	0.3187 (3)	0.5862 (4)	0.0493 (6)	
H6	0.042619	0.346194	0.677785	0.059*	
C7	0.11662 (9)	0.1763 (4)	0.5335 (4)	0.0516 (7)	
H7A	0.140074	0.089729	0.573846	0.062*	
H7B	0.108145	0.242129	0.649545	0.062*	
C8	0.14116 (9)	0.2881 (3)	0.3825 (4)	0.0453 (6)	
H8A	0.118887	0.380029	0.348976	0.054*	
H8B	0.171722	0.335098	0.436782	0.054*	
C9	0.10846 (8)	0.0982 (3)	0.1241 (3)	0.0406 (5)	
H9A	0.118462	0.025512	0.015935	0.049*	
H9B	0.084244	0.178364	0.073747	0.049*	
C10	0.08503 (8)	−0.0048 (3)	0.2859 (4)	0.0458 (6)	
H10A	0.055321	−0.060037	0.234969	0.055*	
H10B	0.108400	−0.091175	0.327448	0.055*	
O1	0.28279 (5)	0.92767 (16)	−0.2019 (3)	0.0457 (4)	
O2	0.20615 (5)	1.03469 (16)	−0.1699 (3)	0.0403 (4)	
H2O	0.2217 (8)	1.136 (3)	−0.190 (4)	0.048*	
O3	0.24248 (5)	0.31442 (15)	−0.2281 (3)	0.0395 (4)	
O4	0.17409 (5)	0.41963 (17)	−0.0874 (3)	0.0411 (4)	
C11	0.23766 (7)	0.9127 (2)	−0.1764 (3)	0.0298 (4)	
C12	0.21444 (7)	0.7446 (2)	−0.1522 (4)	0.0324 (5)	
H12	0.181218	0.738436	−0.111463	0.039*	
C13	0.23863 (7)	0.6049 (2)	−0.1857 (3)	0.0297 (4)	
H13	0.271921	0.611739	−0.225248	0.036*	
C14	0.21578 (7)	0.4351 (2)	−0.1639 (3)	0.0283 (4)	

### 4-Phenylpiperazin-1-ium fumarate (12) Atomic displacement parameters (Å^2^)

**Table:**

	*U*^11^	*U*^22^	*U*^33^	*U*^12^	*U*^13^	*U*^23^
N1	0.0316 (9)	0.0426 (10)	0.0447 (11)	0.0009 (8)	0.0062 (8)	0.0066 (9)
N2	0.0258 (9)	0.0317 (9)	0.0504 (12)	0.0015 (7)	0.0070 (8)	0.0071 (9)
C1	0.0292 (10)	0.0343 (10)	0.0389 (11)	−0.0044 (8)	0.0063 (9)	0.0047 (10)
C2	0.0385 (11)	0.0495 (12)	0.0502 (14)	−0.0029 (10)	0.0001 (12)	−0.0091 (13)
C3	0.0355 (11)	0.0636 (15)	0.0670 (17)	−0.0042 (11)	−0.0095 (14)	0.0038 (16)
C4	0.0364 (13)	0.0466 (14)	0.091 (2)	0.0085 (11)	0.0081 (15)	0.0066 (16)
C5	0.0538 (16)	0.0474 (15)	0.080 (2)	0.0057 (12)	0.0095 (15)	−0.0165 (15)
C6	0.0423 (13)	0.0489 (14)	0.0567 (16)	−0.0027 (11)	−0.0030 (12)	−0.0138 (13)
C7	0.0339 (13)	0.0817 (19)	0.0391 (13)	0.0062 (13)	−0.0057 (10)	0.0027 (14)
C8	0.0279 (10)	0.0477 (13)	0.0604 (16)	−0.0049 (10)	−0.0010 (10)	−0.0136 (12)
C9	0.0338 (12)	0.0459 (13)	0.0419 (13)	−0.0004 (10)	0.0034 (10)	−0.0075 (11)
C10	0.0368 (11)	0.0295 (10)	0.0709 (17)	−0.0033 (9)	0.0130 (12)	−0.0028 (12)
O1	0.0309 (7)	0.0259 (7)	0.0802 (13)	−0.0035 (6)	0.0087 (9)	0.0026 (9)
O2	0.0367 (7)	0.0188 (6)	0.0654 (11)	0.0011 (6)	0.0051 (8)	0.0038 (8)
O3	0.0374 (8)	0.0208 (6)	0.0604 (11)	0.0016 (6)	0.0103 (7)	−0.0015 (7)
O4	0.0305 (8)	0.0258 (7)	0.0671 (11)	0.0008 (6)	0.0107 (7)	0.0088 (7)
C11	0.0332 (10)	0.0224 (9)	0.0338 (11)	−0.0009 (7)	0.0011 (9)	0.0004 (9)
C12	0.0297 (9)	0.0231 (8)	0.0443 (12)	−0.0037 (8)	0.0045 (9)	0.0019 (9)
C13	0.0297 (9)	0.0224 (8)	0.0369 (11)	−0.0028 (7)	0.0024 (9)	0.0029 (9)
C14	0.0283 (9)	0.0201 (8)	0.0365 (10)	0.0003 (7)	−0.0024 (9)	0.0033 (9)

### 4-Phenylpiperazin-1-ium fumarate (12) Geometric parameters (Å, º)

**Table:**

N1—C1	1.411 (3)	C7—H7A	0.9700
N1—C10	1.455 (3)	C7—H7B	0.9700
N1—C7	1.459 (3)	C8—H8A	0.9700
N2—C9	1.485 (3)	C8—H8B	0.9700
N2—C8	1.487 (3)	C9—C10	1.504 (3)
N2—H21	0.87 (2)	C9—H9A	0.9700
N2—H22	0.89 (2)	C9—H9B	0.9700
C1—C2	1.388 (3)	C10—H10A	0.9700
C1—C6	1.388 (3)	C10—H10B	0.9700
C2—C3	1.382 (3)	O1—C11	1.223 (2)
C2—H2	0.9300	O2—C11	1.286 (2)
C3—C4	1.361 (4)	O2—H2O	0.921 (19)
C3—H3	0.9300	O3—C14	1.273 (2)
C4—C5	1.368 (4)	O4—C14	1.234 (2)
C4—H4	0.9300	C11—C12	1.485 (2)
C5—C6	1.375 (4)	C12—C13	1.306 (3)
C5—H5	0.9300	C12—H12	0.9300
C6—H6	0.9300	C13—C14	1.490 (2)
C7—C8	1.505 (4)	C13—H13	0.9300
			
C1—N1—C10	119.1 (2)	H7A—C7—H7B	107.9
C1—N1—C7	118.8 (2)	N2—C8—C7	109.53 (19)
C10—N1—C7	108.50 (18)	N2—C8—H8A	109.8
C9—N2—C8	112.35 (16)	C7—C8—H8A	109.8
C9—N2—H21	109.1 (17)	N2—C8—H8B	109.8
C8—N2—H21	108.3 (17)	C7—C8—H8B	109.8
C9—N2—H22	107.1 (16)	H8A—C8—H8B	108.2
C8—N2—H22	109.1 (17)	N2—C9—C10	109.9 (2)
H21—N2—H22	111 (2)	N2—C9—H9A	109.7
C2—C1—C6	117.3 (2)	C10—C9—H9A	109.7
C2—C1—N1	121.9 (2)	N2—C9—H9B	109.7
C6—C1—N1	120.7 (2)	C10—C9—H9B	109.7
C3—C2—C1	120.6 (2)	H9A—C9—H9B	108.2
C3—C2—H2	119.7	N1—C10—C9	111.91 (17)
C1—C2—H2	119.7	N1—C10—H10A	109.2
C4—C3—C2	121.7 (3)	C9—C10—H10A	109.2
C4—C3—H3	119.2	N1—C10—H10B	109.2
C2—C3—H3	119.2	C9—C10—H10B	109.2
C3—C4—C5	118.0 (2)	H10A—C10—H10B	107.9
C3—C4—H4	121.0	C11—O2—H2O	111.3 (14)
C5—C4—H4	121.0	O1—C11—O2	125.17 (17)
C4—C5—C6	121.6 (3)	O1—C11—C12	120.99 (17)
C4—C5—H5	119.2	O2—C11—C12	113.83 (16)
C6—C5—H5	119.2	C13—C12—C11	122.85 (17)
C5—C6—C1	120.8 (2)	C13—C12—H12	118.6
C5—C6—H6	119.6	C11—C12—H12	118.6
C1—C6—H6	119.6	C12—C13—C14	123.57 (17)
N1—C7—C8	111.8 (2)	C12—C13—H13	118.2
N1—C7—H7A	109.3	C14—C13—H13	118.2
C8—C7—H7A	109.3	O4—C14—O3	124.92 (16)
N1—C7—H7B	109.3	O4—C14—C13	120.10 (17)
C8—C7—H7B	109.3	O3—C14—C13	114.97 (16)
			
C10—N1—C1—C2	18.6 (3)	C10—N1—C7—C8	60.7 (3)
C7—N1—C1—C2	154.6 (2)	C9—N2—C8—C7	53.1 (3)
C10—N1—C1—C6	−165.3 (2)	N1—C7—C8—N2	−57.3 (3)
C7—N1—C1—C6	−29.3 (3)	C8—N2—C9—C10	−52.9 (2)
C6—C1—C2—C3	−0.4 (4)	C1—N1—C10—C9	79.8 (2)
N1—C1—C2—C3	175.8 (2)	C7—N1—C10—C9	−60.2 (3)
C1—C2—C3—C4	0.5 (4)	N2—C9—C10—N1	56.7 (2)
C2—C3—C4—C5	−0.5 (4)	O1—C11—C12—C13	−10.5 (4)
C3—C4—C5—C6	0.4 (5)	O2—C11—C12—C13	168.7 (2)
C4—C5—C6—C1	−0.3 (5)	C11—C12—C13—C14	−179.4 (2)
C2—C1—C6—C5	0.3 (4)	C12—C13—C14—O4	−9.5 (3)
N1—C1—C6—C5	−176.0 (3)	C12—C13—C14—O3	170.9 (2)
C1—N1—C7—C8	−79.5 (3)		

### 4-Phenylpiperazin-1-ium fumarate (12) Hydrogen-bond geometry (Å, º)

**Table:**

*D*—H···*A*	*D*—H	H···*A*	*D*···*A*	*D*—H···*A*
N2—H21···O4	0.87 (2)	1.88 (2)	2.741 (2)	168 (2)
N2—H22···O1^i^	0.89 (2)	1.89 (2)	2.775 (2)	172 (2)
C7—H7*A*···O2^ii^	0.97	2.51	3.317 (3)	141
C8—H8*B*···O3^iii^	0.97	2.55	3.203 (3)	124
C9—H9*A*···O2^iv^	0.97	2.66	3.318 (3)	126
O2—H2*O*···O3^v^	0.92 (2)	1.54 (2)	2.4610 (18)	174 (2)

Symmetry codes: (i) −*x*+1/2, *y*−1, *z*+1/2; (ii) *x*, *y*−1, *z*+1; (iii) −*x*+1/2, *y*, *z*+1/2; (iv) *x*, *y*−1, *z*; (v) *x*, *y*+1, *z*.
