# Supplementary material for: The structures of eleven (4-phen­yl)piperazinium salts containing organic anions
Source: Acta Crystallogr E Crystallogr Commun. 2022 Sep 22;78(Pt 10):1016–27. doi: 10.1107/S2056989022009057 (PMC9535824; doi:10.1107/S2056989022009057)
Supplement: Supplementary file 24 [file e-78-01016-sup22.docx]

Supplementary Information: Hirschfeld Fingerprint plots for 1 – 12. In all cases both cation and anion were included in the calculation of the Hirschfeld surface.


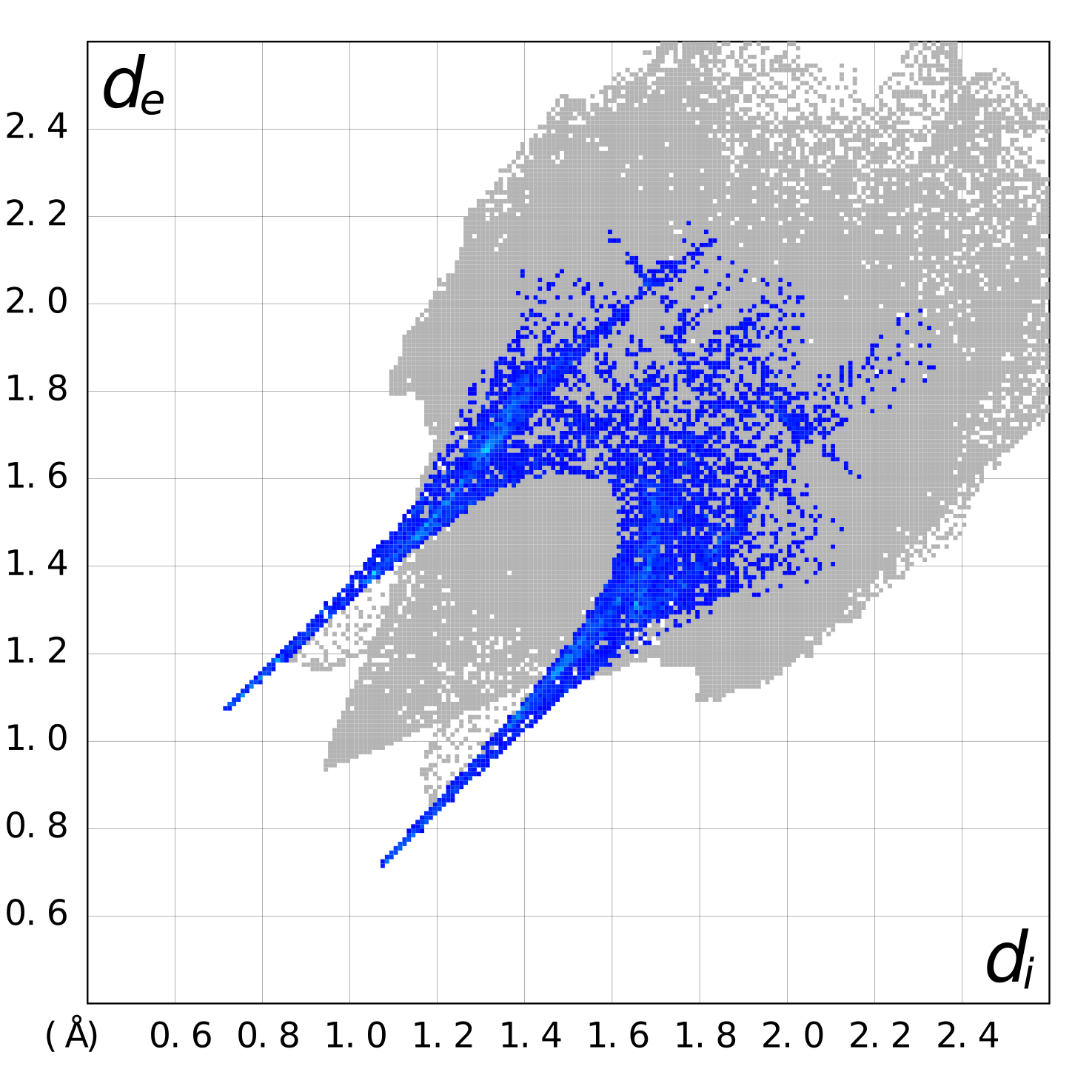


Figure S1. Fingerprint plot for 1. This structure contains H_2_O which is included in the calculation of the surface. No disorder.


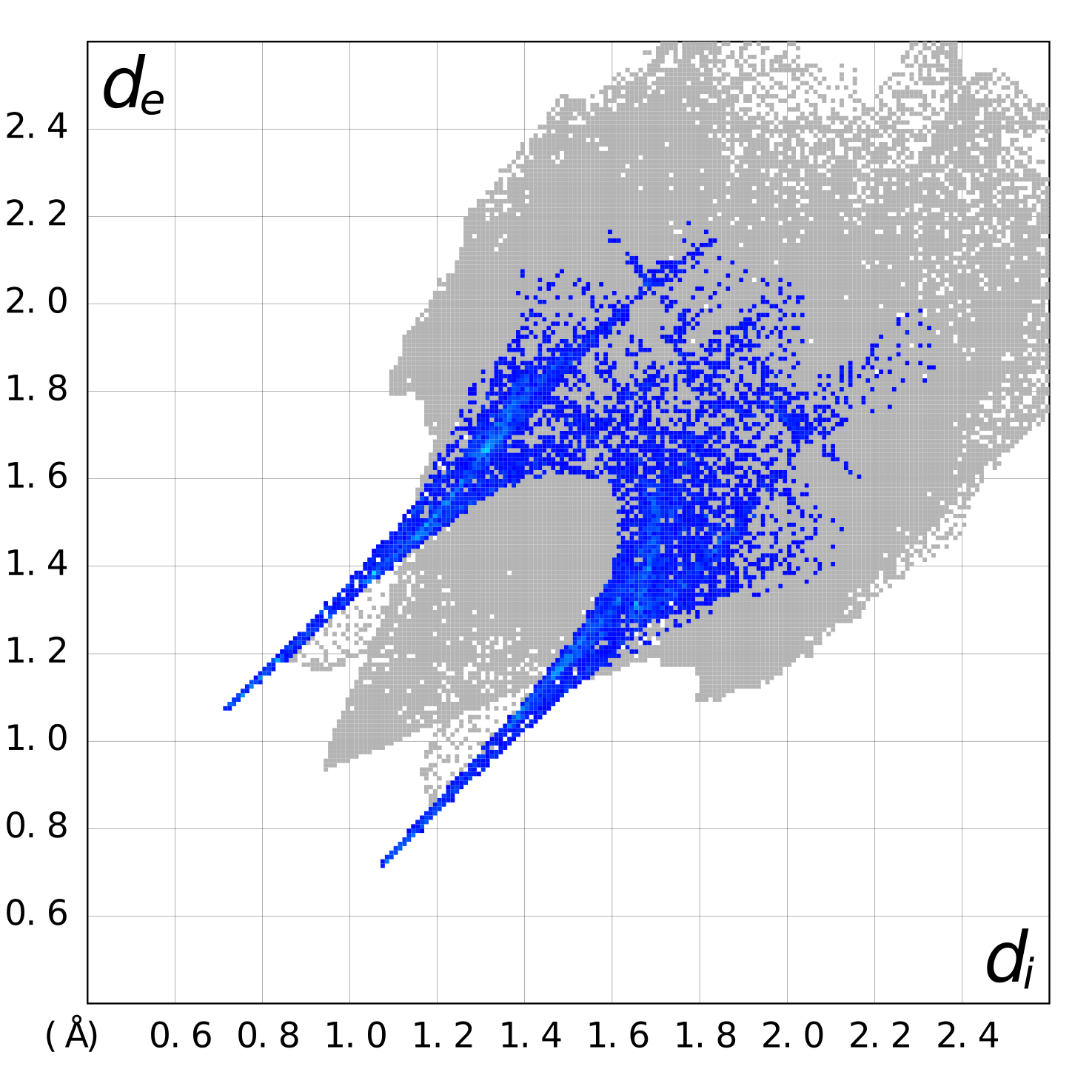


Figure S2. Fingerprint plot for 3. In this structure the Br is disordered over two positions and only the major component was included in the calculation of the surface.


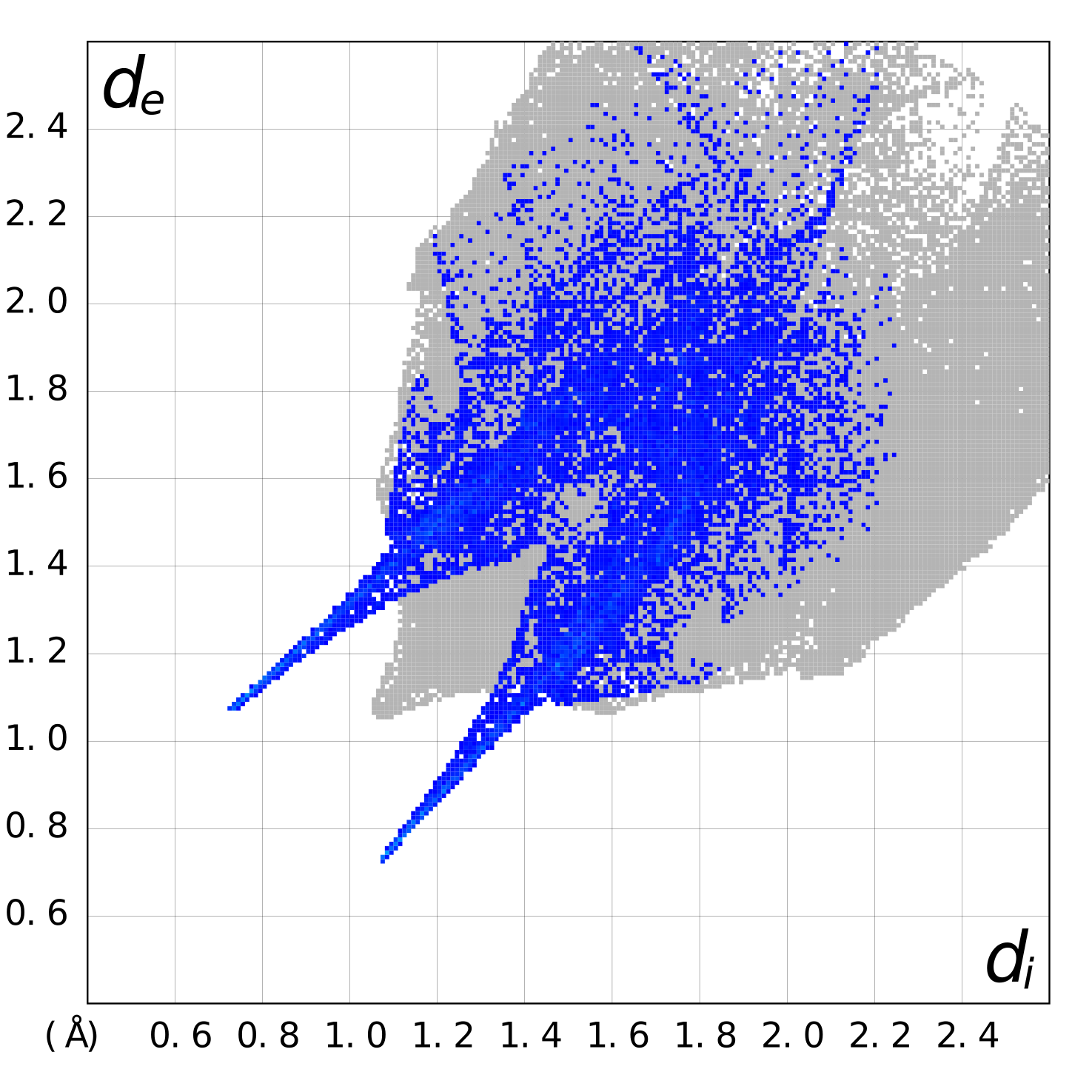


Figure S3. Fingerprint plot for 4. No disorder but there are two formula units in the asymmetric unit and these are included in the calculation of the surface.


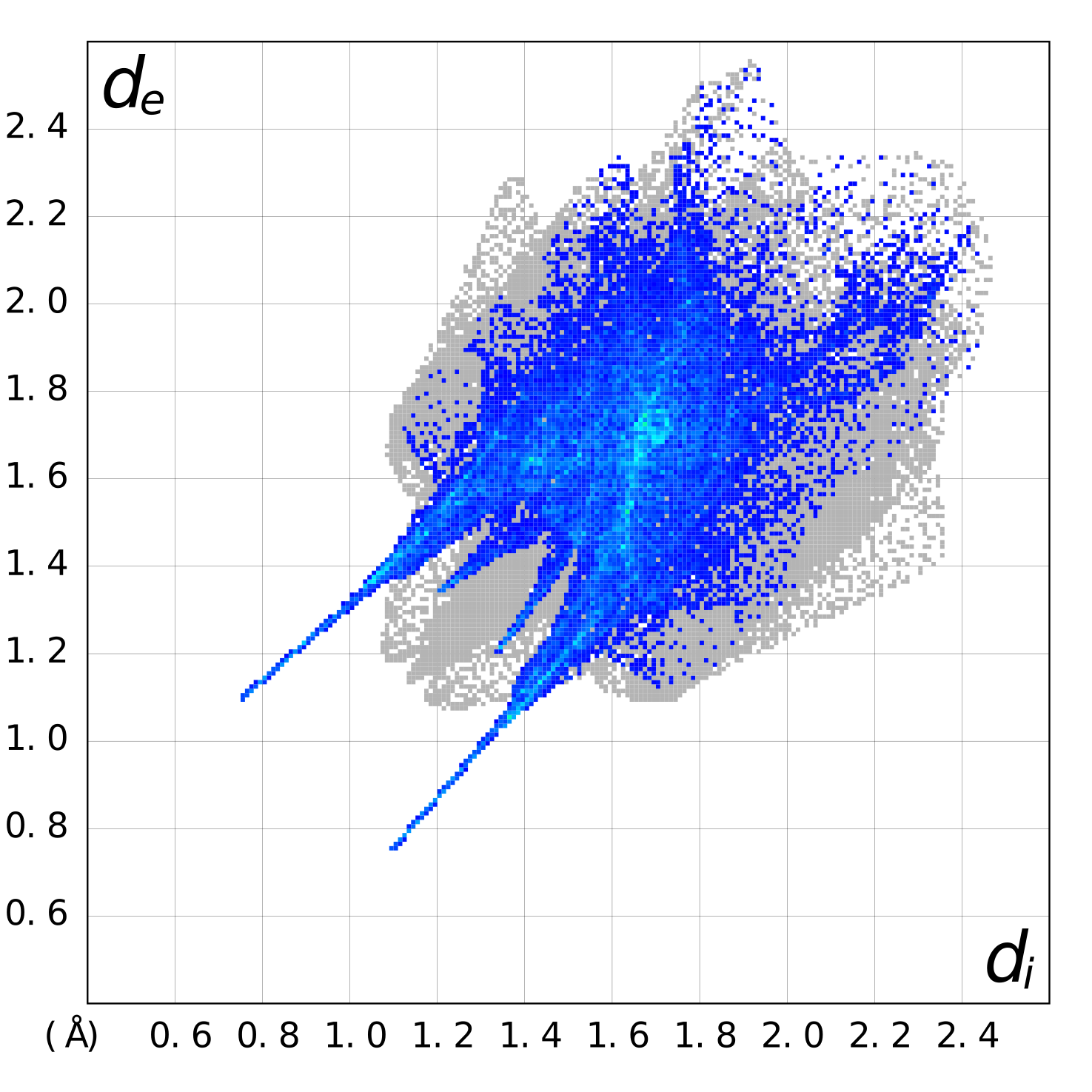


Figure S4. Fingerprint plot for 5. In this case there is disorder in one of the NO_2_ substituents and only the major component was included in the calculation of the surface.


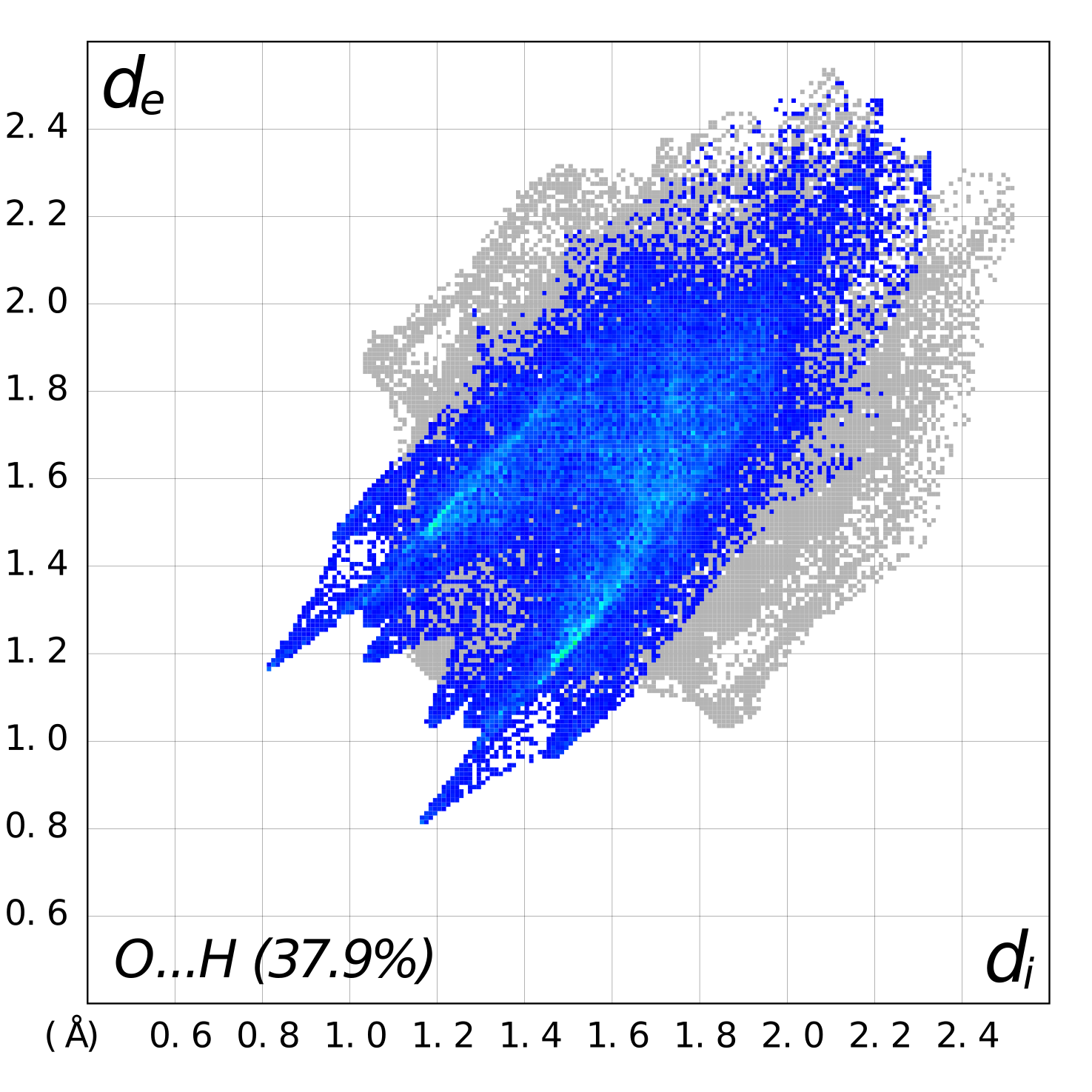


Figure S5. Fingerprint plot for 6. In this case the cation phenyl ring and anion NO_2_ groups were disordered and only the major components were included in the calculation of the surface.


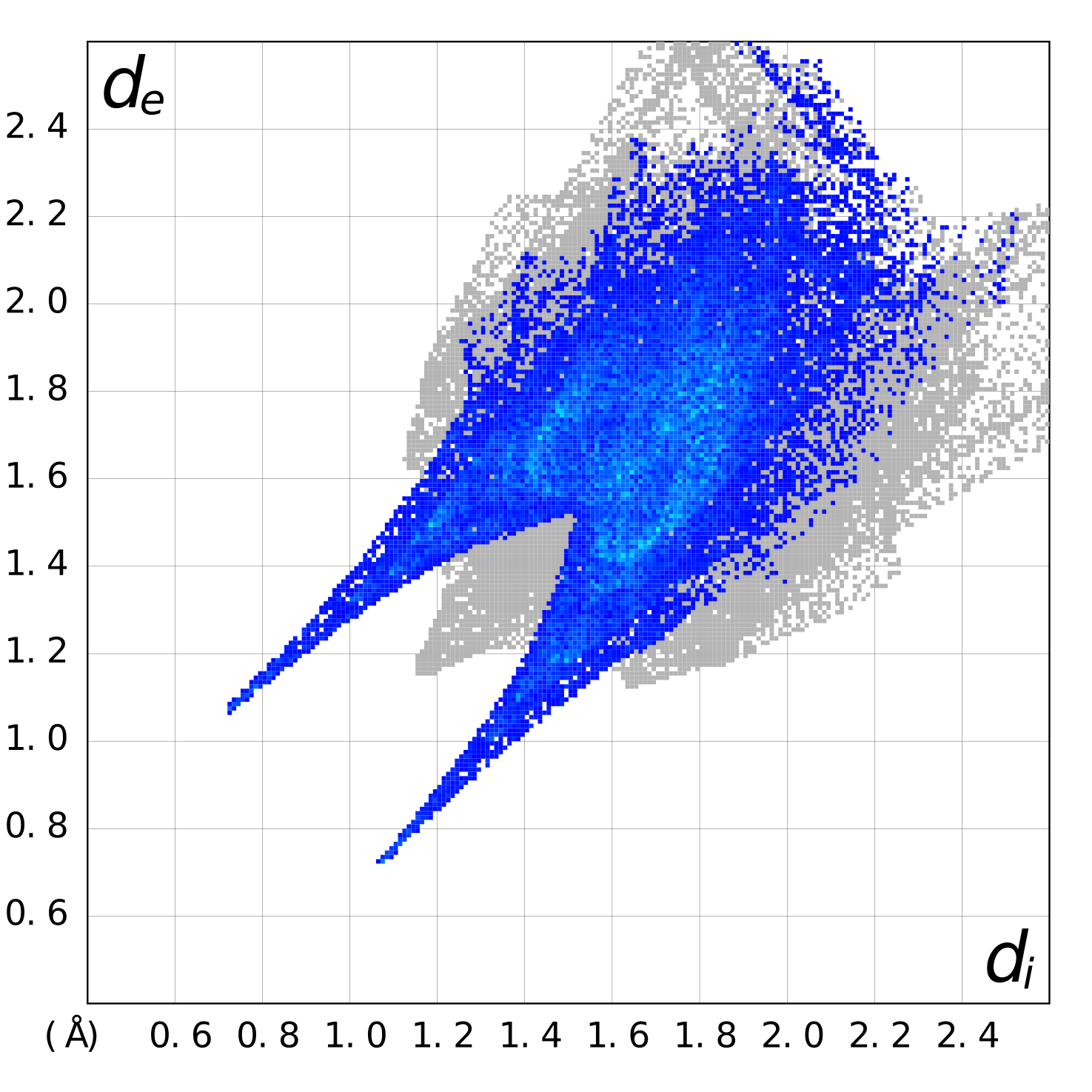


Figure S6. Fingerprint plot for 7. There was no disorder in this structure.


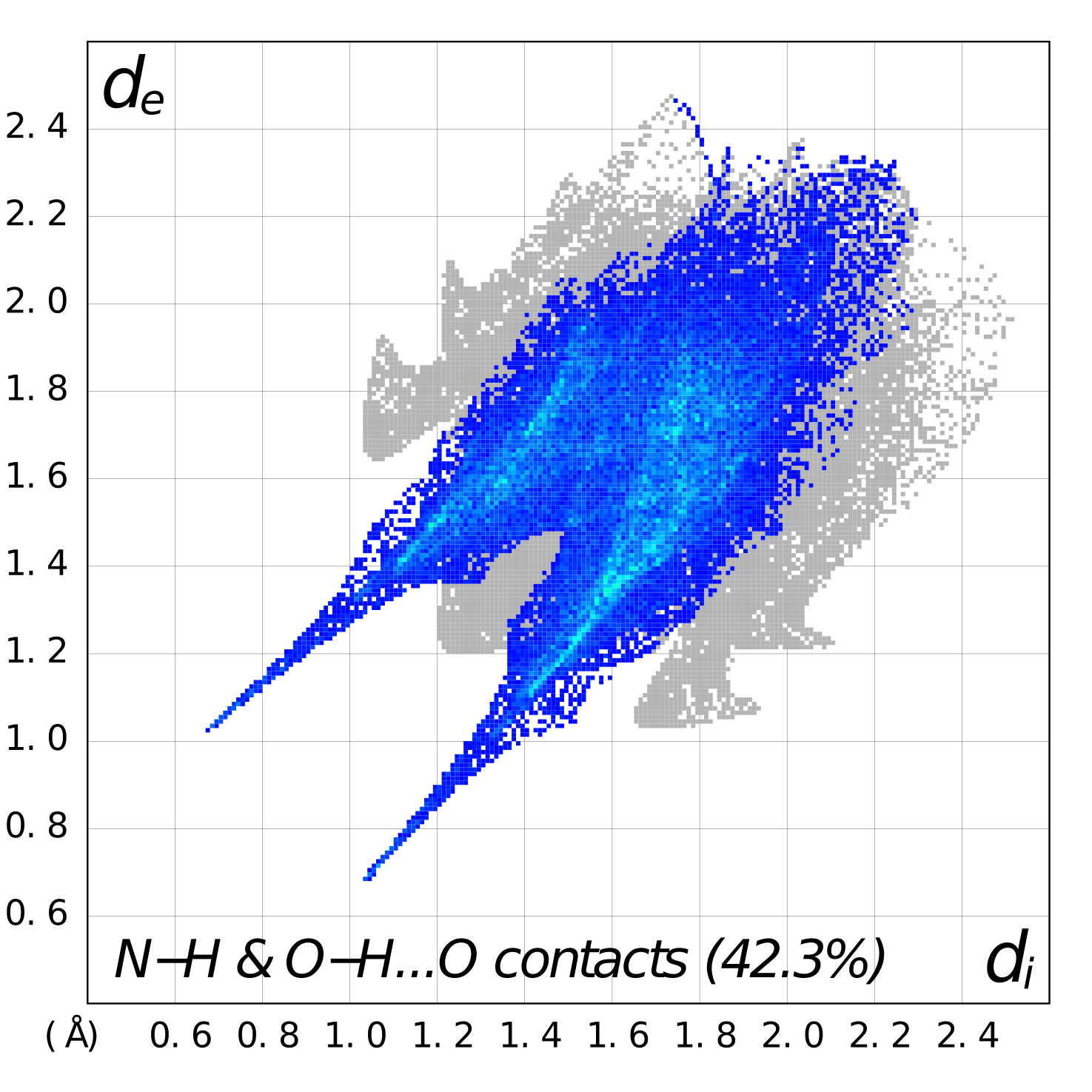


Figure S7. Fingerprint plot for 8. There was no disorder in this structure.


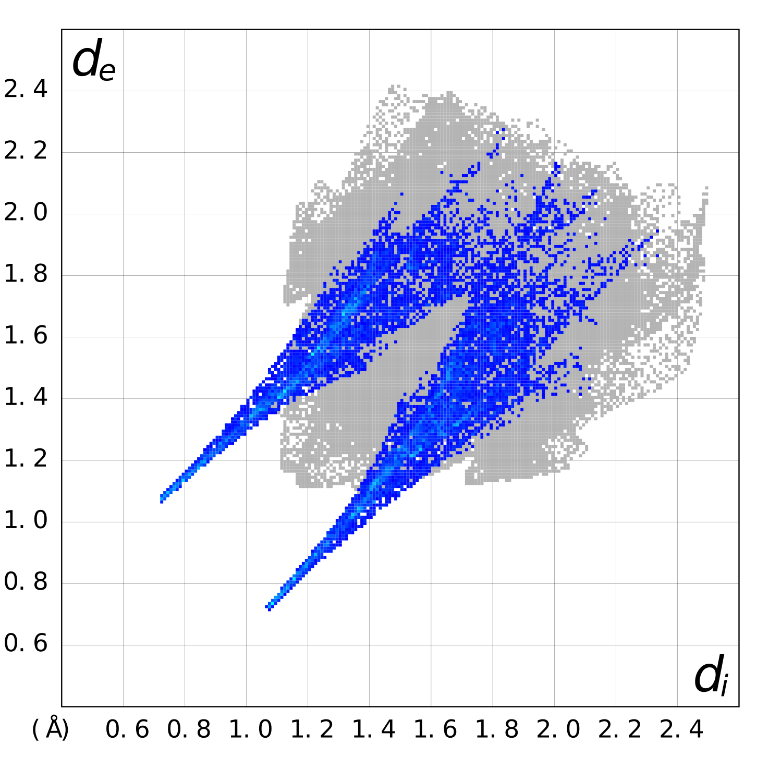


Figure S8. Fingerprint plot for 9. This structure contains H_2_O which is included in the calculation of the surface. No disorder.


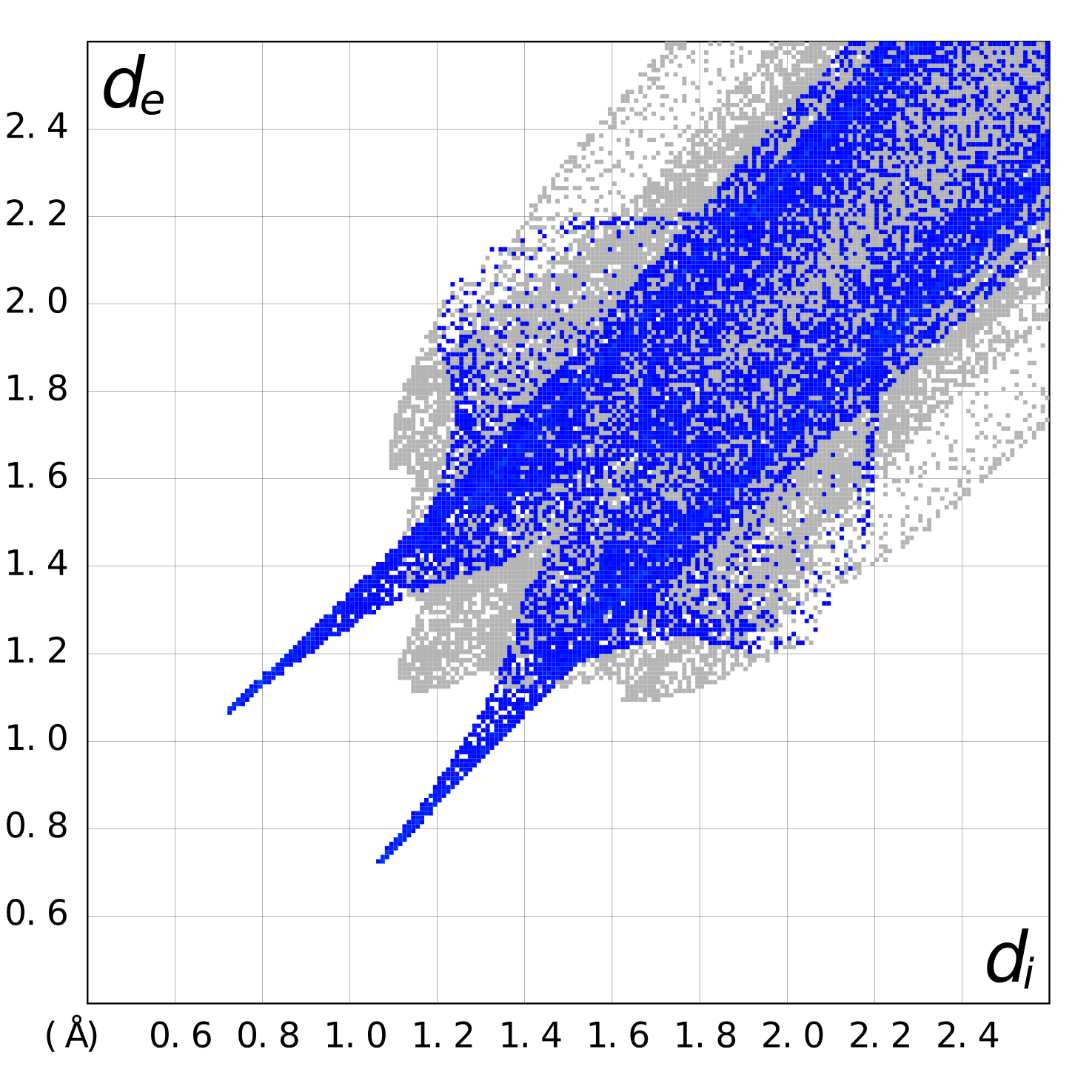


Figure S9. Fingerprint plot for 10. There are two formula units in the asymmetric unit and these are included in the calculation of the surface. In both cations the phenyl ring is disordered and only the major components were included in the calculation of the surface.


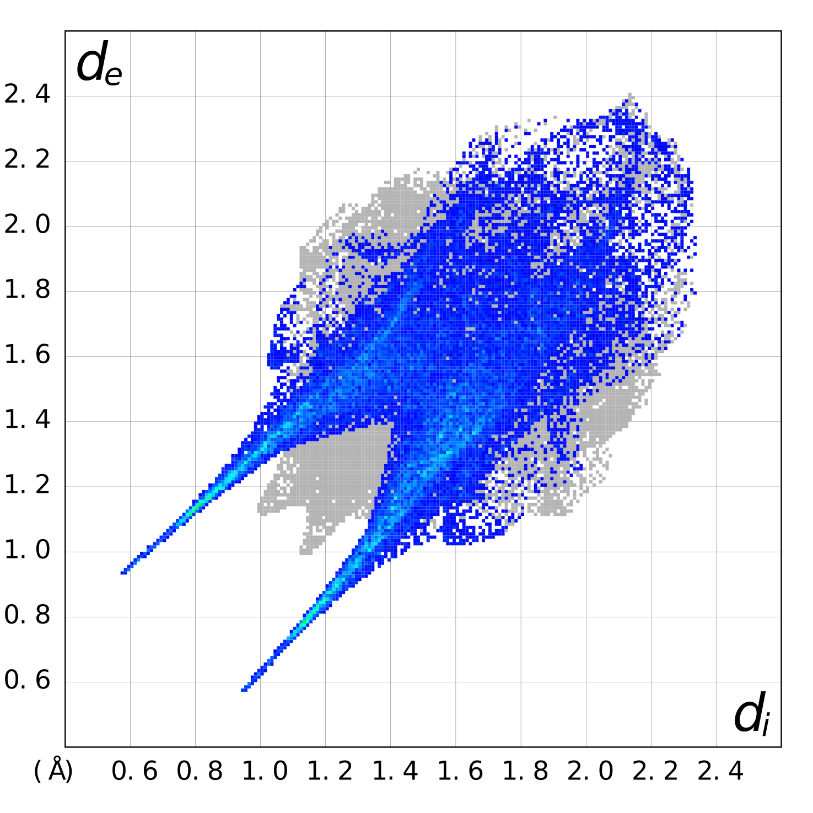


Figure S10. Fingerprint plot for 11. This structure contains H_2_O which is included in the calculation of the surface. For the cation the phenyl ring is disordered and only the major component was included in the calculation of the surface.


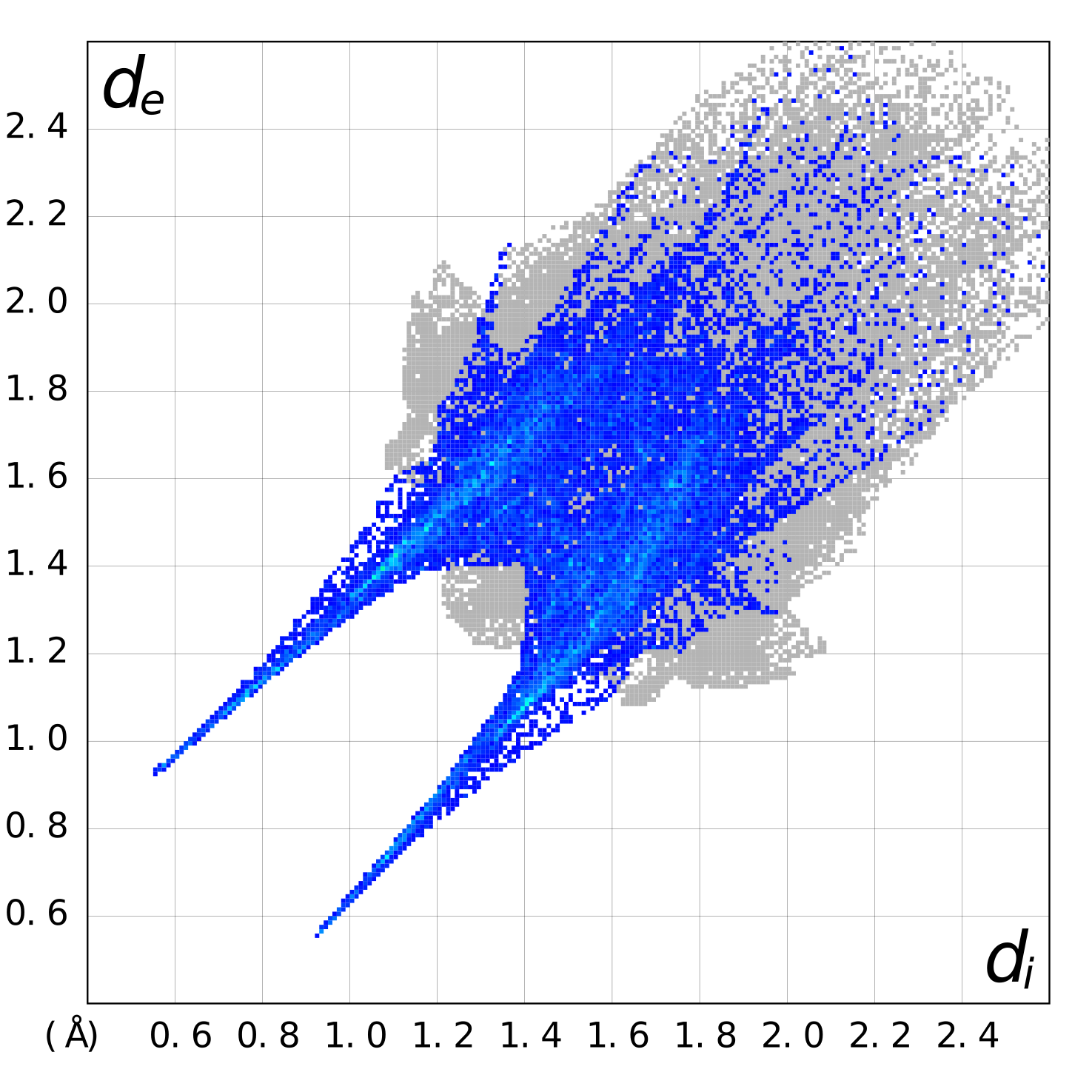


Figure S10. Fingerprint plot for 12. No disorder.
